# Genos: a human-centric genomic foundation model

**DOI:** 10.1093/gigascience/giaf132

**Published:** 2026-01-01

**Authors:** Adi Lin, Bin Xie, Cheng Ye, Cheng Wang, Duoyuan Chen, Ercheng Wang, Fanfeng Lu, Guirong Xue, Haiqiang Zhang, Jiajie Zhan, Jianfeng Zhang, Jiangshuan Pang, Jianqiang Liang, Jiawei Lin, Jiaxin Ma, Jie Hu, Jing Ma, Jinni Dong, Jiongzhen Li, Junchen Liu, Junhong Chen, Junyou Li, Kai Ding, Kaiwen Deng, Kui Chen, Lihui Wang, Longqi Liu, Ling Guo, Liwen Xiong, Luhao Yang, Ming Cheng, Nanning Chen, Renzhong Chen, Shanxin Sun, Shaoshuai Li, Shicheng Chen, Shiping Liu, Siwei Xie, Suyan Liu, Tao Zhou, Wangyang Tang, Weiqiang Zhang, Xianyue Jiang, Xianzhi Qi, Xin Jin, Xinjiang Tan, Xinyue Hu, Xun Xu, Xuyang Feng, Yafei Lu, Yifan Gao, Yong Shang, Youzhe He, Yue Yuan, Yufan Wang, Yuqi Liu, Zhan Xiao, Zhangyuan Meng, Zhaorong Li, Zhe Zhao, Zheng Yang, Zilin Wang

**Affiliations:** Genos Team, West Lake District, Hangzhou 310012, China; Genos Team, West Lake District, Hangzhou 310012, China; Genos Team, West Lake District, Hangzhou 310012, China; Genos Team, West Lake District, Hangzhou 310012, China; Genos Team, West Lake District, Hangzhou 310012, China; Genos Team, West Lake District, Hangzhou 310012, China; Genos Team, West Lake District, Hangzhou 310012, China; Genos Team, West Lake District, Hangzhou 310012, China; Genos Team, West Lake District, Hangzhou 310012, China; Genos Team, West Lake District, Hangzhou 310012, China; Genos Team, West Lake District, Hangzhou 310012, China; Genos Team, West Lake District, Hangzhou 310012, China; Genos Team, West Lake District, Hangzhou 310012, China; Genos Team, West Lake District, Hangzhou 310012, China; Genos Team, West Lake District, Hangzhou 310012, China; Genos Team, West Lake District, Hangzhou 310012, China; Genos Team, West Lake District, Hangzhou 310012, China; Genos Team, West Lake District, Hangzhou 310012, China; Genos Team, West Lake District, Hangzhou 310012, China; Genos Team, West Lake District, Hangzhou 310012, China; Genos Team, West Lake District, Hangzhou 310012, China; Genos Team, West Lake District, Hangzhou 310012, China; Genos Team, West Lake District, Hangzhou 310012, China; Genos Team, West Lake District, Hangzhou 310012, China; Genos Team, West Lake District, Hangzhou 310012, China; Genos Team, West Lake District, Hangzhou 310012, China; Genos Team, West Lake District, Hangzhou 310012, China; Genos Team, West Lake District, Hangzhou 310012, China; Genos Team, West Lake District, Hangzhou 310012, China; Genos Team, West Lake District, Hangzhou 310012, China; Genos Team, West Lake District, Hangzhou 310012, China; Genos Team, West Lake District, Hangzhou 310012, China; Genos Team, West Lake District, Hangzhou 310012, China; Genos Team, West Lake District, Hangzhou 310012, China; Genos Team, West Lake District, Hangzhou 310012, China; Genos Team, West Lake District, Hangzhou 310012, China; Genos Team, West Lake District, Hangzhou 310012, China; Genos Team, West Lake District, Hangzhou 310012, China; Genos Team, West Lake District, Hangzhou 310012, China; Genos Team, West Lake District, Hangzhou 310012, China; Genos Team, West Lake District, Hangzhou 310012, China; Genos Team, West Lake District, Hangzhou 310012, China; Genos Team, West Lake District, Hangzhou 310012, China; Genos Team, West Lake District, Hangzhou 310012, China; Genos Team, West Lake District, Hangzhou 310012, China; Genos Team, West Lake District, Hangzhou 310012, China; Genos Team, West Lake District, Hangzhou 310012, China; Genos Team, West Lake District, Hangzhou 310012, China; Genos Team, West Lake District, Hangzhou 310012, China; Genos Team, West Lake District, Hangzhou 310012, China; Genos Team, West Lake District, Hangzhou 310012, China; Genos Team, West Lake District, Hangzhou 310012, China; Genos Team, West Lake District, Hangzhou 310012, China; Genos Team, West Lake District, Hangzhou 310012, China; Genos Team, West Lake District, Hangzhou 310012, China; Genos Team, West Lake District, Hangzhou 310012, China; Genos Team, West Lake District, Hangzhou 310012, China; Genos Team, West Lake District, Hangzhou 310012, China; Genos Team, West Lake District, Hangzhou 310012, China; Genos Team, West Lake District, Hangzhou 310012, China; Genos Team, West Lake District, Hangzhou 310012, China; Genos Team, West Lake District, Hangzhou 310012, China

**Keywords:** foundation model, human genome, mixture of experts

## Abstract

**Background:**

The rapid expansion of human genomic data demands foundation models that manage ultra-long sequences and capture population diversity, limitations common in existing models that lack human-specific representation, and clinical inference efficiency.

**Results:**

Here, we introduce Genos (Genos-1.2B/Genos-10B), a human-centric genomic foundation model engineered for million-basepair sequence modeling. Genos utilizes a large-scale mixture of experts structure, optimized for a 1-Mb context, trained on high-quality human *de novo* assemblies from datasets such as the Human Pangenome Reference Consortium and the Human Genome Structural Variation Consortium, representing diverse global populations. A suite of optimization strategies was implemented to ensure training stability and enhance computational efficiency, which collectively reduces costs and facilitates million-basepair context modeling. Functionally, Genos performs single-nucleotide resolution analysis and dynamically simulates the cascade effects of noncoding variations on RNA expression profiles. In comprehensive evaluations, Genos uniformly surpasses state-of-the-art models on critical human genomics benchmarks and demonstrates robust omics-text cross-modal diagnostic capabilities. We present a systematic technical evaluation and validation of Genos’s architecture, training convergence, and performance across standard benchmarks.

**Conclusions:**

This work provides a reliable technical blueprint and performance benchmark for the development of the next generation of high-efficiency genomic foundation models. Genos model weights, inference code, and usage documentation are publicly available on GitHub (https://github.com/BGI-HangzhouAI/Genos) and Hugging Face Hub (https://huggingface.co/BGI-HangzhouAI).

## Introduction

### The paradigm shift: genomics and foundation models

Genomics research is currently transitioning from an early phase of massive data accumulation to the contemporary era of intelligent analysis and insight extraction. The proliferation of high-throughput sequencing technologies has generated an unprecedented volume of nucleic acid sequence data, making deep learning–based genomic foundation models (GFMs) a crucial computational tool for deciphering the complexity of life. Analogous to large language models (LLMs) in natural language processing, GFMs aim to learn the intrinsic “grammar” and “semantics” of the genome through large-scale pretraining, enabling unified analysis of functional element identification, variant pathogenicity prediction, and phenotype regulatory networks. This technological breakthrough is pivotal for accelerating precision medicine and population health research.

### Significance of Genos in the field

Significant progress has been made in the GFM landscape, with seminal works like EVO2 [1] and AlphaGenome [2] leading the trend toward long-sequence modeling and cross-species generalization. However, when these models are applied to human translational medicine and clinical high-throughput analysis, they encounter 2 core bottlenecks.

Bottleneck 1: The Human-Centric Representational Gap. The OpenGenome2 dataset used by EVO2 prioritizes cross-species coverage over population diversity, leading to systematic bias in the representation of human-specific regulatory elements (e.g., enhancers, promoters) and rare variants. Similarly, AlphaGenome relies on cohorts with limited reference genomes, struggling to accurately capture complex population-specific genetic patterns. This fundamentally restricts the models’ predictive accuracy and generalizability in complex human disease and rare disorder research.

Bottleneck 2: Efficiency and Deployment Challenges for Ultra-Long Sequences. While existing models have achieved context modeling up to the million-basepair (1 Mb) scale, this often incurs prohibitive computational costs. For instance, the 40B-parameter version of EVO2 requires extensive graphics processing unit (GPU) clusters for training and exhibits high inference latency, unsuitable for time-sensitive clinical analysis. Furthermore, specialized architectures often lack modularity, making them incompatible with mainstream cloud computing infrastructures, significantly raising the barrier to deployment and broad application.

Considering the 2 core bottlenecks, we focused our efforts on robust data engineering and an optimized technical architecture. On the data side, we curated a human-centric, multisource dataset, integrating high-quality, haplotype-resolved assemblies from the Human Pangenome Reference Consortium (HPRC) [3–5] and the Human Genome Structural Variation Consortium (HGSVC) [6] to ensure robust cross-ethnic generalizability. Architecturally, we rooted our design in an evolved Transformer framework, augmenting it with a mixture-of-experts (MoE) [7] structure to address the computational challenges of modeling sequences up to a million bases. This was achieved by integrating elements such as rotary position embedding (RoPE) [8] for extreme context lengths and a multiple parallelism strategy (including tensor, pipeline, context, data, and expert parallelism) to ensure stable and efficient large-scale training. This comprehensive work on data and architecture, coupled with extensive training and optimization, culminated in the release of Genos (Genos-1.2B/Genos-10B), a human-centric genomic foundation model.

Genos stands at the forefront of genomic foundation models, playing a pivotal role in the field of genomics. It has the potential to revolutionize multiple aspects of genomic research and its applications. In precision medicine, Genos can analyze an individual’s genomic data to predict disease risks with greater accuracy. For instance, by identifying key genetic markers associated with diseases such as cancer or neurodegenerative disorders, the development of personalized treatment regimens is facilitated. This not only improves the effectiveness of treatment but also reduces the risk of adverse reactions to medications.

In the realm of group health monitoring, by analyzing genomic data from large populations, the model facilitates the precise identification of genetic trends within different ethnic groups, which is crucial for understanding the genetic basis of diseases prevalent in specific populations. These critical genomic insights, which provide the scientific foundation necessary for formulating, can be used to develop targeted preventive measures and health care policies. In developmental biology, Genos can help in understanding the genetic mechanisms underlying embryo development. By analyzing the genomic sequences at different stages of development, researchers can uncover how genes are regulated to drive the formation of various tissues and organs.

### Objectives and core design feature of Genos

The objective for Genos is to provide a genomic intelligence analysis engine characterized by superior accuracy and efficiency, thereby advancing the field into a mass application phase. Genos provides significant methodological advancements.

In data processing, Genos integrates standardized, high-quality data from leading international genomics initiatives, including the HPRC [3–5] and HGSVC [6]. By constructing a multisource, heterogeneous genomic dataset spanning global populations and incorporating hundreds of nearly telomere-to-telomere (T2T) assemblies, Genos achieves robust cross-ethnic generalizability. To ensure the reliability and representativeness of training data, we designed a multistage quality control pipeline that progressively filters out intergenic sequences of varying lengths, many of which contain segmental duplication (SD) regions.

The model’s architecture is rooted in an evolved Transformer [9] framework, augmented by a MoE [7] structure. This design effectively overcomes the long-standing computational challenge associated with modeling sequences that exceed a million bases. The integration of ultra-long sequence parameterization, multidimensional parallel computing, and specialized complementary attention mechanisms allows Genos to perform single-nucleotide resolution modeling on ultra-long sequences. Consequently, this provides a more comprehensive analytical depth, allowing for the precise capture and analysis of fine-scale genetic details across the entire genome.

Functionally, Genos has the core ability to accurately identify key functional elements in the genome. It can deeply analyze the cascade effect of micro-gene variation on the transcriptional regulatory network. This is a significant improvement over traditional methods, which often have limitations in predicting regulatory elements in the noncoding region. Genos is capable of single-nucleotide resolution analysis within ultra-long noncoding regions and can dynamically simulate the cascade effect of variation sites on RNA expression profiles, offering a novel paradigm for molecular mechanism analysis.

## Method

### Data collection and preprocessing

The training data for Genos were curated from multiple high-quality genomic sources, including 231 haplotype-resolved assemblies from the HPRC (release 2), 65 assemblies from the HGSVC, and 21 genomes from the Centre d’Etude du Polymorphisme Humain (CEPH) cohort, along with 2 reference genomes, GRCh38 and CHM13. In total, the dataset comprises 636 high-quality genomes, representing diverse global populations. Each genome sequence was processed using a one-hot tokenizer, with a vocabulary consisting of the 4 canonical nucleotides (A, T, C, G), the undetermined base N, and special tokens, such as <EOD> marking sequence boundaries. No cell type–specific labels, epigenetic features (e.g., histone modifications), or other functional annotations were incorporated during this stage. This ensures that the model learns a general-purpose representation of the human genome, unbiased toward any particular biological context or experimental condition.

Training was performed in 2 major stages. In the pretraining stage, samples from HPRC release 2 were divided into 4 groups at an approximate 3:3:3:1 ratio, corresponding to sequence lengths of 8,192 bp, 32,768 bp, 131,072 bp, and 1,024,000 bp. Within each stage, about one-fourth of the samples had both haplotypes reverse-complemented, while the remaining samples retained the forward strand orientation. Samples from HGSVC and CEPH pedigrees were all processed into 8,192-bp fragments, with one-fourth of them reverse-complemented in the same manner. Both reference genomes (GRCh38 and CHM13) were prepared with both forward and reverse strands at every length scale. To reduce noninformative intergenic content, 8,192-bp fragments excluded regions located more than 5,120 bp away from any gene boundary, while 32,768-bp fragments excluded regions beyond 10,240 bp from gene boundaries. The 4 pretraining datasets were then sequentially introduced to the model by increasing sequence length, resulting in a total of approximately 1.4 trillion (1,400B) tokens. In the subsequent continued pretraining (CPT) stage, the same samples were reshuffled across lengths and strand orientations to generate an additional 2.6 trillion (2,600B) tokens, which were further randomized before being fed into the model.

Crucially, it is important to emphasize that all filtering was discontinued in the subsequent CPT stage. The additional 2.6 trillion tokens used in CPT were generated from the original samples without any intergenic distance-based exclusion. This ensured that the model was extensively exposed to and trained on distal intergenic regions, segmental duplications, transposable elements, and other complex genomic architectures, thereby cultivating a comprehensive understanding of the entire genomic landscape, including essential “negative” background sequences.

### Model architecture design

Genos employs a MoE architecture evolved from the Transformer, characterized by 12 layers, optimized for both performance and efficiency in genomic sequence modeling. The MoE architecture is established to provide intrinsic performance benefits beyond mere computational efficiency, a principle supporting our genomic model’s design. Foundational work demonstrated that MoE models achieve superior perplexity and accuracy over dense baselines under identical computational budgets [10]. This enhanced modeling capability, attributed to expert specialization and conditional computation leveraging a vast parameter space, is consistently validated across large-scale language models [11] and complex data domains [12]. Consequently, we directly adopted this well-established architectural design, and the resulting performance gains observed in our Genos model align with theoretical expectations and the established body of evidence.

**Figure 1:**
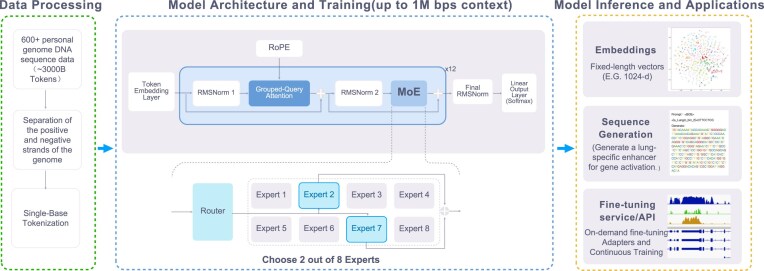
The model architecture of Genos and the design diagram of downstream tasks.

The model begins with a token embedding layer that converts discrete base tokens into continuous vector representations. Following embedding, 3 root mean square normalization (RMSNorm) [13] layers are strategically placed throughout the network to stabilize training by rescaling inputs to have a root mean square of 1, without recentering them around the mean. Between the first and second RMSNorm layers, Genos integrates RoPE [8] with an exceptionally large base frequency of 50,000,000, enabling it to process ultra-long sequences of up to 1 million tokens. Notably, instead of using explicit position embeddings at the input layer, RoPE dynamically injects positional information during attention computation by applying rotary transformations to query and key vectors. This design offers precise positional awareness while supporting extreme context lengths. Complementing RoPE, the model employs a grouped-query attention (GQA) [14] mechanism with 16 attention heads sharing 8 key-value groups. This configuration strikes an optimal balance between computational efficiency and representational capacity, allowing Genos to process long genomic sequences both accurately and efficiently. Genos adopted MoE architecture, which consists of a router network and 8 expert subnetworks. Each expert subnetwork utilizes Swish-Gated Linear Unit (SwiGLU) [15] activation functions, replacing traditional Rectified Linear Unit/Gaussian Error Linear Unit (ReLU/GELU) for improved expressive capability and training stability. The router dynamically selects 2 of the 8 experts for each token based on sequence content, allocating computational resources adaptively (Fig. 1). This design enables efficient processing of both simple repetitive regions and complex regulatory elements. Finally, a linear output layer projects the model’s final hidden state into logits over the vocabulary, where the softmax function then converts them into a probability distribution for the next token, in accordance with the next token prediction (NTP) objective [16]. A key advantage of this model architecture is its inherent flexibility, which enables effective adaptation to various downstream applications.

### Pretraining process and parameter optimization

During the pretraining phase, Genos was trained through the self-supervised paradigm. The model employs the NTP objective while producing general genomic representations.

The model was trained using the Megatron-LM framework [17] across 256 GPUs, employing a sophisticated 5-dimensional parallelism strategy that combines tensor parallelism, pipeline parallelism, context parallelism, data parallelism, and expert parallelism.

Training was conducted with a global batch size of 1,024, achieved via gradient accumulation using a micro-batch size of 1. The optimization process used the AdamW [18] optimizer with a distributed sharded implementation for optimizer states. The learning rate followed a cosine decay schedule, starting with a 5% warm-up phase and peaking at 1e-4, accompanied by gradient clipping set at 1.0 and a weight decay of 0.1.

To address the inherent challenge of expert load imbalance in the MoE architecture—particularly pronounced due to the limited vocabulary of genomic sequences (4 bases)—we implemented an expert load-balancing mechanism with auxiliary loss [10] (coefficient 1e-3). This approach prevents router collapse and ensures uniform activation of experts across diverse genomic contexts. A Z-loss [19] penalty (coefficient 1e-3) applied to router logits prevents numerical instability and ensures smoother training in the MoE components.

To achieve ultra-long context modeling (up to 1 M tokens), we implemented a multistage progressive training strategy. This approach incorporated 3 key technical components: training on data with progressively increasing context lengths, scheduled learning rate decay to effectively mitigate catastrophic forgetting [20], and the application of RoPE-based context window scaling.

To enhance numerical stability and training quality, mixed-precision training was adopted. This involved utilizing BF16 for the majority of computations while strictly retaining FP32 precision for critical operations, specifically (i) the Softmax function within the attention mechanism, (ii) gradient accumulation and All-Reduce communications, and (iii) MoE routing. Simultaneously, reduced-precision matrix multiplication via BF16 was explicitly disabled.

By integrating GQA and Flash Attention [21], Genos capitalizes on their complementary strengths. GQA provides architectural innovations essential for efficient distribution of key-value pairs , while Flash Attention offers an optimized computational kernel for the rapid calculation of attention scores. This synergy established a robust foundation for a high-performance large-scale pretraining model capacity for extensive context windows.

Additional optimizations included grouped general matrix multiplication [22] operations for efficient batched expert computation in MoE layers, all-to-all token dispatching [23] for MoE communication, overlapped parameter gathering and gradient reduction to minimize communication latency [24], and a cyclic data loader with 8 workers to support continuous data streaming during large-scale pretraining.

### Inference and downstream applications

During inference, Genos leverages its adaptive routing mechanism and GQA to efficiently process sequences lengths ranging up to 1 Mb. The model supports 3 primary modalities: embedding generation, sequence generation, and model fine-tuning. For embedding generation, Genos produces fixed-dimensional vector representations that capture biological features for tasks such as sequence clustering and multiomics integration. In sequence generation mode, the model functions as an autoregressive decoder with sampling strategies, including temperature scaling and top-k filtering, to simulate novel sequences or mutated alleles. On-demand fine-tuning via adapter modules or continual learning is available through its hugginface service, enabling customization for specialized tasks such as rare disease variant annotation without requiring full model retraining (Fig. 1). This aims to facilitate deployment across various genomic research and clinical applications.

### Scalable model variants: Genos-1.2B and Genos-10B

**Figure 2:**
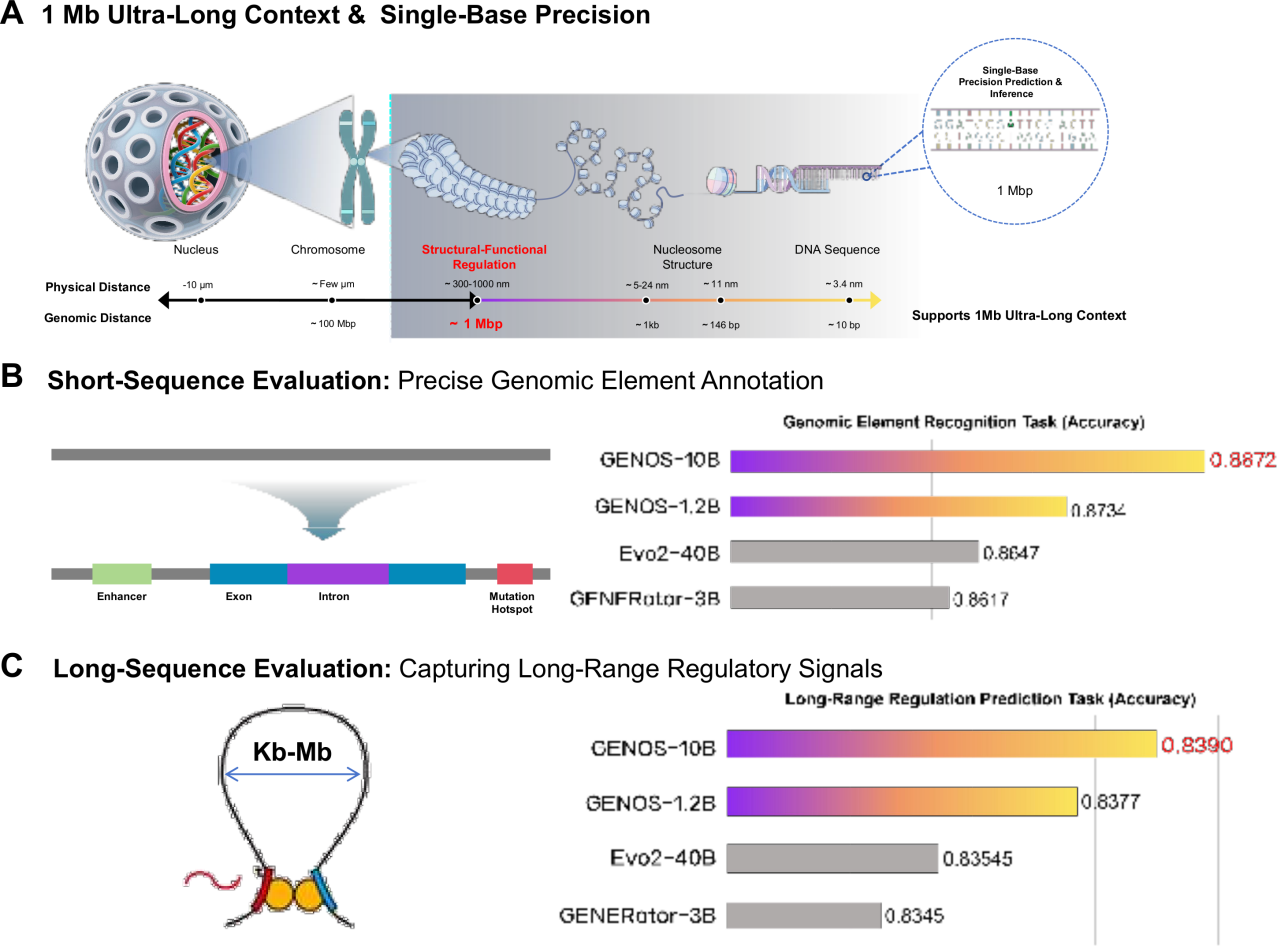
Architectural overview and benchmark performance of the Genos model. (A) Schematic illustration of Genos’s core capabilities: 1-Mb ultra-long context window and single-base precision, enabling the model to analyze genomic sequences from the nucleosomal level down to individual nucleotides for capturing long-range functional regulation. (B) Short-sequence task performance: precise genomic element annotation. Bar plots show the accuracy of Genos and baseline models on tasks, including enhancer, exon, intron, and mutation hotspot recognition. Results are averaged across the respective task categories from the comprehensive benchmark in Table 2. (C) Long-sequence task performance: capturing long-range regulatory signals. Bar plots compare the accuracy of models on predictions requiring the understanding of long-range interactions.

To address diverse computational constraints and application scenarios, we developed 2 versions of the Genos model (1.2B and 10B), with architectural details summarized in Table 1. Compared to the 1.2B variant, the 10B version exhibits substantial improvement across core configuration: the attention hidden dimension scales from 1,024 to 4,096, enhancing contextual understanding; the MoE hidden dimension per expert quadruples from 4,096 to 8,192, boosting representational capacity within each expert subnetwork. Consequently, the total number of parameters rises from 1.25 billion to 10.27 billion, expanding model capacity. Accordingly, the activated parameter count rises from 0.33 billion to 2.87 billion, reflecting the selective utilization inherent in MoE designs. We trained both versions of the model using the same dataset. For this release, the 10B version has been trained on 2,200B tokens, which is slightly higher than the 1,600B tokens used for the 1.2B version. The contrasting scales of the models define their optimal use cases: the 1.2B version is dedicated to resource-constrained analysis and most fine-tuning scenarios, while the 10B version is geared toward intensive, high-capacity modeling requirements (e.g., whole-genome structural variation interpretation). A schematic overview of these key capabilities—single-base resolution and ultra-long context modeling—is provided in Fig. 2A. The model training process was conducted entirely on the 021 Large Science Model and Zero2X open platform.

**Table 1:** Architectural details of 2 versions of Genos model

Version	1.2B	10B
Architecture	MoE	
Number of total parameters	1.25B	10.27B
Number of activated parameters	0.33B	2.87B
Number of layers	12	
Attention hidden dimension	1,024	4,096
MoE hidden dimension (per expert)	4,096	8,192
Number of attention heads	16	
Number of experts	8	
Selected experts per token	2	
Vocabulary size	128 (padded)	256 (padded)
Context length	Up to 1 M	
Attention mechanism	GQA & Flash Attention	
Activation function	SwiGLU	
Trained tokens	1,600B	2,200B

## Performance Evaluation

### Benchmark evaluation and downstream application task

We utilized several standard benchmark datasets to evaluate Genos. We first assessed across a suite of established genomics benchmarks, including the Genomics Benchmark (GB), Nucleotide Transformer Benchmark (NTB), and Genomics Long-Range Benchmark (LRB) datasets [25].

From GB, we selected 3 human-related representative classification tasks: coding versus noncoding sequence discrimination (demo_coding_vs_intergenomic_seqs), enhancer detection (human_enhancers_cohn), and open chromatin region identification (human_ocr_ensembl). From NTB, we included tasks for splice site recognition (splice_sites_all) and histone modification classification (H3, H3K36me3). To evaluate long-range modeling capabilities, 4 LRB human-related tasks were selected, covering enhancer and promoter detection (regulatory_element_enhancer_8 K, regulatory_element_promoter_8 K), as well as prediction of variant effects on expression (variant_effect_causal_eqtl_8 K) and disease pathogenicity (variant_effect_pathogenic_clinvar_8 K).

Tasks from GB and NTB involve relatively short DNA sequences (200–600 bp), while the LRB framework allows arbitrarily long inputs. We generated 8,192-bp (8 K) sequences for all LRB tasks to benchmark long-sequence performance. Dataset splits followed the official configurations or, when unavailable, chromosome-based partitions. In LRB tasks, chromosome 22 was reserved as a validation set. Performance was quantified using the area under the receiver operating characteristic curve (AUC) for binary classification tasks and macro-AUC for multiclass settings.

Next, to further examine scalability to ultra-long inputs, we designed a mutation hotspot classification task using data from the Chinese Pangenome Consortium [26]. Sequences of 8,192 bp (8 K), 32,768 bp (32 K), and 131,072 bp (128 K) were used. Mutation hotspots were identified using a Poisson right-tail test, comparing the mutation count of each sequence to the background mean across all segments within the same chromosome, with significance determined at a false discovery rate <0.05. The dataset was constructed by combining all hotspot sequences with an equal number of randomly selected non-hotspot sequences.

Every evaluation task was performed using the sequence model’s output embeddings as input to a fixed, simple downstream network, enabling direct intermodel comparison.

In addition to the evaluation tasks, we also conducted 2 application-level case studies involving model fine-tuning tailored to specific application requirements. The primary objective here is not to compare intrinsic model capabilities but rather to illustrate the design of feasible downstream applications based on Genos, with the aim of providing case studies for broader practical deployment.

The Encode and Gtex datasets were employed for tasks such as RNA sequencing (RNA-seq) data generation and gene expression analysis. These datasets contain a wealth of single-base transcriptome data from a large number of samples, with different cell types and positive and negative strands labeled. By using these datasets, we could assess Genos’s ability to handle real-world genomic data, learn the underlying patterns in gene expression, and generate accurate predictions.

For evaluating Genos’s performance in tasks related to disease association analysis and gene variation effect prediction, we adopted datasets related to KEGG and Ensembl Variant Effect Predictor (VEP). The KEGG-based dataset contains questions related to chromosome information, pathway networks, reference and variant DNA sequences, and corresponding disease names and reasoning steps. The VEP-based dataset focuses on variant effect prediction questions, reference and variant sequences, and the correct classification of variant effects. These datasets were carefully constructed to cover a wide range of real-world scenarios in genomic research, allowing us to test Genos’s performance in complex and practical genomic analysis tasks.

### Experimental results and comparative analysis

#### Performance comparison with other models

We compared Genos with several other relevant models, including GENERator-3b [27], HyenaDNA-1M [28], NT-2.5b-multi [29], Evo2-7b, and Evo2-40b, across different tasks. Both Genos-1.2B and Genos-10B demonstrated competitive performance over a wide range of topics and input lengths (Table 2).

**Table 2:** Benchmark evaluation of Genos model and other genetic models in various tasks^a^

	Task	Genos 1.2B	Genos 10B	GENERator-3b	HyenaDNA-1M	^b^NT-2.5b-multi	^c^Evo2-7b	^c^Evo2-40b
Short sequence evaluation (sequence length 200–600 bp)	demo_coding_vs_intergenomic_seqs	0.9708	**0.9914**	0.9855	0.9127	0.9763	0.9824	0.9886
	human_enhancers_cohn	**0.8715**	0.8552	0.8181	0.7799	0.7873	0.7733	0.7756
	human_ocr_ensembl	0.7569	0.7623	0.7270	0.6916	0.7285	0.7505	0.7635
	splice_sites_all	0.7819	0.7990	0.8071	0.7110	0.8603	0.8747	0.9138
	H3	0.8944	**0.9400**	0.9163	0.8722	0.9371	0.9140	0.9311
	H3K36me3	0.6883	0.7658	0.8247	0.6787	0.8288	0.8615	0.8823
Mutation hot spot evaluation (sequence length: 8–128 K)	CPC_131072	0.9872	**0.9911**	0.9620	0.9735	/	/	/
	CPC_32768	0.9440	**0.9625**	0.9237	0.9064	/	0.9504	0.9611
	CPC_8192	0.9093	**0.9522**	0.9315	0.8914	/	0.9425	0.9401
Long sequence evaluation (sequence length: 8 K)	regulatory_element_enhancer_8K	0.7469	**0.7532**	0.7390	0.7282	/	0.7454	0.7527
	regulatory_element_promoter_8K	0.9221	0.9249	0.9195	0.8890	/	0.9255	0.9227
	variant_effect_causal_eqtl_8K	0.6990	0.6773	0.6920	0.6887	/	0.7039	0.7054
	variant_effect_pathogenic_clinvar_8K	0.6907	**0.9326**	0.7206	0.6117	/	0.7308	0.9167

The bold numbers indicate the optimal results achieved by Genos in this task.

a Public models of HyenaDNA, Nucleotide Transformer (NT), and other versions in the GENERator series have also been tested. Due to space limitations, the evaluated models not listed include GENERator-1.2b, HyenaDNA-32k, HyenaDNA-450k, NT-500 M, and Evo2-1b. Here, only the best-performing models from each series are shown.

b For NT public models, the maximum acceptable input length is 6,000, making them unavailable for tasks with input lengths of 8 K or more.

c Evo2-7b and Evo-40b models cannot perform inference for 128 K or longer sequences under the HuggingFace framework due to resource constraints.

In short-sequence tasks (200–600 bp) (Table 2), Genos-10B achieved an AUC of 0.9914 on demo_coding_vs_intergenomic_seqs, surpassing models such as GENE-Rator-3B (0.9855), HyenaDNA-1M (0.9127), and NT-2.5B-multi (0.9763). On human_enhancers_cohn, Genos-10B reached an AUC of 0.8552, outperforming NT-2.5B-multi (0.7873) and Evo2-7b (0.7733).

For long-sequence benchmarks (Table 2), Genos-10B achieved an AUC of 0.7532 on regulatory_ element_enhancer_8 K, comparable to the top-performing models. On variant_effect_ pathogenic_clinvar_8 K, it attained an AUC of 0.9326, markedly exceeding GENE-Rator-3B (0.7206) and HyenaDNA-1M (0.6117).

In the mutation hotspot evaluation (8–128 K inputs) (Table 2), Genos-10B consistently achieved the highest performance in AUCs. On CPC_131072, it reached 0.9911, outperforming GENE-Rator-3B (0.9620) and HyenaDNA-1M (0.9735). Similarly, on CPC_32768, it achieved 0.9625, surpassing GENE-Rator-3B (0.9237) and HyenaDNA-1M (0.9064).

Overall, Genos demonstrated strong and consistent performance across benchmarks of varying sequence lengths and biological contexts, highlighting its scalability and robustness from short-range genomic classification to ultra-long sequence modeling (Table 2). The overall performance of Genos and baseline models across fundamental task categories is summarized visually in Fig. 2B, C. The values shown are averaged from the comprehensive per-task metrics presented in Table 2, providing a high-level comparison of model capabilities on short- and long-sequence genomic understanding.

## Case Studies

### RNA-seq profiles prediction case

#### Data preparation and preprocessing steps

In this case, we fine-tune Genos after modifying its output head with a task-specific architecture to predict single-base resolution RNA-seq profiles from DNA sequences across diverse cell types and tissues. In the same way as AlphaGenome​, the training data were sourced from ENCODE [30] and GTEx [31], yielding a total of 667 metadata groups of single-base transcriptome samples. The data were preprocessed by first normalizing all BigWig files to a common scaling factor and then averaging the expression values across samples within each group to generate an average normalized RNA-seq profile for every distinct biological context. The model was trained on paired data, using hg38 reference genome sequences as input and the corresponding averaged RNA-seq profiles as output targets. Considering fine-tuning costs and the consistency of local sequence predictions, we set the sequence window length to 32 kb, with a 16-kb overlap between adjacent windows. Data sampling spanned all positions across chromosomes 1 to 22.

This data preparation and preprocessing strategy aims to provide high-quality and consistent data for subsequent model training, ensuring that the model can effectively learn the underlying relationships between genomic sequences and their corresponding transcriptomic expressions.

#### Network architecture and training process

Full fine-tuning is conducted on the Genos-1.2B model for each RNA-seq profile. The downstream task head employs a convolutional architecture comprising three 1-dimensional convolutional layers, configured with (kernel size, padding, dilation) pairs of (3, 1, 1), (3, 2, 2), and (1, 0, 1), respectively. Channel dimensions are progressively reduced from 1,024 to 256,256 to 64 and 64 to 1. Each convolutional layer is followed by batch normalization, GELU activation, and dropout regularization (dropout rate = 0.1). The final output is scaled via a learnable weight parameter and transformed through a Softplus activation function to enforce nonnegative predictions.

We employed mean squared error (MSE) as the loss function for this token-level regression task. To ensure training stability, we implemented a data scaling strategy similar to AlphaGenome: square root–based smooth clipping and power transformation were applied to compress signal values during training, and the inverse operations were performed during inference.

For optimization, we employed the Adafactor optimizer with a cosine annealing learning rate scheduler and a linear warmup phase covering 5% of the total training steps. The global batch size was set to 256, and each model was trained for 60 epochs totally.

#### Evaluation and result analysis

To assess the fidelity of RNA-seq profile predicted by fine-tuned Genos, we quantified the consistency between model-generated and experimentally derived RNA-seq profiles across 2 cell types: the human B lymphoblastoid cell line (GM12878, EFO:0002784) and natural killer cell (CL:0000623). For each cell type (stratified by DNA strand orientation, “+” or “−”), we calculated log1p-transformed Pearson correlation coefficients across 3 genomic scopes: whole genome, gene region, and gene expression matrix.

As summarized in Table 3, Genos demonstrated strong agreement with experimental RNA-seq results across all scenarios. In GM12878 cells, log1p Pearson correlations reached 0.9335 (whole genome, + strand), 0.9334 (gene region, + strand), and 0.8641 (gene expression, + strand); for the − strand, these values were 0.9182 (whole genome), 0.9274 (gene region), and 0.9081 (gene expression). In natural killer cells, the model achieved correlations of 0.9084 (whole genome, + strand), 0.9036 (gene region, + strand), 0.9267 (gene expression, + strand), and 0.8562 (whole genome, − strand), 0.8542 (gene region, − strand), and 0.8969 (gene expression, − strand).

**Table 3:** Consistency between RNA-seq data of 2 cell types generated by the Genos-1.2B model and the actual results

Type	Cell types	Gene chain	log1p Pearson (whole genome)	log1p Pearson (gene region)	log1p Pearson (gene expression)
Total RNA-seq	GM12878 (EFO:0002784)	+	**0.933467**	0.933387	0.8641
Total RNA-seq	GM12878 (EFO:0002784)	−	0.918187	0.927362	0.9081
Total RNA-seq	Natural killer cell (CL:0000623)	+	0.908418	0.903551	0.9267
Total RNA-seq	Natural killer cell (CL:0000623)	−	0.856171	0.854174	0.8969

The bold numbers indicate the optimal results achieved by Genos in this task.

**Figure 3:**
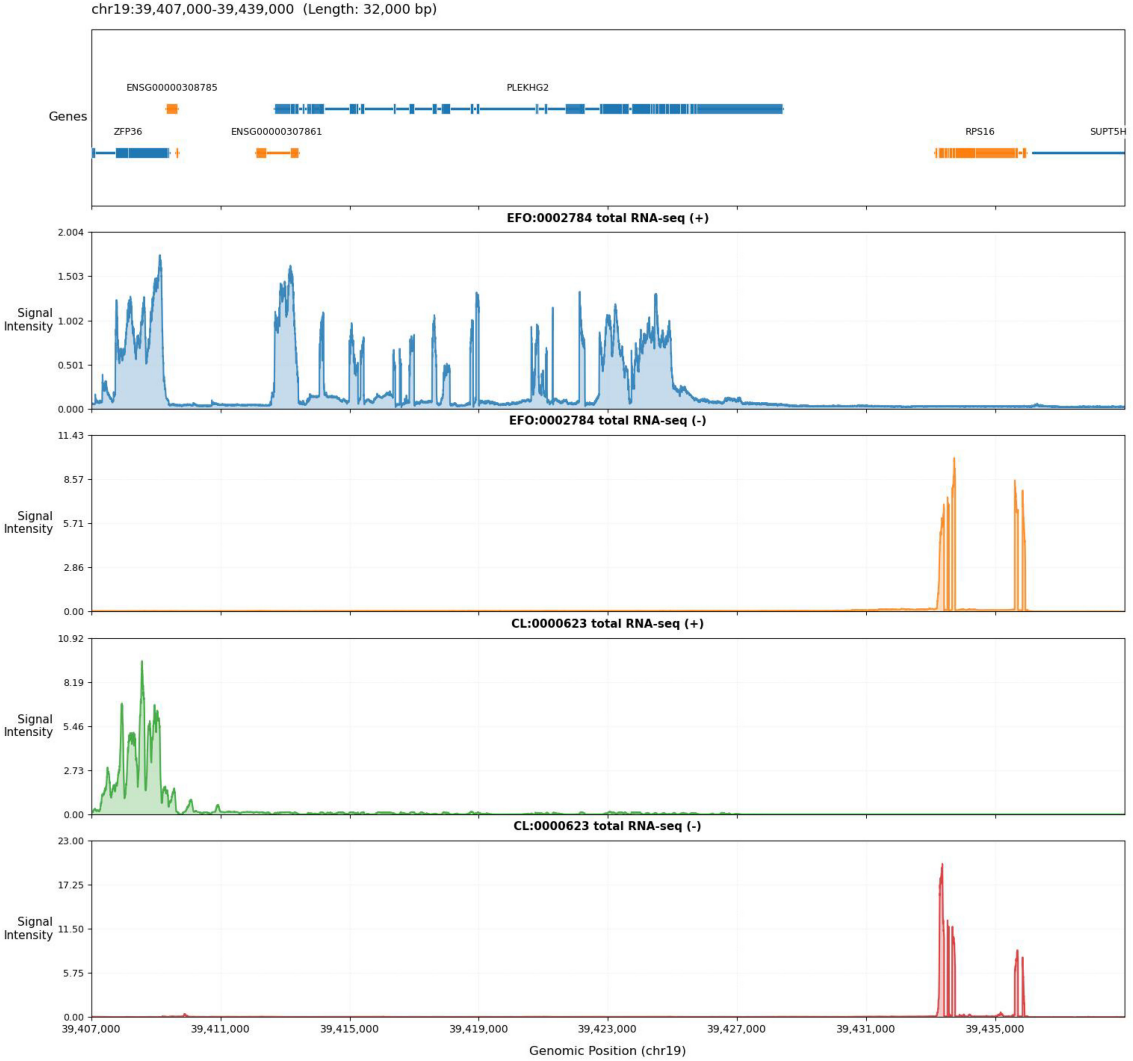
Visualized portion of RNA-seq data for 2 cell types generated by the Genos model

These high correlation values are further corroborated by visual inspection of RNA-seq signal tracks (Fig. 3). The figure illustrates a 32-kb genomic region (chr19:39,407,000–39,439,000) with annotated genes (e.g., ZPBP, RPL36C2, MPL) at the top. Different-colored tracks represent Genos-generated total RNA-seq signals for EFO:0002784 (human B lymphoblastoid cell line GM12878) (blue tracks) and CL:0000623 (natural killer cell) (orange/green/red tracks) across positive (+) and negative (−) strands. For GM12878 (positive strand), signal peaks align precisely with the exonic regions of RPL36C2, reflecting strand-specific transcriptional activity. In natural killer cells, signals concentrate near the MPL locus, and their strand orientation matches the known transcriptional direction of MPL transcripts. These visual patterns confirm that Genos not only achieves high quantitative correlation (Table 3) but also recapitulates cell type–specific and strand-specific transcriptomic landscapes, with signal distributions that accurately mirror gene structure and cell type–dependent expression patterns.

Overall, both quantitative and visual evidence validate Genos as a reliable tool for *in silico* RNA-seq data generation, capturing both global transcriptional patterns (evidenced by whole-genome and gene-region correlations) and gene-specific expression dynamics (reflected in gene expression matrix correlations and signal track alignments). While slight reductions in correlation within gene expression analyses may reflect residual challenges in modeling fine-grained transcriptomic variation, the strong performance across modalities supports the model’s utility for transcriptomic research.

It is noteworthy that our preliminary fine-tuning of the larger Genos-10B parameter model on this task, though currently limited to chromosome 19, already indicates a performance superior to the specialized model AlphaGenome (Supplementary Fig. S1 and Supplementary Table S1). As the genome-wide fine-tuning for the 10B model is ongoing and not yet ready for full release, we conservatively report the results of the fully evaluated Genos-1.2B model in the main text.

### Text-genome model fusion case

#### Project overview and data

To evaluate the performance of a multimodal large language model (combining a genome model and a text model) for predicting genetic diseases caused by gene variants, we follow the architecture [32], which is capable of processing raw DNA sequences while leveraging the reasoning capabilities of large language models to generate biologically consistent explanations and predictions.

**Figure 4:**
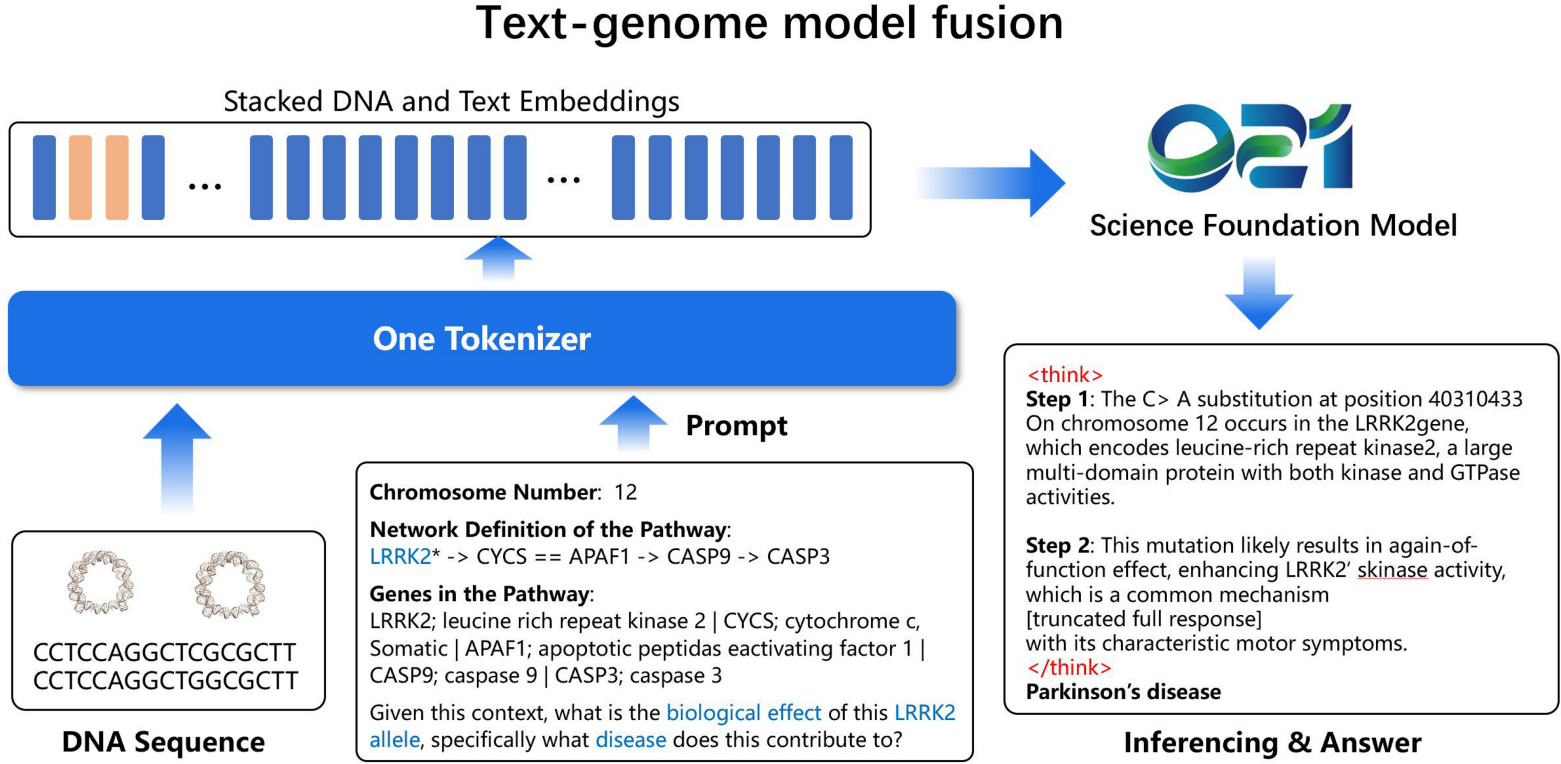
Architecture design of Genos model + text model.

The data used to show the text-genome model fusion are derived from the KEGG task introduced in the Bioreason paper [32]. This task integrates KEGG pathway information with clinical mutation data through a multistage pipeline, employing a standardized symbolic system to represent various molecular interactions and providing reference sequences for comparison with mutated sequences (Fig. 4). The KEGG dataset, comprising 1,449 entries spanning 37 distinct diseases, is partitioned into training, validation, and test sets in an 8:1:1 ratio. Each input consists of a problem description, reference gene sequences, and corresponding mutated gene sequences. The outputs include both reasoning steps and disease classification predictions.

#### Data preprocessing and model training

The data processing workflow adheres to the KEGG processing steps described in Bioreason [32]. The maximum DNA sequence length is limited to 1,024 bp. The goal of this model training is to achieve efficient alignment between DNA sequences and natural language. We utilized 2 series of text models. The first is the Qwen3 [33] series, which includes the Qwen3-1B, Qwen3-4B, and Qwen3-8B models. The second is the 021 Science Foundation Model, which is a large language model trained on extensive scientific corpora with profound scientific cognition. The model training used the AdamW optimizer with a learning rate of 5 × 10⁻⁵ and a weight decay of 1 × 10⁻². Gradient accumulation was set to 8 steps, and a random seed of 23 was used for reproducibility. LoRA adapters were applied with a rank of 32, an α value of 64, and a dropout rate of 0.05. Fine-tuning was performed on the text model (text_model_finetune: True), while the DNA model was frozen. These settings were designed to optimize the training process, balancing model accuracy and computational efficiency through proper regularization and parameter tuning.

#### Evaluation indicator scheme and results

To evaluate the multilabel classification task, we employed standard metrics, including accuracy, macro precision, macro recall, and macro F1-score. These metrics were selected to assess multilabel disease prediction performance, while accounting for potential class imbalance. The results indicate that model performance varied across different architectures and input combinations. For instance, among genome-only models, the **Genos-10B** model achieved an accuracy of **92.07%**, a macro F1-score of **72.59%**, a macro precision of **75.46%**, and a macro recall of **74.15%**. As shown in Table 4, in the genome-text models, the **Genos-1.2B + 021-8B** model achieved an accuracy of **98.28%** and a macro F1-score of **90.37%**, demonstrating its effectiveness in processing raw DNA sequences and accurately predicting disease outcomes.

**Table 4:** Evaluation indicators of the Genos model + text model diagnostic model

Model_type	Model	Accuracy	F1-score	Precision	Recall
Genome	Genos-10B	**92.07%**	72.59%	75.46%	74.15%
	Genos-1.2B	91.72%	72.89%	75.24%	72.95%
	Evo2-1.2b	88.28%	72.43%	75.23%	69.83%
	NT-2.5b-multi	86.55%	69.76%	73.23%	66.62%
	HyenaDNA-1M	50.00%	11.11%	9.22%	14.98%
Genome-text	Genos-1.2B + 021-8B	**98.28%**	90.37%	97.87%	90.15%
	Evo2-1.2b + 021-8B	97.59%	90.96%	98.49%	90.82%
	HyenaDNA-1M + Qwen3-8B	97.58%	95.61%	100%	94.79%
	Evo2-1.2b + Qwen3-4B	97.24%	86.30%	86.75%	87.25%
	Genos-10B + 021-8B	**97.23%**	92.32%	100.00%	90.51%
	Genos-1.2B + Qwen3-4B	**96.90%**	93.24%	100.00%	91.15%
	NT-2.5b-multi + Qwen4B	96.90%	89.03%	90.99%	89.38%
	HyenaDNA-1M + 021-8B	96.55%	93.34%	97.03%	92.86%
	Evo2-1.2b + Qwen3-8B	96.21%	93.53%	100.00%	91.47%
	Genos-10B + Qwen3-4B	**96.21%**	91.75%	100.00%	90.30%
	HyenaDNA-1M + Qwen3-4B	96.21%	89.55%	99.94%	87.02%
	HyenaDNA-1M + Qwen3-1B	93.45%	93.12%	99.05%	91.29%
	Genos-1.2B + Qwen3-1B	91.38%	83.08%	99.57%	79.50%
	Evo2-1.2b + Qwen3-1B	90.42%	75.62%	77.42%	73.91%
	Genos-10B + Qwen1B	88.97%	79.24%	98.22%	76.99%
	NT-2.5b-multi + Qwen1B	88.42%	72.13%	75.42%	71.91%

The bold numbers indicate the optimal results achieved by Genos in this task.

## Deployment and Application Prospects

### Current deployment status and usage

Currently, Genos is in the R&D and optimization phase. It is mainly supporting internal scientific research, providing a powerful tool for researchers within the organization to conduct in-depth genomic studies. Genos is designed to be highly adaptable to mainstream GPU environments, with no special hardware restrictions. This compatibility ensures that it can be easily integrated into existing research setups, reducing the barriers to its utilization.

Adhering to the concept of open science, Genos has deployed cloud reasoning services on the DCS-Cloud platform, thereby constructing a “cloud lab” for genomic intelligence analysis. This open-ecology initiative has far-reaching implications.

Researchers can upload their data through an intuitive interface. Once the data are uploaded, Genos can perform a full-process analysis, starting from mutation function annotation. Mutation function annotation helps in understanding the biological significance of genetic mutations, whether they are benign, pathogenic, or have some other functional implications. The analysis then extends to phenotype prediction, which is a crucial step in connecting genetic information to observable traits. This decentralized computing power support model breaks the shackles of local computing power and algorithm deployment limitations. Researchers from all over the world can now share the predictive power of this leading-edge model. For example, a research team in a resource-limited region can access Genos through the cloud service, enabling them to conduct high-level genomic analysis that was previously out of reach due to lack of local computational resources. This accelerates the transition from genomic discovery to clinical applications, as more research can be carried out and validated, bringing genomic insights closer to patient care.

### Future application potential in biomedicine

In the field of precision medicine, Genos holds great promise. It can analyze an individual’s genomic data to identify disease-related genetic markers with high precision. For example, in cancer diagnosis, Genos can analyze tumor-associated genomic variations, predict the aggressiveness of the cancer, and suggest personalized treatment plans. By accurately predicting the response of different patients to various drugs based on their genetic makeup, Genos can help doctors select the most effective treatment options, minimizing the risk of adverse reactions and improving treatment outcomes.

For group health monitoring, Genos can analyze the genomic data of a large population. It can identify genetic factors associated with common diseases in the population, such as cardiovascular diseases, diabetes, and neurodegenerative disorders. This information can be used to develop preventive strategies, such as targeted health education, lifestyle interventions, and early-detection screening programs for high-risk individuals.

In developmental biology, Genos can contribute to understanding the genetic basis of embryo development. By analyzing the genomic changes during different stages of embryo development, it can uncover the regulatory mechanisms that control cell differentiation, organ formation, and overall development. This knowledge can help in diagnosing and treating developmental disorders and also provide insights into reproductive medicine, such as improving in vitro fertilization techniques. As Genos continues to optimize and iterate, its application potential in these biomedical fields will continue to expand, laying a solid foundation for the development of a more comprehensive and effective health care system.

## Conclusion

### Summary of research findings

Genos represents a significant advancement in genomic intelligence analysis. Specifically, its MoE architecture effectively addresses the computational challenges inherent in ultra-long sequence modeling at single-nucleotide resolution. By introducing strategies such as ultra-long sequence parameterization, multidimensional parallel computing, and complementary attention mechanisms, Genos successfully overcomes the limitations of traditional models in handling million-base sequences. The expert load-balancing mechanism, mixed-precision training strategy, and dynamic routing architecture further enhance the model’s training stability and inference efficiency.

In terms of performance, Genos outperforms existing models in various benchmark tasks. In addition to the existing benchmark comparisons, we designed 2 specific tasks focused on ultra-long sequence modeling. Across these tasks, the Genos model exhibited a clear positive correlation between sequence length and prediction accuracy. In contrast, other models were unable to process sequences of such lengths or did not demonstrate this property of performance scaling with increased sequence context. This finding thus provides empirical evidence for the necessity of longer context windows.

The application cases of Genos further validate its utility. In RNA-seq data generation, Genos can accurately predict gene expression levels, as demonstrated by high Pearson correlation coefficients between predicted and true expression values. In the omics + text interactive disease diagnosis project, Genos, when combined with a large-scale language model, achieves high accuracy in gene variation effect prediction and disease association analysis, with accuracy rates reaching up to 99.31% in some cases.

### Limitations and future work

The Genos model has several limitations that must be addressed in future work. First, computational efficiency requires optimization. Although the architecture is designed for effective resource allocation, there is still potential to significantly reduce the computational cost during training and inference, particularly when handling massive datasets. Second, the capability for cross-modal data fusion needs enhancement. While Genos shows initial promise with genomic data, deeper integration of multiomics data, such as proteomics and metabolomics, alongside phenotypic information, is essential to achieve a more comprehensive understanding of complex biological processes and gene–environment interactions.

Future model development will involve continuous training with an increasingly diverse set of genomic data, with the primary objective remaining a deeper comprehension of the human genome and superior performance in corresponding analytical applications. Furthermore, the integration of other multiomics datasets with the Genos genomic model is anticipated to offer substantial benefits for downstream research and practical applications. Additionally, while Genos’s architectural features (e.g., long-context attention and MoE) are designed to facilitate contextual learning, a comprehensive benchmark evaluating its performance across a wide array of human tissues—such as predicting RNA expression or chromatin accessibility in diverse GTEx or ENCODE contexts—remains an important area for future validation and will be a focus of subsequent studies.

### Significance of Genos for genomics development

Genos is expected to have a substantial impact on the trajectory of genomics research. It marks a paradigm shift from traditional data-driven genomics research to a foundation model-based approach. By providing a powerful tool for accurate and efficient genomic analysis, Genos enables researchers to gain deeper insights into the genetic basis of diseases.

Within the domain of precision medicine, Genos may play a crucial role in disease risk prediction, personalized diagnosis, and treatment stratification. Its capacity to analyze genomic data at a high level of accuracy can aid in identifying disease-associated genetic variants, predicting the efficacy of drugs, and developing personalized treatment plans. This may lead to more effective and targeted medical interventions, reducing the cost and side effects associated with traditional treatment methods.

Moreover, Genos contributes to a more comprehensive understanding of life processes. By decoding the intricate genomic information, it paves the way for advancements in fields such as developmental biology, evolutionary biology, and synthetic biology. Overall, Genos is a key step toward realizing the full potential of genomics in improving human health and understanding the mysteries of life.

## Availability of Source Code and Requirements

Project name: Genos

Project homepage: https://github.com/BGI-HangzhouAI/Genos & https://huggingface.co/BGI-HangzhouAI

Operating system(s): Platform independent

Programming language: Python

Other requirements: pytorch 7.1 or higher, transformers 4.52.4 or higher

License: MIT license

## Additional Files

**Supplementary Table S1**. RNA-seq prediction accuracy: Genos-10B vs. AlphaGenome.

**Supplementary Fig. S1**. Comparative visualization of RNA-seq profile predictions on chromosome 19 for Genos-10B and AlphaGenome. The figure presents a visual comparison of predicted RNA-seq signals against the experimental ground truth for 2 distinct gene regions on chromosome 19. Predictions from the Genos-10B model (fine-tuning in progress) are compared to those from the specialized model AlphaGenome, accessed via its API. Blue: Experimental ground truth RNA-seq data. Orange: RNA-seq profile predicted by the Genos-10B model. Green: RNA-seq profile predicted by the AlphaGenome model. (A) Prediction profiles across the JUNB gene locus. (B) Prediction profiles across an extended region encompassing the LENG8.

giaf132_Supplementary

giaf132_Authors_Response_To_Reviewer_Comments_Original_Submission

giaf132_Authors_Response_To_Reviewer_Comments_Revision_1

giaf132_GIGA-D-25-00428_Original_Submission

giaf132_GIGA-D-25-00428_Revision_1

giaf132_GIGA-D-25-00428_Revision_2

giaf132_Reviewer_1_Report_Original_Submission Xing Zheng -- 10/15/2025

giaf132_Reviewer_1_Report_Revision_1 Xing Zheng -- 10/20/2025

giaf132_Reviewer_2_Report_Original_Submission Xiaofei Yang -- 10/16/2025

giaf132_Reviewer_2_Report_Revision_1 Xiaofei Yang -- 10/20/2025

## Abbreviations

AUC: area under the receiver operating characteristic curve; CEPH: Centre d’Etude du Polymorphisme Humain; CPT: continued pretraining; GB: Genomics Benchmark; GFM: genomic foundation model; GPU: graphics processing unit; GQA: grouped-query attention; HGSVC: Human Genome Structural Variation Consortium; HPRC: Human Pangenome Reference Consortium; KEGG: Kyoto Encyclopedia of Genes and Genomes; LLM: large language model; LRB: Long-Range Benchmark; MoE: mixture of experts; MSE: mean squared error; NTB: Nucleotide Transformer Benchmark; NTP: next token prediction; ReLU/GELU: rectified linear unit/Gaussian error linear unit; RMSNorm: root mean square normalization; RNA-seq: RNA sequencing; RoPE: rotary position embedding; SD: segmental duplication; SwiGLU: swish-gated linear unit; T2T: telomere-to-telomere.

## Author Contributions

All authors (A. Lin, B. Xie, C. Ye, C. Wang, D. Chen, E. Wang, F. Lu, G. Xue, H. Zhang, J. Zhan, J. Zhang, J. Pang, J. Liang, J. Lin, J. Ma, J. Hu, J. Ma, J. Dong, J. Li, J. Liu, J. Chen, J. Li, K. Ding, K. Deng, K. Chen, L. Wang, L. Liu, L. Guo, L. Xiong, L. Yang, M. Cheng, N. Chen, R. Chen, S. Sun, S. Li, S. Chen, S. Liu, S. Xie, S. Liu, T. Zhou, W. Tang, W. Zhang, X. Jiang, X. Qi, X. Jin, X. Tan, X. Hu, X. Xu, X. Feng, Y. Lu, Y. Gao, Y. Shang, Y. He, Y. Yuan, Y. Wang, Y. Liu, Z. Xiao, Z. Meng, Z. Li, Z. Zhao, Z. Yang, Z. Wang) contributed to the design, training, and programming of the GenOS model. D. Chen, Z. Li, S. Liu, L. Guo, Z. Xiao, J. Hu, and X. Tan additionally led the writing and revision of the manuscript. All authors read and approved the final version.

## Data Availability

To facilitate reproducible research and community collaboration, all resources for the Genos model are publicly accessible. Pretrained model weights, inference code, and detailed documentation are released on GitHub (https://github.com/BGI-HangzhouAI/Genos) , the Hugging Face Hub (https://huggingface.co/BGI-HangzhouAI) and the Model Scope (https://modelscope.cn/organization/BGI-HangzhouAI). These resources enable researchers to fine-tune Genos for specialized genomic tasks or integrate it into custom bioinformatic workflows. The model is distributed under the MIT license, permitting unrestricted use, modification, and redistribution for both academic and commercial purposes. For users seeking scalable cloud-based inference, Genos is also deployed on the BGI DCS Cloud platform, with dedicated APIs to support end-to-end genomic analysis without local computing infrastructure.
