# Supplementary material for: Genos: a human-centric genomic foundation model
Source: Gigascience. 2026 Jan 1;14:giaf132. doi: 10.1093/gigascience/giaf132 (PMC12755919; doi:10.1093/gigascience/giaf132)
Supplement: giaf132_Supplementary [file giaf132_supplementary.docx]

**Supplementary Table S1** RNA-seq Prediction Accuracy: Genos-10B vs. AlphaGenome

| **Evaluation model  (on chromosome 19 only)** | **Cell Types** | **Genes chain** | **log1p Pearson (Whole genome)** | **log1p Pearson (Gene region)** |
| --- | --- | --- | --- | --- |
| AlphaGenome | GM12878 (EFO:0002784) | + | 0.958 | 0.956 |
|  | GM12878 (EFO:0002784) | - | 0.947 | 0.944 |
|  | natural killer cell (CL:0000623) | + | 0.914 | 0.902 |
|  | natural killer cell (CL:0000623) | - | 0.901 | 0.887 |
| Genos-10B | GM12878 (EFO:0002784) | + | 0.983 | 0.983 |
|  | GM12878 (EFO:0002784) | - | 0.982 | 0.983 |
|  | natural killer cell (CL:0000623) | + | 0.979 | 0.978 |
|  | natural killer cell (CL:0000623) | - | 0.977 | 0.975 |


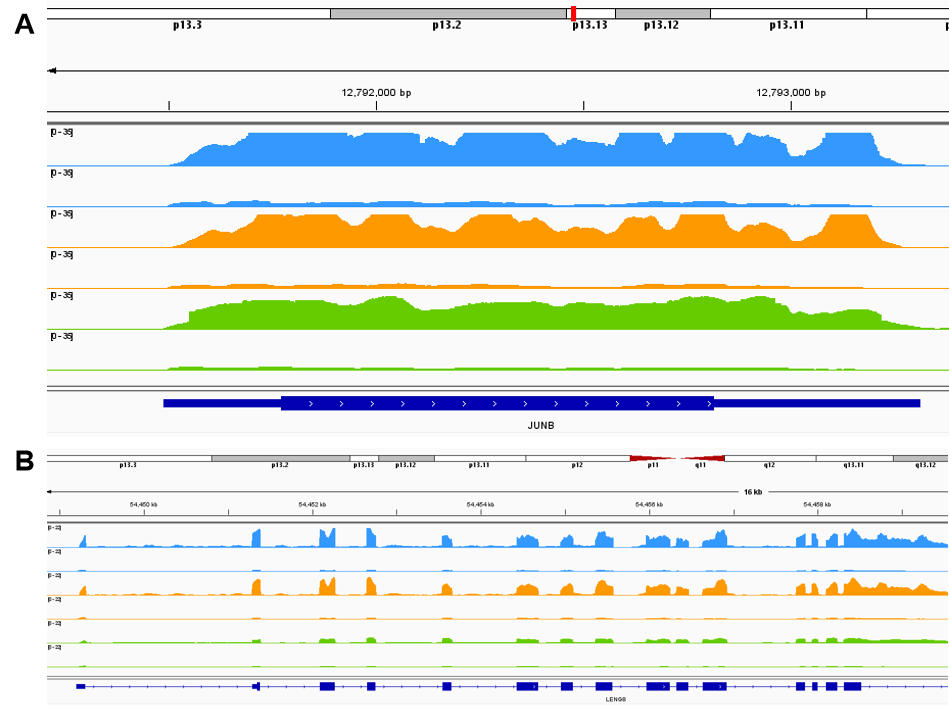


**Supplementary Figure S1 Comparative visualization of RNA-seq profile predictions on chromosome 19 for Genos-10B and AlphaGenome.​**​

The figure presents a visual comparison of predicted RNA-seq signals against the experimental ground truth for two distinct gene regions on chromosome 19. Predictions from the Genos-10B model (fine-tuning in progress) are compared to those from the specialized model AlphaGenome, accessed via its API.​Blue: Experimental ground truth RNA-seq data. Orange: RNA-seq profile predicted by the Genos-10B model. Green: RNA-seq profile predicted by the AlphaGenome model. **(A)** Prediction profiles across the JUNB gene locus **(B)** Prediction profiles across an extended region encompassing the LENG8
