# Supplementary material for: Genos: a human-centric genomic foundation model
Source: Gigascience. 2026 Jan 1;14:giaf132. doi: 10.1093/gigascience/giaf132 (PMC12755919; doi:10.1093/gigascience/giaf132)
Supplement: giaf132_GIGA-D-25-00428_Original_Submission [file giaf132_giga-d-25-00428_original_submission.pdf]

GigaScience

Genos: A Human-Centric Genomic Foundation Model

--Manuscript Draft--

**Manuscript Number:**	GIGA-D-25-00428
**Full Title:**	Genos: A Human-Centric Genomic Foundation Model
**Article Type:**	Research
**Funding Information:**	
**Abstract:**	The rapid expansion of human genomic data demands foundation models that handle long sequences and clinical-grade data, yet existing tools lack human-specific representation and practicality. Here, we present Genos (Genos-1.2B-32K/Genos-10B), a human-centric genomic foundation model trained on high-quality, chromosome-scale de novo assemblies from publicly available datasets such as HPRC and HGSVC, representing diverse global populations. Built on a Transformer-evolved hierarchical hybrid expert (HMoE) architecture, Genos features core innovations: fractal attention for million-base-pair sequence modeling , expert load balancing, and dynamic routing . Genos accurately identifies functional elements, simulates mutation impacts on RNA expression, and breaks non-coding region prediction limitations via a "prediction-interpretation-verification" pipeline. Deployed on BGI DCS Cloud, it offers cloud services eliminating local computing constraints for global researchers. In evaluations, Genos outperforms state-of-the-art models; case studies confirm strong RNA-seq expression correlation and high-accuracy omics-text diagnosis. To advance open science, Genos model weights, inference code, and usage documentation are publicly available on GitHub (https://github.com/BGI-HangzhouAI/Genos) and Hugging Face Hub (https://huggingface.co/BGI-HangzhouAI/Genos), with additional cloud services accessible via BGI DCS Cloud—all released under the MIT License.
**Corresponding Author:**	Duoyuan Chen<br>Genos Team<br>HANGZHOU, CHINA
**Corresponding Author Secondary Information:**	
**Corresponding Author's Institution:**	Genos Team
**Corresponding Author's Secondary Institution:**	
**First Author:**	Duoyuan Chen
**First Author Secondary Information:**	
**Order of Authors:**	Duoyuan Chen
	Shiping Liu
	Zhaorong Li
	Nanning Chen
	Haiqiang Zhang
	Zilin Wang
	Junyou Li
	Youzhe He
	Xinjiang Tan
	Jiajie Zhan
	Shicheng Chen
	Zheng Yang

Powered by Editorial Manager® and ProduXion Manager® from Aries Systems Corporation

	Xinyue Hu
	Wangyang Tang
	Yue Yuan
	Lihui Wang
	Ming Cheng
	Luhao Yang
	Jiongzhen Li
	Yuqi Liu
	Kai Ding
	Cheng Ye
	Jianqiang Liang
	Xianzhi Qi
	Yifan Gao
	Renzhong Chen
	Suyan Liu
	Xuyang Feng
	Yufan Wang
	Kui Chen
	Zhan Xiao
	Kaiwen Deng
	Junchen Liu
	Junhong Chen
	Liwen Xiong
	Jiangshuan Pang
	Bin Xie
	Tao Zhou
	Shaoshuai Li
	Siwei Xie
	Ercheng Wang
	Adi Lin
	Lin Guo
	Jiawei Lin
	Weiqiang Zhang
	Zhe Zhao
	Jie Hu
	Jiaxin Ma
	Jing Ma
	Jianfeng Zhang
	Yong Shang
	Shanxin Sun
	Xun Xu

Powered by Editorial Manager® and ProduXion Manager® from Aries Systems Corporation

Order of Authors Secondary Information:	
**Additional Information:**	
**Question**	**Response**
Are you submitting this manuscript to a special series or article collection?	No
**Experimental design and statistics**<br><br>Full details of the experimental design and statistical methods used should be given in the Methods section, as detailed in our Minimum Standards Reporting Checklist. Information essential to interpreting the data presented should be made available in the figure legends.<br><br>Have you included all the information requested in your manuscript?	Yes
**Resources**<br><br>A description of all resources used, including antibodies, cell lines, animals and software tools, with enough information to allow them to be uniquely identified, should be included in the Methods section. Authors are strongly encouraged to cite Research Resource Identifiers (RRIDs) for antibodies, model organisms and tools, where possible.<br><br>Have you included the information requested as detailed in our Minimum Standards Reporting Checklist?	Yes
**Availability of data and materials**<br><br>All datasets and code on which the conclusions of the paper rely must be either included in your submission or deposited in publicly available repositories (where available and ethically appropriate), referencing such data using a unique identifier in the references and in the "Availability of Data and Materials" section of your manuscript.	Yes

Have you have met the above requirement as detailed in our Minimum Standards Reporting Checklist?	
GigaScience has policies and guidelines in place for the use of generative AI-writing tools such as ChatGPT. If you have used such writing tools to assist with writing the manuscript this must be declared and cited in the text. Authors should not list AI-writing tools and other AI-assisted technologies as an author or co-author and should acknowledge that they are fully responsible for text generated or refined by AI-writing tools.<p><br><br>A summary of use (particularly in the introduction or among methods) needs to be included at the end of the paper, and the outputs should also be included as a supplementary file hosted in GigaDB or other open repositories. Please read our guidelines for more information. <p><br><br>By submitting to GigaScience, you are aware of the journal's AI-writing tools policy, and if you have declared use of such tools below, you have acknowledged this where appropriate in your manuscript and have made a summary of use and outputs available. <p><br><b>AI-assisted writing tools have been used in the preparation of this manuscript?	Yes

Powered by Editorial Manager® and ProduXion Manager® from Aries Systems Corporation

Manuscript
Click here to access/download;Manuscript;[10.13]Genos A Human Centric Genomic Foundation Model-K.docx

Genos: A Human-Centric Genomic Foundation Model

Adi Lin[1], Bin Xie[1], Cheng Ye[1],Cheng Wang[1], Duoyuan Chen[1], Ercheng Wang[1], Fanfeng Lu[1], Guirong Xue[1], Haiqiang Zhang[1], Jiajie Zhan[1], Jianfeng Zhang[1], Jiangshuan Pang[1], Jianqiang Liang[1], Jiawei Lin[1], Jiaxin Ma[1], Jie Hu[1], Jing Ma[1],Jingni Dong[1], Jiongzhen Li, Junchen Liu[1], Junhong Chen[1], Junyou Li[1], Kai Ding[1], Kaiwen Deng[1], Kui Chen[1], Lihui Wang[1], Longqi Liu[1], Ling Guo[1], Liwen Xiong[1], Luhao Yang[1], Ming Cheng[1], Nanning Chen[1], Renzhong Chen[1], Shanxin Sun[1], Shaoshuai Li[1], Shicheng Chen[1], Shiping Liu[1], Siwei Xie[1], Suyan Liu[1], Tao Zhou[1], Wangyang Tang[1], Weiqiang Zhang[1], Xianyue Jiang[1], Xianzhi Qi[1], Xin Jin[1], Xinjiang Tan[1], Xinyue Hu[1], Xun Xu[1], Xuyang Feng[1], Yafei Lu[1] ,Yifan Gao[1], Yong Shang[1], Youzhe He[1], Yue Yuan[1], Yufan Wang[1], Yuqi Liu[1], Zhan Xiao[1], Zhangyuan Meng[1], Zhaorong Li[1], Zhe Zhao[1], Zheng Yang[1], Zilin Wang[1]

[1] Genos team，Hangzhou，China

[**]All authors contributed equally, Authors are ranked in alphabetical order by their first names.

Abstract

The rapid expansion of human genomic data demands foundation models that manage ultra-long sequences and capture population diversity, limitations common in existing models which lack human-specific representation and clinical inference efficiency. Here, we introduce Genos (Genos-1.2B/Genos-10B), a human-centric genomic foundation model engineered for million-base-pair sequence modeling. Genos utilizes a large-sclae Mixture of Experts (MoE) evolved Transformer structure, optimized for a 1Mb context, trained on high-quality human *de novo* assemblies from datasets such as HPRC and HGSVC, representing diverse global populations. This genomic data-optimized model architecture guarantees stable and efficient training, thereby reducing usage costs and enabling long-sequence context capability. Functionally, Genos performs single-nucleotide resolution analysis and dynamically simulates the cascade effects of non-coding variations on RNA expression profiles. In comprehensive evaluations, Genos uniformly surpasses State-of-the-Art models on critical human genomics benchmarks and demonstrates robust Omics-Text cross-modal diagnostic capabilities. We present a systematic technical evaluation and validation of Genos's architecture, training convergence, and performance across standard benchmarks. This work provides a reliable technical blueprint and performance benchmark for the development of the next generation of high-efficiency genomic foundation models. Genos model weights, inference code, and usage documentation are publicly available on GitHub (https://github.com/BGI-HangzhouAI/Genos) and Hugging Face Hub (https://huggingface.co/BGI-HangzhouAI/Genos), with additional cloud services accessible via BGI DCS Cloud—all released under the MIT License.

1. Introduction

1.1 The Paradigm Shift: Genomics and Foundation Models

Genomics research is currently transitioning from an early phase of massive data accumulation to the contemporary era of intelligent analysis and insight extraction. The proliferation of high-throughput sequencing technologies has generated an unprecedented volume of nucleic acid sequence data, making deep learning-based Genomic Foundation Models (GFMs) a crucial computational tool for deciphering the complexity of life. Analogous to Large Language Models (LLMs) in Natural Language Processing, GFMs aim to learn the intrinsic "grammar" and "semantics" of the genome through large-scale pre-training, enabling unified analysis of functional element identification, variant pathogenicity prediction, and phenotyperegulatory networks. This technological breakthrough is pivotal for accelerating precision medicine and population health research.

1.2 Significance of Genos in the Field

Significant progress has been made in the GFM landscape, with seminal works like EVO2(Logsdon et al., 2025) and AlphaGenome(Avsec et al., 2025) leading the trend toward long-sequence modeling and cross-species generalization. However, when these models are applied to human translational medicine and clinical high-throughput analysis, they encounter two core bottlenecks.

Bottleneck I: The Human-Centric Representational Gap. The OpenGenome2 dataset used by EVO2 prioritizes cross-species coverage over population diversity, leading to systematic bias in the representation of human-specific regulatory elements (e.g., enhancers, promoters) and rare variants. Similarly, AlphaGenome relies on cohorts with limited reference genomes, struggling to accurately capture complex population-specific genetic patterns. This fundamentally restricts the models' predictive accuracy and generalizability in complex human disease and rare disorder research.

Bottleneck II: Efficiency and Deployment Challenges for Ultra-Long Sequences. While existing models have achieved context modeling up to the million-base-pair (1Mb) scale, this often incurs prohibitive computational costs. For instance, the 40B-parameter version of EVO2 requires extensive GPU clusters for training and exhibits high inference latency, unsuitable for time-sensitive clinical analysis. Furthermore, specialized architectures often lack modularity, making them incompatible with mainstream cloud computing infrastructures, significantly raising the barrier to deployment and broad application.

To address these two critical bottlenecks, we introduce Genos (Genos-1.2B / Genos-10B), a human-centric GFM designed for high-efficiency long-sequence analysis.

Genos stands at the forefront of genomic foundation models, playing a pivotal role in the field of genomics. It has the potential to revolutionize multiple aspects of genomic research and its applications. In precision medicine, Genos can analyze an individual's genomic data to predict disease risks with greater accuracy. For instance, by identifying key genetic markers associated with diseases such as cancer or neurodegenerative disorders, facilitates the development of personalized treatment regimens. This not only improves the effectiveness of treatment but also reduces the risk of adverse reactions to medications.

In the realm of group health monitoring, by analyzing genomic data from large populations, the model facilitates the precise identification genetic trends within different ethnic groups, which is crucial for understanding the genetic basis of diseases prevalent in specific populations. These critical genomic insights provide the scientific foundation necessary for formulating can be used to develop targeted preventive measures and healthcare policies. In developmental biology, Genos can help in understanding the genetic mechanisms underlying embryo development. By analyzing the genomic sequences at different stages of development, researchers can uncover how genes are regulated to drive the formation of various tissues and organs.

1.3 Objectives and Core Design Feature of Genos

The objective for Genos is to provide a genomic intelligence analysis engine characterized by superior accuracy and efficiency, thereby advancing the field into a mass application phase. Genos provides significant methodological advancements.

In data processing, Genos integrates standardized, high-quality data from leading international genomics initiatives, including the Human Pangenome Reference Consortium (HPRC)(Hickey et al., 2024; Liao et al., 2023; Vollger et al., 2023) and the Human Genome Structural Variation Consortium (HGSVC)(Fairley et al., 2020). By constructing a multi-source, heterogeneous genomic dataset spanning global populations and incorporating hundreds of nearly telomere-to-telomere (T2T) assemblies, Genos achieves robust cross-ethnic generalizability.To ensure the reliability and representativeness of training data, we designed a multi-stage quality control pipeline that progressively filters out intergenic sequences of varying lengths, many of which contain segmental duplication (SD) regions.

The model's architecture is rooted in an evolved Transformer(Vaswani et al., 2017)framework, augmented by a Mixture-of-Experts (MoE) (Jacobs et al., 1991) structure. This design effectively overcomes the long-standing computational challenge associated with modeling sequences that exceed a million bases. The integration of ultra-long sequence parameterization, multi-dimensional parallel computing, and specialized complementary attention mechanisms allows Genos to perform single-nucleotide resolution modeling on ultra-long sequences. Consequently, this provides a more comprehensive analytical depth, allowing for the precise capture and analysis of fine-scale genetic details across the entire genome.

Functionally, Genos has the core ability to accurately identify key functional elements in the genome. It can deeply analyze the cascade effect of micro - gene variation on the transcriptional regulatory network. This is a significant improvement over traditional methods, which often have limitations in predicting regulatory elements in the non - coding region. Genos is capable of single-nucleotide resolution analysis within ultra-long non-coding regions and can dynamically simulate the cascade effect of variation sites on RNA expression profiles, offering a novel paradigm for molecular mechanism analysis.

2. Methodology

2.1 Data Collection and Preprocessing

The training data for Genos were curated from multiple high-quality genomic sources, including 231 haplotype-resolved assemblies from the HPRC (release 2), 65 assemblies from the HGSVC, and 21 genomes from the Centre d'Etude du Polymophisme Humain (CEPH) cohort, along with two reference genomes, GRCh38 and CHM13. In total, the dataset comprises 636 high-quality genomes, representing diverse global populations. Each genome sequence was processed using a one-hot tokenizer, with a vocabulary consisting of the four canonical nucleotides (A, T, C, G), the undetermined base N, and special tokens such as <EOD> marking sequence boundaries.

Training was performed in two major stages. In the pre-training stage, samples from HPRC release 2 were divided into four groups at an approximate 3:3:3:1 ratio, corresponding to sequence lengths of 8,192 bp, 32,768 bp, 131,072 bp, and 1,024,000 bp. Within each stage, about one-quarter of the samples had both haplotypes reverse-complemented, while the remaining samples retained the forward strand orientation. Samples from HGSVC and CEPH pedigrees were all processed into 8,192 bp fragments, with one-quarter of them reverse-complemented in the same manner. Both reference genomes (GRCh38 and CHM13) were prepared with both forward and reverse strands at every length scale. To reduce non-informative intergenic content, 8,192 bp fragments excluded regions located more than 5,120 bp away from any gene boundary, while 32,768 bp fragments excluded regions beyond 10,240 bp from gene boundaries. The four pre-training datasets were then sequentially introduced to the model by increasing sequence length, resulting in a total of approximately 1.4 trillion (1,400B) tokens. In the subsequent continued pre-training (CPT) stage, the same samples were reshuffled across lengths and strand orientations to generate an additional 2.6 trillion (2,600B) tokens, which were further randomized before being fed into the model.

2.2 Model Architecture Design

Genos employs a MoE architecture evolved from the Transformer, characterized by 12 layers, optimized for both performance and efficiency in genomic sequence modeling. The model begins with a token embedding layer that converts discrete base tokens into continuous vector representations. Following embedding, three root mean square normalization (RMSNorm)(Zhang and Sennrich, 2019) layers are strategically placed throughout the network to stabilize training by re-scaling inputs to have a root mean square of one, without re-centering them around the mean. Between the first and second RMSNorm layers, Genos integrates Rotary Position Embedding (RoPE)(Su et al., 2024) with an exceptionally large base frequency of 50,000,000, enabling it to process ultra-long sequences of up to 1 million tokens. Notably, instead of using explicit position embeddings at the input layer, RoPE dynamically injects positional information during attention computation by applying rotary transformations to query and key vectors. This design offers precise positional awareness while supporting extreme context lengths. Complementing RoPE, the model employs a Grouped-Query Attention (GQA) (Ainslie et al., 2023) mechanism with 16 attention heads sharing 8 key-value groups. This configuration strikes an optimal balance between computational efficiency and representational capacity, allowing Genos to process long genomic sequences both accurately and efficiently. Genos adopted MoE archtecture, which consists of a router network and eight expert subnetworks. Each expert subnetwork utilizes SwiGLU(Zhai et al., 2021) activation functions, replacing traditional ReLU/GELU for improved expressive capability and training stability. The router dynamically selects two out of the eight experts for each token based on sequence content, allocating computational resources adaptively (**Figure 1**). This design enables efficient processing of both simple repetitive regions and complex regulatory elements. Finally, a linear output layer projects

the model's final hidden state into logits over the vocabulary, where the softmax function then converts them into a probability distribution for the next token, in accordance with the Next Token Prediction (NTP) objective(Radford and Narasimhan, 2018). A key advantage of this model architecture is its inherent flexibility, which enables effective adaptation to various downstream applications.

2.3 Pre-training Process and Parameter Optimization

During the pre-training phase, Genos is trained through self-supervised paradigms. The model employs the NTP objective while producing general genomic representations.

The model was trained using the Megatron-LM framework (Shoeybi et al., 2019) across 256 GPUs, employing a sophisticated five-dimensional parallelism strategy that combines Tensor Parallelism, Pipeline Parallelism, Context Parallelism, Data Parallelism, and Expert Parallelism.

Training was conducted with a global batch size of 1,024, achieved via gradient accumulation using a micro-batch size of 1. The optimization process used the AdamW (Loshchilov and Hutter, 2017) optimizer with a distributed sharded implementation for optimizer states. The learning rate followed a cosine decay schedule, starting with a 5% warm-up phase and peaking at 1e-4, accompanied by gradient clipping set at 1.0 and weight decay of 0.1.

To address the inherent challenge of expert load imbalance in the MoE architecture—particularly pronounced due to the limited vocabulary of genomic sequences (four bases)—we implemented an expert load balancing mechanism with auxiliary loss (Shazeer et al., 2017) (coefficient 1e-3). This approach prevents router collapse and ensures uniform activation of experts across diverse genomic contexts. A Z-loss (Zoph et al., 2022) penalty (coefficient 1e-3) applied to router logits to prevent numerical instability and ensure smoother training in the MoE components.

To achieve ultra-long context modeling (up to 1M tokens), we implemented a multi-stage progressive training strategy. This approach incorporated three key technical components: training on data with progressively increasing context lengths, scheduled learning rate decay (Wang et al., 2025), to effectively mitigate catastrophic forgetting, and the application of RoPE-based context window scaling.

To enhance numerical stability and training quality, mixed-precision training was adopted. This involving utilizing BF16 for the majority computations while stricted retaining FP32 precision for critical operations, specifically (1) the Softmax function within the attention mechanism, (2) gradient accumulation and All-Reduce communications, and (3) MoE routing. Simultaneously, reduced-precision matrix multiplication via BF16 was explicitly disabled.

By integrating GQA and Flash Attention (Dao et al., 2022), Genos capitalizes on their complementary strengths. GQA provides architectural innovations essential for efficient KV caching, while Flash Attention offers an optimized computational kernel for the rapid calculation of attention scores. This synergy established a robust foundation for a high-performance large-scale pre-training model capacity for extensive context windows.

Additional optimizations included: Grouped GEMM (General Matrix Multiplication) (Hwang et al., 2022)operations for efficient batched expert computation in MoE layers; AllToAll token dispatching for MoE communication; Overlapped parameter gathering and gradient reduction to minimize

communication latency (Liu et al., 2024); A cyclic data loader with 8 workers to support continuous data streaming during large-scale pretraining.

2.4 Inference and Downstream Applications

During inference, Genos leverages its adaptive routing mechanism and GQA to efficiently process sequences lengths ranging up to 1 Mb. The model supports three primary modalities: embedding generation, sequence generation, and model fine-tuning. For embedding generation, Genos produces fixed-dimensional vector representations that capture biological features for tasks such as sequence clustering and multi-omics integration. In sequence generation mode, the model functions as an autoregressive decoder with sampling strategies including temperature scaling and top-k filtering to simulate novel sequences or mutated alleles. On-demand fine-tuning via adapter modules or continual learning is avalable through its hugginface service, enabling customization for specialized tasks such as rare disease variant annotation without requiring full model retraining (**Figure 1**). This aims to facilitate deployment across various genomic research and clinical applications.

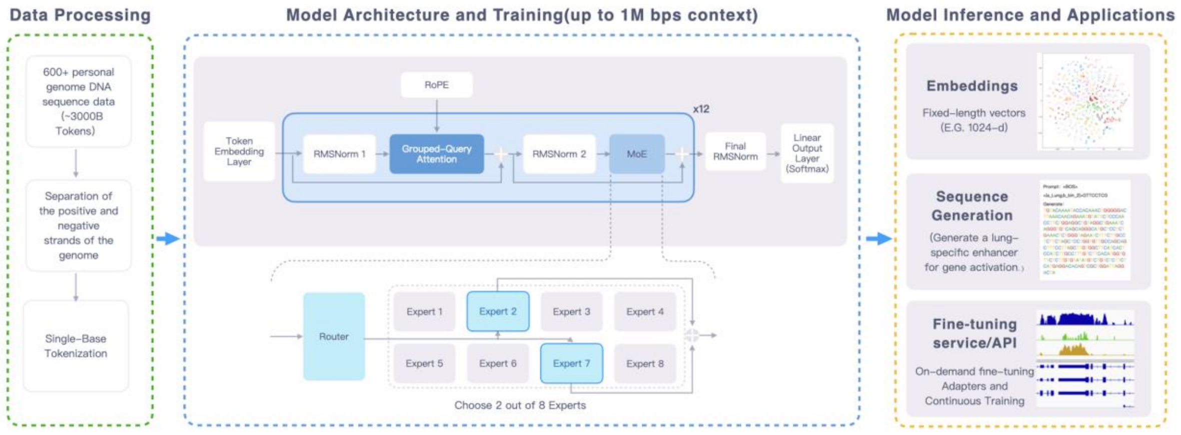

Figure 1 The model architecture of Genos and the design diagram of downstream tasks

2.5 Scalable Model Variants: Genos-1.2B and Genos-10B

To address diverse computational constraints and application scenarios, we developed two versions of the Genos model (1.2B and 10B), with architectural details summarized in **Table 1**. Compared to the 1.2B variant, the 10B version exhibits substantial improvement across core configuration: the total number of parameters rises from 1.25 billion to 10.27 billion, expanding model capacity; consequently, the activated parameter count rises from 0.33 billion to 2.87 billion, reflecting the selective utilization inherent in MoE designs; We trained both versions of the model using the same dataset. For this release, the 10B version has been trained on 2,200B tokens, which is slightly higher than the 1,600B tokens used for the 1.2B version. The contrasting scales of the models define their optimal use cases: the 1.2B version is dedicated to resource-constrained analysis, and most fine-tuning scenarios, while the 10B version is geared towards intensive, high-capacity modeling requirements (e.g., whole-genome structural variation interpretation).

The model training process was conducted entirely on the 021 Large Science Model and Zero2X open platform.

Table 1 Architectural Details of Two Versions of Genos Model

Version	1.2B	10B
**Architecture**	MoE	
**Number of Total Parameters**	1.25B	10.27B
**Number of Activated Parameters**	0.33B	2.87B
**Number of Layers**	12	
**Attention Hidden Dimension**	1024	4096
**MoE Hidden Dimension (per Expert)**	4096	8192
**Number of Attention Heads**	16	
**Number of Experts**	8	
**Selected Experts per Token**	2	
**Vocabulary Size**	128(padded)	256(padded)
**Context Length**	up to 1M	
**Attention Mechanism**	GQA&Flash Attention	
**Activation Function**	SWiGLU	
**Trained Tokens**	1600 B	2200 B

3. Performance Evaluation

3.1 Benchmark Evaluation and Downstream Application Task

We utilized several standard benchmark datasets to evaluate Genos. We firstly assessed across a suite of established genomics benchmarks, including the Genomics Benchmark (GB), Nucleotide Transformer Benchmark (NTB), and Genomics Long-Range Benchmark (LRB) datasets (Trop et al., 2025).

From GB, we selected three human-related representative classification tasks: coding versus noncoding sequence discrimination (demo_coding_vs_intergenomic_seqs), enhancer detection (human_enhancers_cohn), and open chromatin region identification (human_ocr_ensembl). From NTB, we included tasks for splice site recognition (splice_sites_all) and histone modification classification (H3, H3K36me3). To evaluate long-range modeling capabilities, four LRB human-related tasks were selected, covering enhancer and promoter detection (regulatory_element_enhancer_8K, regulatory_element_promoter_8K), as well as prediction of

variant effects on expression (variant_effect_causal_eqtl_8K) and disease pathogenicity (variant_effect_pathogenic_clinvar_8K).

Tasks from GB and NTB involve relatively short DNA sequences (200–600 bp), while the LRB framework allows arbitrarily long inputs. We generated 8,192 bp (8K) sequences for all LRB tasks to benchmark long-sequence performance. Dataset splits followed the official configurations or, when unavailable, chromosome-based partitions. In LRB tasks, chromosome 22 was reserved as a validation set. Performance was quantified using the area under the receiver operating characteristic curve (AUC) for binary classification tasks and macro-AUC for multi-class settings.

Next, to further examine scalability to ultra-long inputs, we designed a mutation hotspot classification task using data from the Chinese Pangenome Consortium (Gao et al., 2023). Sequences of 8,192 bp (8K), 32,768 bp (32K), and 131,072 bp (128K) were used. Mutation hotspots were identified using a Poisson right-tail test, comparing the mutation count of each sequence to the background mean across all segments within the same chromosome, with significance determined at FDR < 0.05. The dataset was constructed by combining all hotspot sequences with an equal number of randomly selected non-hotspot sequences

Every evaluation task was performed using the sequence model's output embeddings as input to a fixed, simple downstream network, enabling direct inter-model comparison.

In addition to the evaluation tasks, we also conducted two application-level case studies involving model fine-tuning tailored to specific application requirements. The primary objective here is not to compare intrinsic model capabilities, but rather to illustrate the design of feasible downstream applications based on Genos, with the aim of providing case studies for broader practical deployment.

The Encode and Gtex datasets were employed for tasks such as RNA - seq data generation and gene expression analysis. These datasets contain a wealth of single - base transcriptome data from a large number of samples, with different cell types and positive and negative strands labeled. By using these datasets, we could assess Genos's ability to handle real - world genomic data, learn the underlying patterns in gene expression, and generate accurate predictions.

For evaluating Genos's performance in tasks related to disease association analysis and gene variation effect prediction, we adopted datasets related to KEGG and VEP. The KEGG - based dataset contains questions related to chromosome information, pathway networks, along with reference and variant DNA sequences, and corresponding disease names and reasoning steps. The VEP - based dataset focuses on variant effect prediction questions, reference and variant sequences, and the correct classification of variant effects. These datasets were carefully constructed to cover a wide range of real - world scenarios in genomic research, allowing us to test Genos's performance in complex and practical genomic analysis tasks.

3.2 Experimental Results and Comparative Analysis

3.2.1 Performance Comparison with Other Models

We compared Genos with several other relevant models, including GENERator - 3b(Wu et al., 2025), HyenaDNA - 1M(Nguyen et al., 2023), NT - 2.5b - multi(Dalla-Torre et al., 2025), Evo2 - 7b, and Evo2 - 40b, across different tasks.Both Genos-1.2B and Genos-10B demonstrated competitive performance over a wide range of topics and input lengths.

In short-sequence tasks (200–600 bp), Genos-10B achieved an AUC of 0.9907 on demo_coding_vs_intergenomic_seqs, surpassing models such as GENE-Rator-3B (0.9855), HyenaDNA-1M (0.9127), and NT-2.5B-multi (0.9763). On human_enhancers_cohn, Genos-10B reached an AUC of 0.8806, outperforming NT-2.5B-multi (0.7873) and Evo2-7B (0.7733).

For long-sequence benchmarks, Genos-10B achieved an AUC of 0.7536 on regulatory_ element_enhancer_8K, comparable to the top-performing models. On variant_effect_ pathogenic_clinvar_8K, it attained an accuracy of 0.9298, markedly exceeding GENE-Rator-3B (0.7206) and HyenaDNA-1M (0.6117).

In the mutation hotspot evaluation (8K–128K inputs), Genos-10B consistently achieved the highest accuracy. On CPC_131072, it reached 0.9886, outperforming GENE-Rator-3B (0.9620) and HyenaDNA-1M (0.9735). Similarly, on CPC_32768, it achieved 0.9720, surpassing GENE-Rator-3B (0.9237) and HyenaDNA-1M (0.9064).

Overall, Genos demonstrated strong and consistent performance across benchmarks of varying sequence lengths and biological contexts, highlighting its scalability and robustness from short-range genomic classification to ultra-long sequence modeling.

Table 2 Benchmark Evaluation of Genos Model and Other Genetic Models in Various Tasks

	**Task**	**Genos 1.2B**	**Genos 10B**	**GENERator-3b**	**HyenaDNA-1M**	***NT-2.5b-multi**	****Evo2-7b**	****Evo2-40b**
Short sequence evaluation (sequence length 200-600bp)	demo_coding_vs_intergenomic_seqs	0.9708	0.9914	0.9855	0.9127	0.9763	0.9824	0.9886
	human_enhancers_cohn	0.8715	0.8565	0.8181	0.7799	0.7873	0.7733	0.7756
	human_ocr_ensembl	0.7569	0.7717	0.7270	0.6916	0.7285	0.7505	0.7635
	splice_sites_all	0.7819	0.8045	0.8071	0.7110	0.8603	0.8747	0.9138
	H3	0.8944	0.9404	0.9163	0.8722	0.9371	0.9140	0.9311
	H3K36me3	0.6883	0.7810	0.8247	0.6787	0.8288	0.8615	0.8823
Mutation hot spot evaluation (sequence length: 8K ~ 128K)	CPC_131072	0.9872		0.9620	0.9735	/	/	/
	CPC_32768	0.9440	0.9645	0.9237	0.9064	/	0.9504	0.9611
	CPC_8192	0.9093	0.9526	0.9315	0.8914	/	0.9425	0.9401
Long sequence evaluation (sequence length: 8K)	regulatory_element_enhancer_8K	0.7469	0.7530	0.7390	0.7282	/	0.7454	0.7527
	regulatory_element_promoter_8K	0.9221	0.9269	0.9195	0.8890	/	0.9255	0.9227
	variant_effect_causal_eqtl_8K	0.6990	0.6712	0.6920	0.6887	/	0.7039	0.7054
	variant_effect_pathogenic_clinvar_8K	0.6907	0.9341	0.7206	0.6117	/	0.7308	0.9167

* Public models of HyenaDNA, Nucleotide Transformer (NT), and other versions in the GENERator series have also been tested. Due to space limitations, the evaluated models not listed include GENERator-1.2b, HyenaDNA-32k, HyenaDNA-450k, NT-500M, and Evo2-1b. Here, only the best-performing models from each series are shown.

** For NT public models, the maximum acceptable input length is 6000, making them unavailable for tasks with input lengths of 8K or more.

*** For Evo2 1b public models, only the base version with an 8K input length is available, making it unusable for tasks requiring longer inputs.

**** Evo2 7b and 40b models cannot perform inference for 128K or longer sequences under the HuggingFace framework due to resource constraints.

4. Case Studies

4.1 RNA-seq Profiles Prediction Case

4.1.1 Data Preparation and Preprocessing Steps

In this case, we fine-tune Genos after modifying its output head with a task-specific architecture to predict single-base resolution RNA-seq profiles from DNA sequences across diverse cell types and tissues. In the same way as AlphaGenome, the training data were sourced from ENCODE (Consortium, 2012) and GTEx (Kim-Hellmuth et al., 2020), yielding a total of 667 metadata groups of single-base transcriptome samples.The data was preprocessed by first normalizing all BigWig files to a common scaling factor and then averaging the expression values across samples within each group to generate an average normalized RNA-seq profile for every distinct biological context. The model was trained on paired data, using hg38 reference genome sequences as input and the corresponding averaged RNA-seq profiles as output targets. Considering fine-tuning costs and the consistency of local sequence predictions, we set the sequence window length to 32 kb, with a 16 kb overlap between adjacent windows. Data sampling spanned all positions across chromosomes 1–22. This data preparation and preprocessing strategy aims to provide high-quality and consistent data for subsequent model training, ensuring that the model can effectively learn the underlying relationships between genomic sequences and their corresponding transcriptomic expressions.

4.1.2 Network Architecture and Training Process

Full fine-tuning is conducted on the Genos-1.2B model for each RNA-seq profile. The downstream task head employs a convolutional architecture comprising three 1D convolutional layers, configured with (kernel size, padding, dilation) pairs of (3, 1, 1), (3, 2, 2), and (1, 0, 1), respectively. Channel dimensions are progressively reduced from 1024 to 256, 256 to 64, and 64 to 1. Each convolutional layer is followed by batch normalization, GELU activation, and dropout regularization (dropout rate = 0.1). The final output is scaled via a learnable weight parameter and transformed through a Softplus activation function to enforce non-negative predictions.
We employed MSE as the loss function for this token-level regression task. To ensure training stability, we implemented a data scaling strategy similar to AlphaGenome: a square-root-based smooth clipping and power transformation were applied to compress signal values during training, and the inverse operations were performed when inference.

For optimization, we employed the Adafactor optimizer with a cosine annealing learning rate scheduler and a linear warmup phase covering 5% of the total training steps. The global batch size was set to 256, and each model was trained for 60 epochs totally.

4.1.3 Evaluation and Result Analysis

To assess the fidelity of RNA-seq profile predicted by fine-tuned Genos, we quantified the consistency between model-generated and experimentally derived RNA-seq profiles across two cell types: the human B lymphoblastoid cell line (GM12878, EFO:0002784) and natural killer cell (CL:0000623). For each cell type (stratified by DNA strand orientation, "+" or "–"), we calculated log1p-transformed Pearson correlation coefficients across three genomic scopes: whole genome, gene region, and gene expression matrix.

As summarized in Table 3, Genos demonstrated strong agreement with experimental RNA-seq results across all scenarios. In GM12878 cells, log1p Pearson correlations reached 0.9335 (whole genome, + strand), 0.9334 (gene region, + strand), and 0.8641 (gene expression, + strand); for the − strand, these values were 0.9182 (whole genome), 0.9274 (gene region), and 0.9081 (gene expression). In natural killer cells, the model achieved correlations of 0.9084 (whole genome, + strand), 0.9036 (gene region, + strand), 0.9267 (gene expression, + strand), and 0.8562 (whole genome, − strand), 0.8542 (gene region, − strand), 0.8969 (gene expression, − strand).

These high correlation values are further corroborated by visual inspection of RNA-seq signal tracks **(Fig. 2)**. The figure illustrates a 32 kb genomic region (chr19:39,407,000–39,439,000) with annotated genes (e.g., ZPBP, RPL36C2, MPL) at the top. Different colored tracks represent Genos-generated total RNA-seq signals for EFO:0002784 (human B lymphoblastoid cell line GM12878) (blue tracks) and CL:0000623 (natural killer cell) (orange/green/red tracks) across positive (+) and negative (−) strands. For GM12878 (positive strand), signal peaks align precisely with the exonic regions of RPL36C2, reflecting strand-specific transcriptional activity. In natural killer cells, signals concentrate near the MPL locus, and their strand orientation matches the known transcriptional direction of MPL transcripts. These visual patterns confirm that Genos not only achieves high quantitative correlation **(Table 3)** but also recapitulates cell type–specific and strand-specific transcriptomic landscapes, with signal distributions that accurately mirror gene structure and cell type–dependent expression patterns.

Overall, both quantitative and visual evidence validate Genos as a reliable tool for in silico RNA-seq data generation, capturing both global transcriptional patterns (evidenced by whole-genome and gene-region correlations) and gene-specific expression dynamics (reflected in gene expression matrix correlations and signal track alignments). While slight reductions in correlation within gene expression analyses may reflect residual challenges in modeling fine-grained transcriptomic variation, the strong performance across modalities supports the model's utility for transcriptomic research.

Table 3 Consistency between RNA-seq data of two cell types generated by the Genos model and the actual results

Type	Cell Types	Genes chain	log1p Pearson (Whole genome)	log1p Pearson (Gene region)	log1p Pearson (Gene expression)
total RNA-seq	GM12878 (EFO:0002784)	+	0.933467	0.933387	0.8641
total RNA-seq	GM12878 (EFO:0002784)	-	0.918187	0.927362	0.9081

total RNA-seq	natural killer cell (CL:0000623)	+	0.908418	0.903551	0.9267
total RNA-seq	natural killer cell (CL:0000623)	-	0.856171	0.854174	0.8969

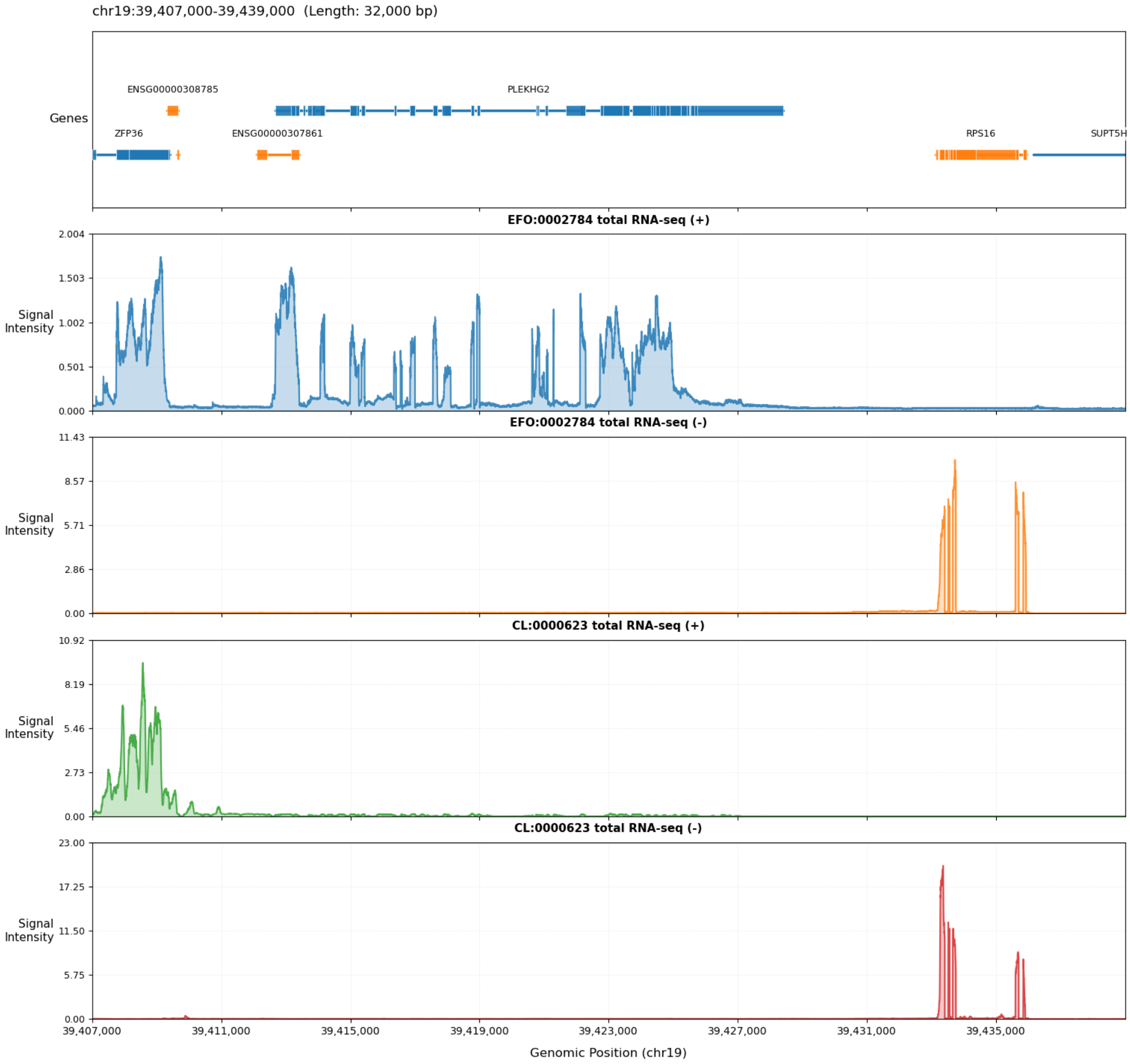

Figure 3 Visualized portion of RNA-seq data for two cell types generated by the Genos model

4.2 Text-genome Model Fusion Case

4.2.1 Project Overview and Data

To validate the performance of a multimodal large language models (gene model + text model) in the task of predicting genetic diseases caused by gene variants it is capable of handling raw DNA

sequences while leveraging the reasoning capabilities of large language models to generate biologically consistent explanations and predictions.

The data comes from the KEGG task in the paper Bioreason (Fallahpour et al., 2025). This task integrates KEGG pathways with clinical database mutation information through a multi-stage process, using a standardized symbolic system to represent various interactions in the molecular network, and providing a reference sequence for comparison with the mutated sequences. The KEGG dataset contains 1,449 entries across 37 different diseases, with the data split into training, validation, and testing sets in a ratio of 8:1:1. The inputs include the problem description, reference gene sequences, and mutated gene sequences. The outputs include reasoning and disease classification predictions.

The model output was in the form of text generation. The model generated text that contained the reasoning process and the final answer. The format was `<|im_start|>assistant [reasoning content] Answer: [final answer]<|im_end|>`. The evaluation results were in CSV format, containing columns such as 'Ground_truth' for the real label, 'Pred_Label' for the prediction label, and 'Generated_text' for the complete generated text.

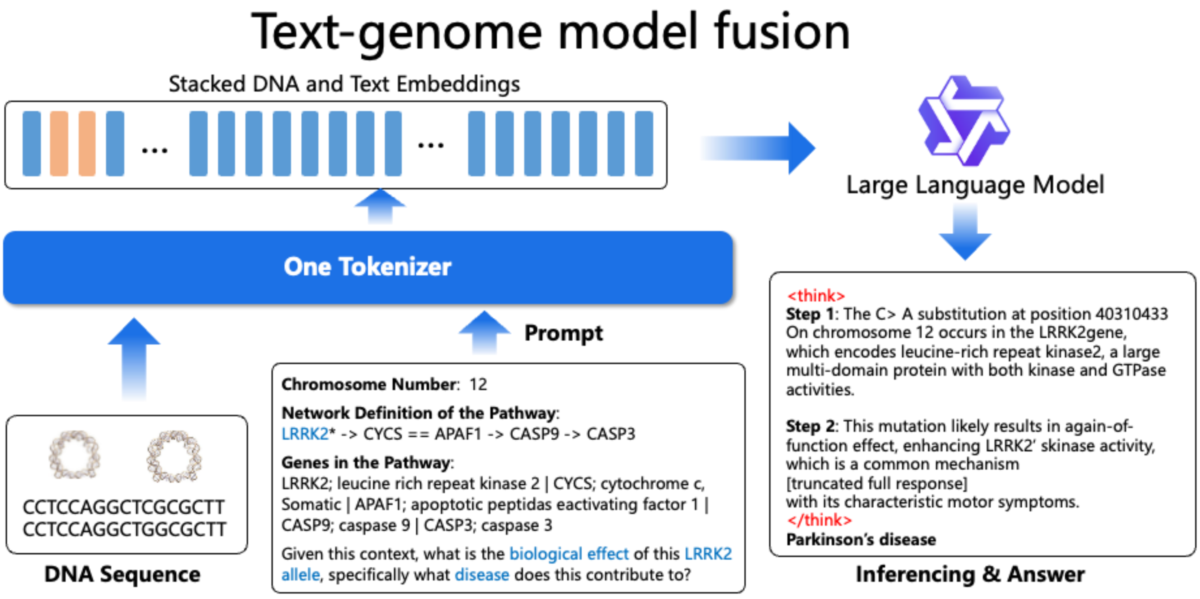

Figure 4 Architecture Design of Genos Model + Language Model

4.2.2 Data Preprocessing and Model Training

In the data preprocessing stage, for DNA sequences, several operations were performed. Firstly, sequence normalization was carried out. All DNA sequences were converted to uppercase letters, and white - space characters were removed. Then, sequence truncation was done using the `truncate_DNA` function, which truncated 1024 base pairs from both ends of the sequence. If the sequence was too short, the middle part was returned. After that, sequence tokenization was performed using character - level taggers. Special tags `<| dna_start |>` `<| dna_pad |>` `<| dna_end |>` were added, and the maximum sequence length was limited to 2048 tokens.

For text preprocessing, the conversation format was converted. The input was transformed into a multimodal format that combined DNA sequences and text. The roles were defined as 'user' which contained DNA sequences and questions, and 'assistant' which contained the reasoning process and

answers. A custom chat template was applied for formatting the input, and special tags <| im_start |> and <| im_end |> were correctly handled.

The dataset was segmented into a training set (80%), a validation set (10%), and a test set (10%). A batch function qwen_DNA_collate_FN was designed specifically for Qwen DNA Models. During the training process, the loss was calculated only for the helper reply part using a label mask. A left - fill policy was adopted, and special markers were added during the fill processing.

4.2.3 Evaluation Indicator Scheme and Results

The evaluation indicator scheme included classification indicators. The basic indicators were Accuracy, which was the proportion of correctly predicted samples to the total sample; Precision, calculated as the macro - average precision (the average of accuracy rates for all categories); Recall rate, which was the macro - average recall rate (the average of all categories of recall rate); and F1 score, the macro - average F1 score (the reconciliation average of precision and recall).

The calculation methods utilized sklearn's classification_report. The report_dict was obtained by calling classification_report with parameters y_true, y_pred, labels, output_dict = True, and zero_division = 1. Then, the macro - average metrics were extracted from the report_dict, and the accuracy was calculated using accuracy_score.

During the validation phase, text was generated in real - time on the validation set, and the answer part was extracted from the generated text. The category indicators were calculated and recorded in the log. In the test phase, a complete evaluation was carried out on the test set. The prediction results were saved as a CSV file, and a detailed classification report was generated.

The results showed that for different models and combinations, the performance varied. For example, in the DNA - only models, the our_gene model achieved an accuracy of 91.72% and an F1-score of 78.75% when evaluated from the code. In the DNA - LLM combined models, the Genos + Qwen4B model achieved an accuracy of 99.31% and an F1 - score of 92.93% when evaluated from the code (**Table 4**), demonstrating the effectiveness of the Omics + Text Interactive Disease Diagnosis model in handling gene variation effect prediction and disease association analysis tasks.

Table 4 Evaluation Indicators of the Genos Model + Text Model Diagnostic Model

Model_type	Model	Dataset	Accuracy	F1-score	Precision	Recall
DNA-only	NT	KEGG	86.55%	69.76%	73.23%	66.62%
	Genos-1B-8K	KEGG	91.72%	78.75%	81.09%	81.64%
LLM-only	Qwen4B	KEGG	93.48%	85.44%	88.31%	86.72%
		KEGG_hard	29.86%	15.15%	67.04%	12.89%
DNA+LLM	NT+Qwen4B	KEGG	96.90%	89.03%	09.00%	89.38%
		KEGG_hard	46.90%	25.50%	78.52%	23.41%
	Genos-1B-8K+Qwen4B	KEGG	99.31%	92.93%	98.48%	93.94%

5. Deployment and Application Prospects

5.1 Current Deployment Status and Usage

Currently, Genos is in the R&D and optimization phase. It is mainly supporting internal scientific research, providing a powerful tool for researchers within the organization to conduct in - depth genomic studies. Genos is designed to be highly adaptable to mainstream GPU environments, with no special hardware restrictions. This compatibility ensures that it can be easily integrated into existing research setups, reducing the barriers to its utilization.

Adhering to the concept of open science, Genos has deployed cloud reasoning services on the Huada DCSCloud platform, thereby constructing a “cloud lab” for genomic intelligence analysis. This open - ecology initiative has far - reaching implications.

Researchers can upload their data through an intuitive interface. Once the data is uploaded, Genos can perform a full - process analysis, starting from mutation function annotation. Mutation function annotation helps in understanding the biological significance of genetic mutations, whether they are benign, pathogenic, or have some other functional implications. The analysis then extends to phenotype prediction, which is a crucial step in connecting genetic information to observable traits. This decentralized computing power support model breaks the shackles of local computing power and algorithm deployment limitations. Researchers from all over the world can now share the predictive power of this leading - edge model. For example, a research team in a resource - limited region can access Genos through the cloud service, enabling them to conduct high - level genomic analysis that was previously out of reach due to lack of local computational resources. This accelerates the transition from genomic discovery to clinical applications, as more research can be carried out and validated, bringing genomic insights closer to patient care.

5.2 Future Application Potential in Biomedicine

In the field of precision medicine, Genos holds great promise. It can analyze an individual's genomic data to identify disease - related genetic markers with high precision. For example, in cancer diagnosis, Genos can analyze tumor - associated genomic variations, predict the aggressiveness of the cancer, and suggest personalized treatment plans. By accurately predicting the response of different patients to various drugs based on their genetic makeup, Genos can help doctors select the most effective treatment options, minimizing the risk of adverse reactions and improving treatment outcomes.

For group health monitoring, Genos can analyze the genomic data of a large population. It can identify genetic factors associated with common diseases in the population, such as cardiovascular diseases, diabetes, and neurodegenerative disorders. This information can be used to develop preventive strategies, such as targeted health education, lifestyle interventions, and early - detection screening programs for high-risk individuals.

In developmental biology, Genos can contribute to understanding the genetic basis of embryo development. By analyzing the genomic changes during different stages of embryo development, it can uncover the regulatory mechanisms that control cell differentiation, organ formation, and overall

development. This knowledge can help in diagnosing and treating developmental disorders and also provide insights into reproductive medicine, such as improving in vitro fertilization techniques. As Genos continues to optimize and iterate, its application potential in these biomedical fields will continue to expand, laying a solid foundation for the development of a more comprehensive and effective healthcare system.

6. Conclusion

6.1 Summary of Research Findings

Genos represents a significant advancement in genomic intelligence analysis. Specifically, its Mixture-of-Experts (MoE) architecture effectively addresses the computational challenges inherent in ultra-long sequence modeling at single-nucleotide resolution. By introducing strategies such as ultra - long sequence parameterization, multi - dimensional parallel computing, and complementary attention mechanisms, Genos successfully overcomes the limitations of traditional models in handling million - base sequences. The expert load balancing mechanism, mixed-precision training strategy and dynamic routing architecture further enhance the model's training stability and inference efficiency.

In terms of performance, Genos outperforms existing models in various benchmark tasks. In addition to the existing benchmark comparisons, we designed two specific tasks focused on ultra-long sequence modeling. Across these tasks, the Genos model exhibited a clear positive correlation between sequence length and prediction accuracy. In contrast, other models were either unable to process sequences of such lengths or did not demonstrate this property of performance scaling with increased sequence context. This finding thus provides empirical evidence for the necessity of longer context windows.

The application cases of Genos further validate its utility. In RNA - seq data generation, Genos can accurately predict gene expression levels, as demonstrated by high Pearson correlation coefficients between predicted and true expression values. In the omics + text interactive disease diagnosis project, Genos, when combined with a large - scale language model, achieves high accuracy in gene variation effect prediction and disease association analysis, with accuracy rates reaching up to 99.31% in some cases.

6.2 Limitations and Future Work

The Genos model has several limitations that must be addressed in future work. Firstly, computational efficiency requires optimization. Although the architecture is designed for effective resource allocation, there is still potential to significantly reduce the computational cost during training and inference, particularly when handling massive datasets. Secondly, the capability for cross-modal data fusion needs enhancement. While Genos shows initial promise with genomic data, deeper integration of multi-omics data, such as proteomics and metabolomics, alongside phenotypic information, is essential to achieve a more comprehensive understanding of complex biological processes and gene-environment interactions.

Future model development will involve continuous training with an increasingly diverse set of genomic data, with the primary objective remaining a deeper comprehension of the human genome and superior performance in corresponding analytical applications. Furthermore, the integration of

other multi-omics datasets with the Genos genomic model is anticipated to offer substantial benefits for downstream research and practical applications.

.

6.3 Significance of Genos for Genomics Development

Genos is expected to have a substantial impact on the trajectory of genomics research. It marks a paradigm shift from traditional data - driven genomics research to an fundation model - based approach. By providing a powerful tool for accurate and efficient genomic analysis, Genos enables researchers to gain deeper insights into the genetic basis of diseases.

Within the domain of precision medicine, Genos may play a crucial role in disease risk prediction, personalized diagnosis, and treatmen stratificationt. Its capacity to analyze genomic data at a high level of accuracy can aid in identifying disease-associated genetic variants, predicting the efficacy of drugs, and developing personalized treatment plans. This may lead to more effective and targeted medical interventions, reducing the cost and side - effects associated with traditional treatment methods.

Moreover, Genos contributes to a more comprehensive understanding of life processes. By decoding the intricate genomic information, it paves the way for advancements in fields such as developmental biology, evolutionary biology, and synthetic biology. Overall, Genos is a key step towards realizing the full potential of genomics in improving human health and understanding the mysteries of life.

Data and Code Availability

To facilitate reproducible research and community collaboration, all resources for the Genos model are publicly accessible. Pre-trained model weights, inference code, and detailed documentation are released on GitHub (https://github.com/BGI-HangzhouAI/Genos) and the Hugging Face Hub (https://huggingface.co/BGI-HangzhouAI/Genos). These resources enable researchers to fine-tune Genos for specialized genomic tasks or integrate it into custom bioinformatic workflows. The model is distributed under the MIT License, permitting unrestricted use, modification, and redistribution for both academic and commercial purposes. For users seeking scalable cloud-based inference, Genos is also deployed on the BGI DCS Cloud platform, with dedicated APIs to support end-to-end genomic analysis without local computing infrastructure.

Disclosure of use of AI-assisted tools including generative AI

In the preparation of this manuscript, an AI-assisted tool (Doubao) was used to support the optimization of academic writing structure (e.g., organizing the logical flow of the Methodology section and Abstract), and refine the expression of technical content. All content generated or optimized with the assistance of this tool was thoroughly reviewed, verified, and revised by the authors to ensure accuracy, academic rigor, and consistency with the study's original findings

Acknowledgement

We would like to acknowledge The Human Genome Structural Variation Consortium (HGSVC) and the Human Pangenome Reference Consortium (BioProject ID: PRJNA698480) , as well as their funder, the National Human Genome Research Institute (NHGRI).

The model training process was conducted entirely on the 021 Large Science Model and Zero2X open platform.

References

Ainslie, J., Lee-Thorp, J., Jong, M., Zemlyanskiy, Y., Lebrón, F., and Sanghai, S. (2023). GQA: Training Generalized Multi-Query Transformer Models from Multi-He ad Checkpoints.

Avsec, Ž., Latysheva, N., Cheng, J., Novati, G., Taylor, K.R., Ward, T., Bycroft, C., Nicolaisen, L., Arvaniti, E., Pan, J., et al. (2025). AlphaGenome: advancing regulatory variant effect prediction with a unified DNA sequence model. bioRxiv, 2025.2006.2025.661532. 10.1101/2025.06.25.661532.

Consortium, E.P. (2012). An integrated encyclopedia of DNA elements in the human genome. 2010-04-01 *489*, 57-74. 10.1038/nature11247.

Dalla-Torre, H., Gonzalez, L., Mendoza-Revilla, J., Lopez Carranza, N., Grzywaczewski, A.H., Oteri, F., Dallago, C., Trop, E., de Almeida, B.P., Sirelkhatim, H., et al. (2025). Nucleotide Transformer: building and evaluating robust foundation models for human genomics. Nat Methods *22*, 287-297. 10.1038/s41592-024-02523-z.

Dao, T., Fu, D.Y., Ermon, S., Rudra, A., and Ré, C. (2022). FlashAttention: Fast and Memory-Efficient Exact Attention with IO-Awar eness.

Fairley, S., Lowy-Gallego, E., Perry, E., and Flicek, P. (2020). The International Genome Sample Resource (IGSR) collection of open human genomic variation resources. Nucleic Acids Res *48*, D941-D947. 10.1093/nar/gkz836.

Fallahpour, A., Magnuson, A., Gupta, P., Ma, S., Naimer, J., Shah, A., Duan, H., Ibrahim, O., Goodarzi, H., Maddison, C.J., and Wang, B. (2025). BioReason: Incentivizing Multimodal Biological Reasoning within a DNA- LLM Model.

Gao, Y., Yang, X., Chen, H., Tan, X., Yang, Z., Deng, L., Wang, B., Kong, S., Li, S., Cui, Y., et al. (2023). A pangenome reference of 36 Chinese populations. 2010-04-01 *619*, 112-121. 10.1038/s41586-023-06173-7.

Hickey, G., Monlong, J., Ebler, J., Novak, A.M., Eizenga, J.M., Gao, Y., Human Pangenome Reference, C., Marschall, T., Li, H., and Paten, B. (2024). Pangenome graph construction from genome alignments with Minigraph-Cactus. Nat Biotechnol *42*, 663-673. 10.1038/s41587-023-01793-w.

Hwang, C., Cui, W., Xiong, Y., Yang, Z., Liu, Z., Hu, H., Wang, Z., Salas, R., Jose, J., Ram, P., et al. (2022). Tutel: Adaptive Mixture-of-Experts at Scale.

Jacobs, R.A., Jordan, M.I., Nowlan, S.J., and Hinton, G.E. (1991). Adaptive Mixtures of Local Experts. Neural Computation, 79-87. 10.1162/neco.1991.3.1.79.

Kim-Hellmuth, S., Aguet, F., Oliva, M., Munoz-Aguirre, M., Kasela, S., Wucher, V., Castel, S.E., Hamel, A.R., Vinuela, A., Roberts, A.L., et al. (2020). Cell type-specific genetic regulation of gene expression across human tissues. Science *369*. 10.1126/science.aaz8528.

Liao, W.W., Asri, M., Ebler, J., Doerr, D., Haukness, M., Hickey, G., Lu, S., Lucas, J.K., Monlong, J., Abel, H.J., et al. (2023). A draft human pangenome reference. 2010-04-01 *617*, 312-324. 10.1038/s41586-023-05896-x.

Liu, J., Tang, P., Wang, W., Ren, Y., Hou, X., Heng, P.-A., Guo, M., and Li, C. (2024). A Survey on Inference Optimization Techniques for Mixture of Experts M odels.

Logsdon, G.A., Ebert, P., Audano, P.A., Loftus, M., Porubsky, D., Ebler, J., Yilmaz, F., Hallast, P., Prodanov, T., Yoo, D., et al. (2025). Complex genetic variation in nearly complete human genomes. 2010-04-01 *644*, 430-441. 10.1038/s41586-025-09140-6.

Loshchilov, I., and Hutter, F. (2017). Decoupled Weight Decay Regularization.

Nguyen, E., Poli, M., Faizi, M., Thomas, A., Birch-Sykes, C., Wornow, M., Patel, A., Rabideau, C., Massaroli, S., Bengio, Y., et al. (2023). HyenaDNA: Long-Range Genomic Sequence Modeling at Single Nucleotide Re solution.

Radford, A., and Narasimhan, K. (2018). Improving Language Understanding by Generative Pre-Training.

Shazeer, N., Mirhoseini, A., Maziarz, K., Davis, A., Le, Q., Hinton, G., and Dean, J. (2017). Outrageously Large Neural Networks: The Sparsely-Gated Mixture-of-Expe rts Layer.

Shoeybi, M., Patwary, M., Puri, R., LeGresley, P., Casper, J., and Catanzaro, B. (2019). Megatron-LM: Training Multi-Billion Parameter Language Models Using Mo del Parallelism.
Su, J., Ahmed, M., Lu, Y., Pan, S., Bo, W., and Liu, Y. (2024). RoFormer: Enhanced transformer with Rotary Position Embedding. Neurocomputing *568*, 127063. https://doi.org/10.1016/j.neucom.2023.127063.
Trop, E., Schiff, Y., Marroquin, E.M., Kao, C.H., Gokaslan, A., Polen, M., Shao, M., Kallala, A., Almeida, B.P., Pierrot, T., et al. (2025). The Genomics Long-Range Benchmark: Advancing DNA Language Models.
Vaswani, A., Shazeer, N., Parmar, N., Uszkoreit, J., Jones, L., Gomez, A.N., Kaiser, L., and Polosukhin, I. (2017). Attention Is All You Need.
Vollger, M.R., Dishuck, P.C., Harvey, W.T., DeWitt, W.S., Guitart, X., Goldberg, M.E., Rozanski, A.N., Lucas, J., Asri, M., Human Pangenome Reference, C., et al. (2023). Increased mutation and gene conversion within human segmental duplications. 2010-04-01 *617*, 325-334. 10.1038/s41586-023-05895-y.
Wang, X., Tissue, H., Wang, L., Li, L., and Zeng, D.D. (2025). Learning Dynamics in Continual Pre-Training for Large Language Models.
Wu, W., Li, Q., Li, M., Fu, K., Feng, F., Ye, J., Xiong, H., and Wang, Z. (2025). GENERator: A Long-Context Generative Genomic Foundation Model.
Zhai, X., Kolesnikov, A., Houlsby, N., and Beyer, L. (2021). Scaling Vision Transformers. arXiv e-prints, arXiv:2106.04560, arXiv:2106.04560. 10.48550/arXiv.2106.04560.
Zhang, B., and Sennrich, R. (2019). Root Mean Square Layer Normalization. arXiv e-prints, arXiv:1910.07467, arXiv:1910.07467. 10.48550/arXiv.1910.07467.
Zoph, B., Bello, I., Kumar, S., Du, N., Huang, Y., Dean, J., Shazeer, N., and Fedus, W. (2022). ST-MoE: Designing Stable and Transferable Sparse Expert Models.
