# Supplementary material for: Genos: a human-centric genomic foundation model
Source: Gigascience. 2026 Jan 1;14:giaf132. doi: 10.1093/gigascience/giaf132 (PMC12755919; doi:10.1093/gigascience/giaf132)
Supplement: giaf132_GIGA-D-25-00428_Revision_2 [file giaf132_giga-d-25-00428_revision_2.pdf]

GigaScience

Genos: A Human-Centric Genomic Foundation Model

--Manuscript Draft--

**Manuscript Number:**	GIGA-D-25-00428R2
**Full Title:**	Genos: A Human-Centric Genomic Foundation Model
**Article Type:**	Research
**Funding Information:**	
**Abstract:**	The rapid expansion of human genomic data demands foundation models that manage ultra-long sequences and capture population diversity, limitations common in existing models which lack human-specific representation and clinical inference efficiency. Here, we introduce Genos (Genos-1.2B/Genos-10B), a human-centric genomic foundation model engineered for million-base-pair sequence modeling. Genos utilizes a large-scale Mixture of Experts (MoE) structure, optimized for a 1Mb context, trained on high-quality human de novo assemblies from datasets such as HPRC and HGSVC, representing diverse global populations. A suite of optimization strategies was implemented to ensure training stability and enhance computational efficiency, which collectively reduces costs and facilitates million-base-pair context modeling. Functionally, Genos performs single-nucleotide resolution analysis and dynamically simulates the cascade effects of non-coding variations on RNA expression profiles. In comprehensive evaluations, Genos uniformly surpasses State-of-the-Art models on critical human genomics benchmarks and demonstrates robust Omics-Text cross-modal diagnostic capabilities. We present a systematic technical evaluation and validation of Genos's architecture, training convergence, and performance across standard benchmarks. This work provides a reliable technical blueprint and performance benchmark for the development of the next generation of high-efficiency genomic foundation models. Genos model weights, inference code, and usage documentation are publicly available on GitHub (https://github.com/BGI-HangzhouAI/Genos) and Hugging Face Hub (https://huggingface.co/BGI-HangzhouAI), with additional cloud services accessible via BGI DCS Cloud—all released under the MIT License.
**Corresponding Author:**	Duoyuan Chen<br>Genos Team<br>HANGZHOU, CHINA
**Corresponding Author Secondary Information:**	
**Corresponding Author's Institution:**	Genos Team
**Corresponding Author's Secondary Institution:**	
**First Author:**	Adi Lin
**First Author Secondary Information:**	
**Order of Authors:**	Adi Lin
	Bin Xie
	Cheng Ye
	Cheng Wang
	Duoyuan Chen
	Ercheng Wang
	Fanfeng Lu
	Guirong Xue
	Haiqiang Zhang

Powered by Editorial Manager® and ProduXion Manager® from Aries Systems Corporation

	Jiajie Zhan
	Jianfeng Zhang
	Jiangshuan Pang
	Jianqiang Liang
	Jiawei Lin
	Jiaxin Ma
	Jie Hu
	Jing Ma
	Jinni Dong
	Jiongzhen Li
	Junchen Liu
	Junhong Chen
	Junyou Li
	Kai Ding
	Kaiwen Deng
	Kui Chen
	Lihui Wang
	Longqi Liu
	Ling Guo
	Liwen Xiong
	Luhao Yang
	Ming Cheng
	Nanning Chen
	Renzhong Chen
	Shanxin Sun
	Shaoshuai Li
	Shicheng Chen
	Shiping Liu
	Siwei Xie
	Suyan Liu
	Tao Zhou
	Wangyang Tang
	Weiqiang Zhang
	Xianyue Jiang
	Xianzhi Qi
	Xin Jin
	Xinjiang Tan
	Xinyue Hu
	Xun Xu
	Xuyang Feng
	Yafei Lu

Powered by Editorial Manager® and ProduXion Manager® from Aries Systems Corporation

	Yifan Gao
	Yong Shang
	Youzhe He
	Yue Yuan
	Yufan Wang
	Yuqi Liu
	Zhan Xiao
	Zhangyuan Meng
	Zhaorong Li
	Zhe Zhao
	Zheng Yang
	Zilin Wang
**Order of Authors Secondary Information:**	
**Response to Reviewers:**	All issues raised by the reviewers have been revised and updated.
**Additional Information:**	
**Question**	**Response**
Are you submitting this manuscript to a special series or article collection?	No
**Experimental design and statistics**<br><br>Full details of the experimental design and statistical methods used should be given in the Methods section, as detailed in our Minimum Standards Reporting Checklist. Information essential to interpreting the data presented should be made available in the figure legends.<br><br>Have you included all the information requested in your manuscript?	Yes
**Resources**<br><br>A description of all resources used, including antibodies, cell lines, animals and software tools, with enough information to allow them to be uniquely identified, should be included in the Methods section. Authors are strongly encouraged to cite Research Resource Identifiers (RRIDs) for antibodies, model organisms and tools, where possible.	Yes

Have you included the information requested as detailed in our Minimum Standards Reporting Checklist?	
**Availability of data and materials**<br><br>All datasets and code on which the conclusions of the paper rely must be either included in your submission or deposited in publicly available repositories (where available and ethically appropriate), referencing such data using a unique identifier in the references and in the “Availability of Data and Materials” section of your manuscript.<br><br>Have you have met the above requirement as detailed in our Minimum Standards Reporting Checklist?	Yes
GigaScience has policies and guidelines in place for the use of generative AI-writing tools such as ChatGPT. If you have used such writing tools to assist with writing the manuscript this must be declared and cited in the text. Authors should not list AI-writing tools and other AI-assisted technologies as an author or co-author and should acknowledge that they are fully responsible for text generated or refined by AI-writing tools.<p><br><br>A summary of use (particularly in the introduction or among methods) needs to be included at the end of the paper, and the outputs should also be included as a supplementary file hosted in GigaDB or other open repositories. Please read our guidelines for more information. <p><br><br>By submitting to GigaScience, you are aware of the journal's AI-writing tools policy, and if you have declared use of such tools below, you have acknowledged this where appropriate in your manuscript and have made a summary of use and outputs available. <p><br><b>AI-assisted writing tools have been used in the preparation of this manuscript?	Yes

Manuscript

Click here to access/download;Manuscript;[10.21]Genos A Human Centric Genomic Foundation Model.docx

Genos: A Human-Centric Genomic Foundation Model

Adi Lin[1], Bin Xie[1], Cheng Ye[1],Cheng Wang[1], Duoyuan Chen[1], Ercheng Wang[1], Fanfeng Lu[1], Guirong Xue[1], Haiqiang Zhang[1], Jiajie Zhan[1], Jianfeng Zhang[1], Jiangshuan Pang[1], Jianqiang Liang[1], Jiawei Lin[1], Jiaxin Ma[1], Jie Hu[1], Jing Ma[1],Jinni Dong[1], Jiongzhen Li, Junchen Liu[1], Junhong Chen[1], Junyou Li[1], Kai Ding[1], Kaiwen Deng[1], Kui Chen[1], Lihui Wang[1], Longqi Liu[1], Ling Guo[1], Liwen Xiong[1], Luhao Yang[1], Ming Cheng[1], Nanning Chen[1], Renzhong Chen[1], Shanxin Sun[1], Shaoshuai Li[1], Shicheng Chen[1], Shiping Liu[1], Siwei Xie[1], Suyan Liu[1], Tao Zhou[1], Wangyang Tang[1], Weiqiang Zhang[1], Xianyue Jiang[1], Xianzhi Qi[1], Xin Jin[1], Xinjiang Tan[1], Xinyue Hu[1], Xun Xu[1], Xuyang Feng[1], Yafei Lu[1] ,Yifan Gao[1], Yong Shang[1], Youzhe He[1], Yue Yuan[1], Yufan Wang[1], Yuqi Liu[1], Zhan Xiao[1], Zhangyuan Meng[1], Zhaorong Li[1], Zhe Zhao[1], Zheng Yang[1], Zilin Wang[1]

[1] Genos team, Hangzhou, China

[**]All authors contributed equally, Authors are ranked in alphabetical order by their first names.

Corresponding Author: Xun Xu. E-Mail: xuxun@genomics.cn,

ORCID: [0000-0002-5338-5173].

Abstract

The rapid expansion of human genomic data demands foundation models that manage ultra-long sequences and capture population diversity, limitations common in existing models which lack human-specific representation and clinical inference efficiency. Here, we introduce Genos (Genos-1.2B/Genos-10B), a human-centric genomic foundation model engineered for million-base-pair sequence modeling. Genos utilizes a large-scale Mixture of Experts (MoE) structure, optimized for a 1Mb context, trained on high-quality human *de novo* assemblies from datasets such as HPRC and HGSVC, representing diverse global populations. A suite of optimization strategies was implemented to ensure training stability and enhance computational efficiency, which collectively reduces costs and facilitates million-base-pair context modeling. Functionally, Genos performs single-nucleotide resolution analysis and dynamically simulates the cascade effects of non-coding variations on RNA expression profiles. In comprehensive evaluations, Genos uniformly surpasses State-of-the-Art models on critical human genomics benchmarks and demonstrates robust Omics-Text cross-modal diagnostic capabilities. We present a systematic technical evaluation and validation of Genos's architecture, training convergence, and performance across standard benchmarks. This work provides a reliable technical blueprint and performance benchmark for the development of the next generation of high-efficiency genomic foundation models. Genos model weights, inference code, and usage documentation are publicly available on GitHub (https://github.com/BGI-HangzhouAI/Genos) and Hugging Face Hub (https://huggingface.co/BGI-HangzhouAI), with additional cloud services accessible via BGI DCS Cloud—all released under the MIT License.

1. Introduction

1.1 The Paradigm Shift: Genomics and Foundation Models

Genomics research is currently transitioning from an early phase of massive data accumulation to the contemporary era of intelligent analysis and insight extraction. The proliferation of high-throughput sequencing technologies has generated an unprecedented volume of nucleic acid sequence data, making deep learning-based Genomic Foundation Models (GFMs) a crucial computational tool for deciphering the complexity of life. Analogous to Large Language Models (LLMs) in Natural Language Processing, GFMs aim to learn the intrinsic "grammar" and "semantics" of the genome through large-scale pre-training, enabling unified analysis of functional element identification, variant pathogenicity prediction, and phenotype regulatory networks. This technological breakthrough is pivotal for accelerating precision medicine and population health research.

1.2 Significance of Genos in the Field

Significant progress has been made in the GFM landscape, with seminal works like EVO2 [1] and AlphaGenome [2] leading the trend toward long-sequence modeling and cross-species generalization. However, when these models are applied to human translational medicine and clinical high-throughput analysis, they encounter two core bottlenecks.

Bottleneck I: The Human-Centric Representational Gap. The OpenGenome2 dataset used by EVO2 prioritizes cross-species coverage over population diversity, leading to systematic bias in the representation of human-specific regulatory elements (e.g., enhancers, promoters) and rare variants. Similarly, AlphaGenome relies on cohorts with limited reference genomes, struggling to accurately capture complex population-specific genetic patterns. This fundamentally restricts the models' predictive accuracy and generalizability in complex human disease and rare disorder research.

Bottleneck II: Efficiency and Deployment Challenges for Ultra-Long Sequences. While existing models have achieved context modeling up to the million-base-pair (1Mb) scale, this often incurs prohibitive computational costs. For instance, the 40B-parameter version of EVO2 requires extensive GPU clusters for training and exhibits high inference latency, unsuitable for time-sensitive clinical analysis. Furthermore, specialized architectures often lack modularity, making them incompatible with mainstream cloud computing infrastructures, significantly raising the barrier to deployment and broad application.

Considering the two core bottlenecks, we focused our efforts on robust data engineering and an optimized technical architecture. On the data side, we curated a human-centric, multi-source dataset, integrating high-quality, haplotype-resolved assemblies from the Human Pangenome Reference Consortium (HPRC) [3-5] and the Human Genome Structural Variation Consortium (HGSVC) [6] to ensure robust cross-ethnic generalizability. Architecturally, we rooted our design in an evolved Transformer framework, augmenting it with a Mixture-of-Experts (MoE) [7] structure to address the computational challenges of modeling sequences up to a million bases. This was achieved by integrating elements such as Rotary Position Embedding (RoPE) [8] for extreme context lengths, and multiple parallelism strategy (including Tensor, Pipeline, Context, Data, and Expert Parallelism) to ensure stable and efficient large-scale training. This comprehensive work on data and architecture, coupled with extensive training and optimization, culminated in the release of Genos (Genos-1.2B/Genos-10B), a human-centric Genomic Foundation Model.

Genos stands at the forefront of genomic foundation models, playing a pivotal role in the field of genomics. It has the potential to revolutionize multiple aspects of genomic research and its applications. In precision medicine, Genos can analyze an individual's genomic data to predict disease risks with greater accuracy. For instance, by identifying key genetic markers associated with diseases such as cancer or neurodegenerative disorders, facilitates the development of personalized treatment regimens. This not only improves the effectiveness of treatment but also reduces the risk of adverse reactions to medications.

In the realm of group health monitoring, by analyzing genomic data from large populations, the model facilitates the precise identification genetic trends within different ethnic groups, which is crucial for understanding the genetic basis of diseases prevalent in specific populations. These critical genomic insights provide the scientific foundation necessary for formulating can be used to develop targeted preventive measures and healthcare policies. In developmental biology, Genos can help in understanding the genetic mechanisms underlying embryo development. By analyzing the genomic sequences at different stages of development, researchers can uncover how genes are regulated to drive the formation of various tissues and organs.

1.3 Objectives and Core Design Feature of Genos

The objective for Genos is to provide a genomic intelligence analysis engine characterized by superior accuracy and efficiency, thereby advancing the field into a mass application phase. Genos provides significant methodological advancements.

In data processing, Genos integrates standardized, high-quality data from leading international genomics initiatives, including the Human Pangenome Reference Consortium (HPRC) [3-5] and the Human Genome Structural Variation Consortium (HGSVC) [6]. By constructing a multi-source, heterogeneous genomic dataset spanning global populations and incorporating hundreds of nearly telomere-to-telomere (T2T) assemblies, Genos achieves robust cross-ethnic generalizability.To ensure the reliability and representativeness of training data, we designed a multi-stage quality control pipeline that progressively filters out intergenic sequences of varying lengths, many of which contain segmental duplication (SD) regions.

The model's architecture is rooted in an evolved Transformer [9] framework, augmented by a Mixture-of-Experts (MoE) [7] structure. This design effectively overcomes the long-standing computational challenge associated with modeling sequences that exceed a million bases. The integration of ultra-long sequence parameterization, multi-dimensional parallel computing, and specialized complementary attention mechanisms allows Genos to perform single-nucleotide resolution modeling on ultra-long sequences. Consequently, this provides a more comprehensive analytical depth, allowing for the precise capture and analysis of fine-scale genetic details across the entire genome.

Functionally, Genos has the core ability to accurately identify key functional elements in the genome. It can deeply analyze the cascade effect of micro-gene variation on the transcriptional regulatory network. This is a significant improvement over traditional methods, which often have limitations in predicting regulatory elements in the non-coding region. Genos is capable of single-nucleotide resolution analysis within ultra-long non-coding regions and can dynamically simulate the cascade

effect of variation sites on RNA expression profiles, offering a novel paradigm for molecular mechanism analysis.

2. Methodology

2.1 Data Collection and Preprocessing

The training data for Genos were curated from multiple high-quality genomic sources, including 231 haplotype-resolved assemblies from the HPRC (release 2), 65 assemblies from the HGSVC, and 21 genomes from the Centre d'Etude du Polymorphisme Humain (CEPH) cohort, along with two reference genomes, GRCh38 and CHM13. In total, the dataset comprises 636 high-quality genomes, representing diverse global populations. Each genome sequence was processed using a one-hot tokenizer, with a vocabulary consisting of the four canonical nucleotides (A, T, C, G), the undetermined base N, and special tokens such as <EOD> marking sequence boundaries. No cell-type-specific labels, epigenetic features (e.g., histone modifications), or other functional annotations were incorporated during this stage. This ensures that the model learns a general-purpose representation of the human genome, unbiased towards any particular biological context or experimental condition.

Training was performed in two major stages. In the pre-training stage, samples from HPRC release 2 were divided into four groups at an approximate 3:3:3:1 ratio, corresponding to sequence lengths of 8,192 bp, 32,768 bp, 131,072 bp, and 1,024,000 bp. Within each stage, about one-quarter of the samples had both haplotypes reverse-complemented, while the remaining samples retained the forward strand orientation. Samples from HGSVC and CEPH pedigrees were all processed into 8,192 bp fragments, with one-quarter of them reverse-complemented in the same manner. Both reference genomes (GRCh38 and CHM13) were prepared with both forward and reverse strands at every length scale. To reduce non-informative intergenic content, 8,192 bp fragments excluded regions located more than 5,120 bp away from any gene boundary, while 32,768 bp fragments excluded regions beyond 10,240 bp from gene boundaries. The four pre-training datasets were then sequentially introduced to the model by increasing sequence length, resulting in a total of approximately 1.4 trillion (1,400B) tokens. In the subsequent continued pre-training (CPT) stage, the same samples were reshuffled across lengths and strand orientations to generate an additional 2.6 trillion (2,600B) tokens, which were further randomized before being fed into the model.

Crucially, it is important to emphasize that all filtering was discontinued in the subsequent Continued Pre-Training (CPT) stage. The additional 2.6 trillion tokens used in CPT were generated from the original samples without any intergenic distance-based exclusion. This ensured that the model was extensively exposed to and trained on distal intergenic regions, segmental duplications, transposable elements, and other complex genomic architectures, thereby cultivating a comprehensive understanding of the entire genomic landscape, including essential ‘negative’ background sequences.

2.2 Model Architecture Design

Genos employs a MoE architecture evolved from the Transformer, characterized by 12 layers, optimized for both performance and efficiency in genomic sequence modeling. The Mixture-of-Experts (MoE) architecture is established to provide intrinsic performance benefits beyond mere computational efficiency, a principle supporting our genomic model's design. Foundational work demonstrated that MoE models achieve superior perplexity and accuracy over

dense baselines under identical computational budgets [10]. This enhanced modeling capability, attributed to expert specialization and conditional computation leveraging a vast parameter space, is consistently validated across large-scale language models [11] and complex data domains [12]. Consequently, we directly adopted this well-established architectural design, and the resulting performance gains observed in our Genos model align with theoretical expectations and the established body of evidence.

The model begins with a token embedding layer that converts discrete base tokens into continuous vector representations. Following embedding, three root mean square normalization (RMSNorm) [13] layers are strategically placed throughout the network to stabilize training by re-scaling inputs to have a root mean square of one, without re-centering them around the mean. Between the first and second RMSNorm layers, Genos integrates Rotary Position Embedding (RoPE) [8] with an exceptionally large base frequency of 50,000,000, enabling it to process ultra-long sequences of up to 1 million tokens. Notably, instead of using explicit position embeddings at the input layer, RoPE dynamically injects positional information during attention computation by applying rotary transformations to query and key vectors. This design offers precise positional awareness while supporting extreme context lengths. Complementing RoPE, the model employs a Grouped-Query Attention (GQA) [14] mechanism with 16 attention heads sharing 8 key-value groups. This configuration strikes an optimal balance between computational efficiency and representational capacity, allowing Genos to process long genomic sequences both accurately and efficiently. Genos adopted MoE architecture, which consists of a router network and eight expert subnetworks. Each expert subnetwork utilizes SwiGLU [15] activation functions, replacing traditional ReLU/GELU for improved expressive capability and training stability. The router dynamically selects two out of the eight experts for each token based on sequence content, allocating computational resources adaptively (**Figure 1**). This design enables efficient processing of both simple repetitive regions and complex regulatory elements. Finally, a linear output layer projects the model's final hidden state into logits over the vocabulary, where the softmax function then converts them into a probability distribution for the next token, in accordance with the Next Token Prediction (NTP) objective [16]. A key advantage of this model architecture is its inherent flexibility, which enables effective adaptation to various downstream applications.

2.3 Pre-training Process and Parameter Optimization

During the pre-training phase, Genos was trained through the self-supervised paradigm. The model employs the NTP objective while producing general genomic representations.

The model was trained using the Megatron-LM framework [17] across 256 GPUs, employing a sophisticated five-dimensional parallelism strategy that combines Tensor Parallelism, Pipeline Parallelism, Context Parallelism, Data Parallelism, and Expert Parallelism.

Training was conducted with a global batch size of 1,024, achieved via gradient accumulation using a micro-batch size of 1. The optimization process used the AdamW [18] optimizer with a distributed sharded implementation for optimizer states. The learning rate followed a cosine decay schedule, starting with a 5% warm-up phase and peaking at 1e-4, accompanied by gradient clipping set at 1.0 and weight decay of 0.1.

To address the inherent challenge of expert load imbalance in the MoE architecture—particularly pronounced due to the limited vocabulary of genomic sequences (four bases)—we implemented an expert load balancing mechanism with auxiliary loss [10] (coefficient 1e-3). This approach prevents

router collapse and ensures uniform activation of experts across diverse genomic contexts. A Z-loss [19] penalty (coefficient 1e-3) applied to router logits to prevent numerical instability and ensure smoother training in the MoE components.

To achieve ultra-long context modeling (up to 1M tokens), we implemented a multi-stage progressive training strategy. This approach incorporated three key technical components: training on data with progressively increasing context lengths, scheduled learning rate decay to effectively mitigate catastrophic forgetting [20], and the application of RoPE-based context window scaling.

To enhance numerical stability and training quality, mixed-precision training was adopted. This involving utilizing BF16 for the majority computations while stricted retaining FP32 precision for critical operations, specifically (1) the Softmax function within the attention mechanism, (2) gradient accumulation and All-Reduce communications, and (3) MoE routing. Simultaneously, reduced-precision matrix multiplication via BF16 was explicitly disabled.

By integrating GQA and Flash Attention [21], Genos capitalizes on their complementary strengths. GQA provides architectural innovations essential for efficient KV caching, while Flash Attention offers an optimized computational kernel for the rapid calculation of attention scores. This synergy established a robust foundation for a high-performance large-scale pre-training model capacity for extensive context windows.

Additional optimizations included: Grouped GEMM (General Matrix Multiplication) [22] operations for efficient batched expert computation in MoE layers; AllToAll token dispatching [23] for MoE communication; Overlapped parameter gathering and gradient reduction to minimize communication latency [24]; A cyclic data loader with 8 workers to support continuous data streaming during large-scale pretraining.

2.4 Inference and Downstream Applications

During inference, Genos leverages its adaptive routing mechanism and GQA to efficiently process sequences lengths ranging up to 1 Mb. The model supports three primary modalities: embedding generation, sequence generation, and model fine-tuning. For embedding generation, Genos produces fixed-dimensional vector representations that capture biological features for tasks such as sequence clustering and multi-omics integration. In sequence generation mode, the model functions as an autoregressive decoder with sampling strategies including temperature scaling and top-k filtering to simulate novel sequences or mutated alleles. On-demand fine-tuning via adapter modules or continual learning is available through its hugginface service, enabling customization for specialized tasks such as rare disease variant annotation without requiring full model retraining (**Figure 1**). This aims to facilitate deployment across various genomic research and clinical applications.

2.5 Scalable Model Variants: Genos-1.2B and Genos-10B

To address diverse computational constraints and application scenarios, we developed two versions of the Genos model (1.2B and 10B), with architectural details summarized in **Table 1**. Compared to the 1.2B variant, the 10B version exhibits substantial improvement across core configuration: the attention hidden dimension scales from 1,024 to 4,096, enhancing contextual understanding; the MoE hidden dimension per expert quadruples from 4096 to 8192, boosting representational capacity within each expert subnetwork; consequently, the total number of parameters rises from 1.25 billion to 10.27 billion, expanding model capacity; accordingly, the activated parameter count rises from 0.33 billion to 2.87

billion, reflecting the selective utilization inherent in MoE designs. We trained both versions of the model using the same dataset. For this release, the 10B version has been trained on 2,200B tokens, which is slightly higher than the 1,600B tokens used for the 1.2B version. The contrasting scales of the models define their optimal use cases: the 1.2B version is dedicated to resource-constrained analysis, and most fine-tuning scenarios, while the 10B version is geared towards intensive, high-capacity modeling requirements (e.g.,whole-genome structural variation interpretation). A schematic overview of these key capabilities—single-base resolution and ultra-long context modeling—is provided in **Figure 2A**. The model training process was conducted entirely on the 021 Large Science Model and Zero2X open platform.

Table 1 Architectural Details of Two Versions of Genos Model

Version	1.2B	10B
Architecture	MoE	
Number of Total Parameters	1.25B	10.27B
Number of Activated Parameters	0.33B	2.87B
Number of Layers	12	
Attention Hidden Dimension	1024	4096
MoE Hidden Dimension (per Expert)	4096	8192
Number of Attention Heads	16	
Number of Experts	8	
Selected Experts per Token	2	
Vocabulary Size	128(padded)	256(padded)
Context Length	up to 1M	
Attention Mechanism	GQA&Flash Attention	
Activation Function	SWiGLU	
Trained Tokens	1600 B	2200 B

3. Performance Evaluation

3.1 Benchmark Evaluation and Downstream Application Task

We utilized several standard benchmark datasets to evaluate Genos. We firstly assessed across a suite of established genomics benchmarks, including the Genomics Benchmark (GB), Nucleotide Transformer Benchmark (NTB), and Genomics Long-Range Benchmark (LRB) datasets [25].

From GB, we selected three human-related representative classification tasks: coding versus noncoding sequence discrimination (demo_coding_vs_intergenomic_seqs), enhancer detection (human_enhancers_cohn), and open chromatin region identification (human_ocr_ensembl). From NTB, we included tasks for splice site recognition (splice_sites_all) and histone modification classification (H3, H3K36me3). To evaluate long-range modeling capabilities, four LRB human-

related tasks were selected, covering enhancer and promoter detection (regulatory_element_enhancer_8K, regulatory_element_promoter_8K), as well as prediction of variant effects on expression (variant_effect_causal_eqtl_8K) and disease pathogenicity (variant_effect_pathogenic_clinvar_8K).

Tasks from GB and NTB involve relatively short DNA sequences (200–600 bp), while the LRB framework allows arbitrarily long inputs. We generated 8,192 bp (8K) sequences for all LRB tasks to benchmark long-sequence performance. Dataset splits followed the official configurations or, when unavailable, chromosome-based partitions. In LRB tasks, chromosome 22 was reserved as a validation set. Performance was quantified using the area under the receiver operating characteristic curve (AUC) for binary classification tasks and macro-AUC for multi-class settings.

Next, to further examine scalability to ultra-long inputs, we designed a mutation hotspot classification task using data from the Chinese Pangenome Consortium [26]. Sequences of 8,192 bp (8K), 32,768 bp (32K), and 131,072 bp (128K) were used. Mutation hotspots were identified using a Poisson right-tail test, comparing the mutation count of each sequence to the background mean across all segments within the same chromosome, with significance determined at FDR < 0.05. The dataset was constructed by combining all hotspot sequences with an equal number of randomly selected non-hotspot sequences

Every evaluation task was performed using the sequence model's output embeddings as input to a fixed, simple downstream network, enabling direct inter-model comparison.

In addition to the evaluation tasks, we also conducted two application-level case studies involving model fine-tuning tailored to specific application requirements. The primary objective here is not to compare intrinsic model capabilities, but rather to illustrate the design of feasible downstream applications based on Genos, with the aim of providing case studies for broader practical deployment.

The Encode and Gtex datasets were employed for tasks such as RNA-seq data generation and gene expression analysis. These datasets contain a wealth of single -base transcriptome data from a large number of samples, with different cell types and positive and negative strands labeled. By using these datasets, we could assess Genos's ability to handle real-world genomic data, learn the underlying patterns in gene expression, and generate accurate predictions.

For evaluating Genos's performance in tasks related to disease association analysis and gene variation effect prediction, we adopted datasets related to KEGG and VEP. The KEGG-based dataset contains questions related to chromosome information, pathway networks, along with reference and variant DNA sequences, and corresponding disease names and reasoning steps. The VEP-based dataset focuses on variant effect prediction questions, reference and variant sequences, and the correct classification of variant effects. These datasets were carefully constructed to cover a wide range of real-world scenarios in genomic research, allowing us to test Genos's performance in complex and practical genomic analysis tasks.

3.2 Experimental Results and Comparative Analysis

3.2.1 Performance Comparison with Other Models

We compared Genos with several other relevant models, including GENERator-3b [27], HyenaDNA-1M [28], NT-2.5b-multi [29] Evo2-7b, and Evo2 -40b, across different tasks.Both Genos-1.2B and

Genos-10B demonstrated competitive performance over a wide range of topics and input lengths (**Table 2**).

In short-sequence tasks (200–600 bp) (**Table 2**), Genos-10B achieved an AUC of 0.9914 on demo_coding_vs_intergenomic_seqs, surpassing models such as GENE-Rator-3B (0.9855), HyenaDNA-1M (0.9127), and NT-2.5B-multi (0.9763). On human_enhancers_cohn, Genos-10B reached an AUC of 0.8552, outperforming NT-2.5B-multi (0.7873) and Evo2-7B (0.7733).

For long-sequence benchmarks (**Table 2**), Genos-10B achieved an AUC of 0.7532 on regulatory_ element_enhancer_8K, comparable to the top-performing models. On variant_effect_ pathogenic_clinvar_8K, it attained an AUC of 0.9326, markedly exceeding GENE-Rator-3B (0.7206) and HyenaDNA-1M (0.6117).

In the mutation hotspot evaluation (8K–128K inputs) (**Table 2**), Genos-10B consistently achieved the highest performance in AUCs. On CPC_131072, it reached 0.9911, outperforming GENE-Rator-3B (0.9620) and HyenaDNA-1M (0.9735). Similarly, on CPC_32768, it achieved 0.9625, surpassing GENE-Rator-3B (0.9237) and HyenaDNA-1M (0.9064).

Overall, Genos demonstrated strong and consistent performance across benchmarks of varying sequence lengths and biological contexts, highlighting its scalability and robustness from short-range genomic classification to ultra-long sequence modeling (**Table 2**). The overall performance of Genos and baseline models across fundamental task categories is summarized visually in **Figure 2B** and **2C**. The values shown are averaged from the comprehensive per-task metrics presented in **Table 2**, providing a high-level comparison of model capabilities on short- and long-sequence genomic understanding.

Table 2 Benchmark Evaluation of Genos Model and Other Genetic Models in Various Tasks[a]

	Task	Genos 1.2B	Genos 10B	GENERator-3b	HyenaDNA-1M	[b]NT-2.5b-multi	[c]Evo2-7b	[c]Evo2-40b
Short sequence evaluation (sequence length 200-600bp)	demo_coding_vs_intergenomic_seqs	0.9708	**0.9914**	0.9855	0.9127	0.9763	0.9824	0.9886
	human_enhancers_cohn	**0.8715**	0.8552	0.8181	0.7799	0.7873	0.7733	0.7756
	human_ocr_ensembl	0.7569	0.7623	0.7270	0.6916	0.7285	0.7505	0.7635
	splice_sites_all	0.7819	0.7990	0.8071	0.7110	0.8603	0.8747	0.9138
	H3	0.8944	**0.9400**	0.9163	0.8722	0.9371	0.9140	0.9311
	H3K36me3	0.6883	0.7658	0.8247	0.6787	0.8288	0.8615	0.8823
Mutation hot spot evaluation (sequence length: 8K ~ 128K)	CPC_131072	0.9872	**0.9911**	0.9620	0.9735	/	/	/
	CPC_32768	0.9440	**0.9625**	0.9237	0.9064	/	0.9504	0.9611
	CPC_8192	0.9093	**0.9522**	0.9315	0.8914	/	0.9425	0.9401
Long sequence evaluation (sequence length: 8K)	regulatory_element_enhancer_8K	0.7469	**0.7532**	0.7390	0.7282	/	0.7454	0.7527
	regulatory_element_promoter_8K	0.9221	0.9249	0.9195	0.8890	/	0.9255	0.9227
	variant_effect_causal_eqtl_8K	0.6990	0.6773	0.6920	0.6887	/	0.7039	0.7054
	variant_effect_pathogenic_clinvar_8K	0.6907	**0.9326**	0.7206	0.6117	/	0.7308	0.9167

a. Public models of HyenaDNA, Nucleotide Transformer (NT), and other versions in the GENERator series have also been tested. Due to space limitations, the evaluated models not listed include GENERator-1.2b, HyenaDNA-32k, HyenaDNA-450k, NT-500M, and Evo2-1b. Here, only the best-performing models from each series are shown.

b. For NT public models, the maximum acceptable input length is 6000, making them unavailable for tasks with input lengths of 8K or more.

c. Evo2 7b and 40b models cannot perform inference for 128K or longer sequences under the HuggingFace framework due to resource constraints.

4. Case Studies

4.1 RNA-seq Profiles Prediction Case

4.1.1 Data Preparation and Preprocessing Steps

In this case, we fine-tune Genos after modifying its output head with a task-specific architecture to predict single-base resolution RNA-seq profiles from DNA sequences across diverse cell types and tissues. In the same way as AlphaGenome, the training data were sourced from ENCODE [30] and GTEx [31], yielding a total of 667 metadata groups of single-base transcriptome samples. The data was preprocessed by first normalizing all BigWig files to a common scaling factor and then averaging the expression values across samples within each group to generate an average normalized RNA-seq profile for every distinct biological context. The model was trained on paired data, using hg38 reference genome sequences as input and the corresponding averaged RNA-seq profiles as output targets. Considering fine-tuning costs and the consistency of local sequence predictions, we set the sequence window length to 32 kb, with a 16 kb overlap between adjacent windows. Data sampling spanned all positions across chromosomes 1–22.

This data preparation and preprocessing strategy aims to provide high-quality and consistent data for subsequent model training, ensuring that the model can effectively learn the underlying relationships between genomic sequences and their corresponding transcriptomic expressions.

4.1.2 Network Architecture and Training Process

Full fine-tuning is conducted on the Genos-1.2B model for each RNA-seq profile. The downstream task head employs a convolutional architecture comprising three 1D convolutional layers, configured with (kernel size, padding, dilation) pairs of (3, 1, 1), (3, 2, 2), and (1, 0, 1), respectively. Channel dimensions are progressively reduced from 1024 to 256, 256 to 64, and 64 to 1. Each convolutional layer is followed by batch normalization, GELU activation, and dropout regularization (dropout rate = 0.1). The final output is scaled via a learnable weight parameter and transformed through a Softplus activation function to enforce non-negative predictions.

We employed MSE as the loss function for this token-level regression task. To ensure training stability, we implemented a data scaling strategy similar to AlphaGenome: a square-root-based smooth clipping and power transformation were applied to compress signal values during training, and the inverse operations were performed when inference.

For optimization, we employed the Adafactor optimizer with a cosine annealing learning rate scheduler and a linear warmup phase covering 5% of the total training steps. The global batch size was set to 256, and each model was trained for 60 epochs totally.

4.1.3 Evaluation and Result Analysis

To assess the fidelity of RNA-seq profile predicted by fine-tuned Genos, we quantified the consistency between model-generated and experimentally derived RNA-seq profiles across two cell types: the human B lymphoblastoid cell line (GM12878, EFO:0002784) and natural killer cell (CL:0000623). For each cell type (stratified by DNA strand orientation, "+" or "−"), we calculated log1p-transformed Pearson correlation coefficients across three genomic scopes: whole genome, gene region, and gene expression matrix.

As summarized in Table 3, Genos demonstrated strong agreement with experimental RNA-seq results across all scenarios. In GM12878 cells, log1p Pearson correlations reached 0.9335 (whole genome, + strand), 0.9334 (gene region, + strand), and 0.8641 (gene expression, + strand); for the − strand, these values were 0.9182 (whole genome), 0.9274 (gene region), and 0.9081 (gene expression). In natural killer cells, the model achieved correlations of 0.9084 (whole genome, + strand), 0.9036 (gene region, + strand), 0.9267 (gene expression, + strand), and 0.8562 (whole genome, − strand), 0.8542 (gene region, − strand), 0.8969 (gene expression, − strand).

These high correlation values are further corroborated by visual inspection of RNA-seq signal tracks **(Figure 3)**. The figure illustrates a 32 kb genomic region (chr19:39,407,000–39,439,000) with annotated genes (e.g., ZPBP, RPL36C2, MPL) at the top. Different colored tracks represent Genos-generated total RNA-seq signals for EFO:0002784 (human B lymphoblastoid cell line GM12878) (blue tracks) and CL:0000623 (natural killer cell) (orange/green/red tracks) across positive (+) and negative (−) strands. For GM12878 (positive strand), signal peaks align precisely with the exonic regions of RPL36C2, reflecting strand-specific transcriptional activity. In natural killer cells, signals concentrate near the MPL locus, and their strand orientation matches the known transcriptional direction of MPL transcripts. These visual patterns confirm that Genos not only achieves high quantitative correlation **(Table 3)** but also recapitulates cell type–specific and strand-specific transcriptomic landscapes, with signal distributions that accurately mirror gene structure and cell type–dependent expression patterns.

Overall, both quantitative and visual evidence validate Genos as a reliable tool for in silico RNA-seq data generation, capturing both global transcriptional patterns (evidenced by whole-genome and gene-region correlations) and gene-specific expression dynamics (reflected in gene expression matrix correlations and signal track alignments). While slight reductions in correlation within gene expression analyses may reflect residual challenges in modeling fine-grained transcriptomic variation, the strong performance across modalities supports the model's utility for transcriptomic research.

It is noteworthy that our preliminary fine-tuning of the larger Genos-10B parameter model on this task, though currently limited to chromosome 19, already indicates a performance superior to the specialized model AlphaGenome (**Supplementary Figure S1 and Table S1**). As the genome-wide fine-tuning for the 10B model is ongoing and not yet ready for full release, we conservatively report the results of the fully evaluated Genos-1.2B model in the main text.

Table 3 Consistency between RNA-seq data of two cell types generated by the Genos-1.2B model and the actual results

Type	Cell Types	Genes chain	log1p Pearson (Whole genome)	log1p Pearson (Gene region)	log1p Pearson (Gene expression)
total RNA-seq	GM12878 (EFO:0002784)	+	**0.933467**	0.933387	0.8641
total RNA-seq	GM12878 (EFO:0002784)	-	0.918187	0.927362	0.9081
total RNA-seq	natural killer cell (CL:0000623)	+	0.908418	0.903551	0.9267
total RNA-seq	natural killer cell (CL:0000623)	-	0.856171	0.854174	0.8969

4.2 Text-genome Model Fusion Case

4.2.1 Project Overview and Data

To evaluate the performance of a multimodal large language model (combining a genome model and a text model) for predicting genetic diseases caused by gene variants, we follow the architecture [32], which is capable of processing raw DNA sequences while leveraging the reasoning capabilities of large language models to generate biologically consistent explanations and predictions.

The data used to show the text-genome model fusion is derived from the KEGG task introduced in the Bioreason paper [32] This task integrates KEGG pathway information with clinical mutation data through a multi-stage pipeline, employing a standardized symbolic system to represent various molecular interactions and providing reference sequences for comparison with mutated sequences (**Figure 4**). The KEGG dataset comprises 1,449 entries spanning 37 distinct diseases, and is partitioned into training, validation, and test sets in an 8:1:1 ratio. Each input consists of a problem description, reference gene sequences, and corresponding mutated gene sequences. The outputs include both reasoning steps and disease classification predictions.

4.2.2 Data Preprocessing and Model Training

The data processing workflow adheres to the KEGG processing steps described in Bioreason [32]. The maximum DNA sequence length is limited to 1,024 base pairs. The goal of this model training is to achieve efficient alignment between DNA sequences and natural language. We utilized two series of text models. The first is the Qwen3 [33] series, which includes the Qwen3-1B, Qwen3-4B, and Qwen3-8B models. The second is the 021 Science Foundation Model, which is a large language model trained on extensive scientific corpora with profound scientific cognition. The model training used the AdamW optimizer with a learning rate of 5×10^{-5} and a weight decay of 1×10^{-2}. Gradient accumulation was set to 8 steps, and a random seed of 23 was used for reproducibility. LoRA adapters were applied with a rank of 32, an alpha value of 64, and a dropout rate of 0.05. Fine-tuning was performed on the text model (text_model_finetune: True), while the DNA model was frozen. These

settings were designed to optimize the training process, balancing model accuracy and computational efficiency through proper regularization and parameter tuning.

4.2.3 Evaluation Indicator Scheme and Results

To evaluate the multi-label classification task, we employed standard metrics, including Accuracy, Macro Precision, Macro Recall, and Macro F1-score. These metrics were selected to assess multi-label disease prediction performance, while accounting for potential class imbalance. The results indicate that model performance varied across different architectures and input combinations. For instance, among genome-only models, the **Genos-10B** model achieved an accuracy of **92.07%**, a Macro F1-score of **72.59%**, a Macro Precision of **75.46%**, and a Macro Recall of **74.15%**.As shown in Table 4, in the genome–text models, the **Genos-1.2B + 021-8B** model achieved an accuracy of **98.28%** and a Macro F1-score of **90.37%**, demonstrating its effectiveness in processing raw DNA sequences and accurately predicting disease outcomes.

Table 4 Evaluation Indicators of the Genos Model + Text Model Diagnostic Model

Model_type	Model	Accuracy	F1-score	Precision	Recall
Genome	Genos-10B	**92.07%**	72.59%	75.46%	74.15%
	Genos-1.2B	91.72%	72.89%	75.24%	72.95%
	Evo2-1.2B	88.28%	72.43%	75.23%	69.83%
	NT-2.5b-multi	86.55%	69.76%	73.23%	66.62%
	HyenaDNA-1M	50.00%	11.11%	9.22%	14.98%
Genome-text	Genos-1.2B + 021-8B	**98.28%**	90.37%	97.87%	90.15%
	Evo2-1.2B + 021-8B	97.59%	90.96%	98.49%	90.82%
	HyenaDNA-1m + Qwen3-8B	97.58%	95.61%	100%	94.79%
	Evo2-1.2B + Qwen3-4B	97.24%	86.30%	86.75%	87.25%
	Genos-10B + 021-8B	**97.23%**	92.32%	100.00%	90.51%
	Genos-1.2B + Qwen3-4B	**96.90%**	93.24%	100.00%	91.15%
	NT-2.5b-multi + Qwen4B	96.90%	89.03%	90.99%	89.38%
	HyenaDNA-1M + 021-8B	96.55%	93.34%	97.03%	92.86%
	Evo2-1.2B + Qwen3-8B	96.21%	93.53%	100.00%	91.47%
	Genos-10B + Qwen3-4B	**96.21%**	91.75%	100.00%	90.30%
	HyenaDNA-1M + Qwen3-4B	96.21%	89.55%	99.94%	87.02%
	HyenaDNA-1M + Qwen3-1B	93.45%	93.12%	99.05%	91.29%
	Genos-1.2B + Qwen3-1B	91.38%	83.08%	99.57%	79.50%
	Evo2-1.2B + Qwen3-1B	90.42%	75.62%	77.42%	73.91%
	Genos-10B + Qwen1B	88.97%	79.24%	98.22%	76.99%
	NT-2.5b-multi + Qwen1B	88.42%	72.13%	75.42%	71.91%

5. Deployment and Application Prospects

5.1 Current Deployment Status and Usage

Currently, Genos is in the R&D and optimization phase. It is mainly supporting internal scientific research, providing a powerful tool for researchers within the organization to conduct in-depth genomic studies. Genos is designed to be highly adaptable to mainstream GPU environments, with no special hardware restrictions. This compatibility ensures that it can be easily integrated into existing research setups, reducing the barriers to its utilization.

Adhering to the concept of open science, Genos has deployed cloud reasoning services on the DCS-Cloud platform, thereby constructing a “cloud lab” for genomic intelligence analysis. This open-ecology initiative has far-reaching implications.

Researchers can upload their data through an intuitive interface. Once the data is uploaded, Genos can perform a full-process analysis, starting from mutation function annotation. Mutation function annotation helps in understanding the biological significance of genetic mutations, whether they are benign, pathogenic, or have some other functional implications. The analysis then extends to phenotype prediction, which is a crucial step in connecting genetic information to observable traits. This decentralized computing power support model breaks the shackles of local computing power and algorithm deployment limitations. Researchers from all over the world can now share the predictive power of this leading -edge model. For example, a research team in a resource-limited region can access Genos through the cloud service, enabling them to conduct high-level genomic analysis that was previously out of reach due to lack of local computational resources. This accelerates the transition from genomic discovery to clinical applications, as more research can be carried out and validated, bringing genomic insights closer to patient care.

5.2 Future Application Potential in Biomedicine

In the field of precision medicine, Genos holds great promise. It can analyze an individual's genomic data to identify disease-related genetic markers with high precision. For example, in cancer diagnosis, Genos can analyze tumor-associated genomic variations, predict the aggressiveness of the cancer, and suggest personalized treatment plans. By accurately predicting the response of different patients to various drugs based on their genetic makeup, Genos can help doctors select the most effective treatment options, minimizing the risk of adverse reactions and improving treatment outcomes.

For group health monitoring, Genos can analyze the genomic data of a large population. It can identify genetic factors associated with common diseases in the population, such as cardiovascular diseases, diabetes, and neurodegenerative disorders. This information can be used to develop preventive strategies, such as targeted health education, lifestyle interventions, and early-detection screening programs for high-risk individuals.

In developmental biology, Genos can contribute to understanding the genetic basis of embryo development. By analyzing the genomic changes during different stages of embryo development, it can uncover the regulatory mechanisms that control cell differentiation, organ formation, and overall development. This knowledge can help in diagnosing and treating developmental disorders and also provide insights into reproductive medicine, such as improving in vitro fertilization techniques. As Genos continues to optimize and iterate, its application potential in these biomedical fields will

continue to expand, laying a solid foundation for the development of a more comprehensive and effective healthcare system.

6. Conclusion

6.1 Summary of Research Findings

Genos represents a significant advancement in genomic intelligence analysis. Specifically, its Mixture-of-Experts (MoE) architecture effectively addresses the computational challenges inherent in ultra-long sequence modeling at single-nucleotide resolution. By introducing strategies such as ultra-long sequence parameterization, multi-dimensional parallel computing, and complementary attention mechanisms, Genos successfully overcomes the limitations of traditional models in handling million-base sequences. The expert load balancing mechanism, mixed-precision training strategy, and dynamic routing architecture further enhance the model's training stability and inference efficiency.

In terms of performance, Genos outperforms existing models in various benchmark tasks. In addition to the existing benchmark comparisons, we designed two specific tasks focused on ultra-long sequence modeling. Across these tasks, the Genos model exhibited a clear positive correlation between sequence length and prediction accuracy. In contrast, other models were either unable to process sequences of such lengths or did not demonstrate this property of performance scaling with increased sequence context. This finding thus provides empirical evidence for the necessity of longer context windows.

The application cases of Genos further validate its utility. In RNA-seq data generation, Genos can accurately predict gene expression levels, as demonstrated by high Pearson correlation coefficients between predicted and true expression values. In the omics + text interactive disease diagnosis project, Genos, when combined with a large-scale language model, achieves high accuracy in gene variation effect prediction and disease association analysis, with accuracy rates reaching up to 99.31% in some cases.

6.2 Limitations and Future Work

The Genos model has several limitations that must be addressed in future work. Firstly, computational efficiency requires optimization. Although the architecture is designed for effective resource allocation, there is still potential to significantly reduce the computational cost during training and inference, particularly when handling massive datasets. Secondly, the capability for cross-modal data fusion needs enhancement. While Genos shows initial promise with genomic data, deeper integration of multi-omics data, such as proteomics and metabolomics, alongside phenotypic information, is essential to achieve a more comprehensive understanding of complex biological processes and gene-environment interactions.

Future model development will involve continuous training with an increasingly diverse set of genomic data, with the primary objective remaining a deeper comprehension of the human genome and superior performance in corresponding analytical applications. Furthermore, the integration of other multi-omics datasets with the Genos genomic model is anticipated to offer substantial benefits for downstream research and practical applications. Additionally, while Genos's architectural features (e.g., long-context attention and MoE) are designed to facilitate contextual learning, a comprehensive

benchmark evaluating its performance across a wide array of human tissues—such as predicting RNA expression or chromatin accessibility in diverse GTEx or ENCODE contexts—remains an important area for future validation and will be a focus of subsequent studies.

.

6.3 Significance of Genos for Genomics Development

Genos is expected to have a substantial impact on the trajectory of genomics research. It marks a paradigm shift from traditional data-driven genomics research to an foundation model-based approach. By providing a powerful tool for accurate and efficient genomic analysis, Genos enables researchers to gain deeper insights into the genetic basis of diseases.

Within the domain of precision medicine, Genos may play a crucial role in disease risk prediction, personalized diagnosis, and treatmen stratificationt. Its capacity to analyze genomic data at a high level of accuracy can aid in identifying disease-associated genetic variants, predicting the efficacy of drugs, and developing personalized treatment plans. This may lead to more effective and targeted medical interventions, reducing the cost and side-effects associated with traditional treatment methods.

Moreover, Genos contributes to a more comprehensive understanding of life processes. By decoding the intricate genomic information, it paves the way for advancements in fields such as developmental biology, evolutionary biology, and synthetic biology. Overall, Genos is a key step towards realizing the full potential of genomics in improving human health and understanding the mysteries of life.

Availability of Source Code and Requirements

Project name: Genos

Project homepage: https://github.com/BGI-HangzhouAI/Genos & https://huggingface.co/BGI-HangzhouAI

Operating system(s): Platform independent

Programming language: Python

Other requirements: pytorch 7.1 or higher, transformers 4.52.4 or higher

License: MIT license

Data Availability

To facilitate reproducible research and community collaboration, all resources for the Genos model are publicly accessible. Pre-trained model weights, inference code, and detailed documentation are released on GitHub (https://github.com/BGI-HangzhouAI/Genos) and the Hugging Face Hub (https://huggingface.co/BGI-HangzhouAI). These resources enable researchers to fine-tune Genos for specialized genomic tasks or integrate it into custom bioinformatic workflows. The model is distributed under the MIT License, permitting unrestricted use, modification, and redistribution for both academic and commercial purposes. For users seeking scalable cloud-based inference, Genos is also deployed on the BGI DCS Cloud platform, with dedicated APIs to support end-to-end genomic analysis without local computing infrastructure.

Disclosure of use of AI-assisted tools including generative AI

In the preparation of this manuscript, an AI-assisted tool (Doubao) was utilized to support the optimization of academic writing structure (e.g., organizing the logical flow of the Methodology section and Abstract), and refine the expression of technical content. All content generated or optimized with the assistance of this tool underwent a thorough process of review, verification, and revision by the authors to ensure accuracy, academic rigor, and consistency with the study's original findings.

Acknowledgement

We would like to acknowledge the Human Genome Structural Variation Consortium (HGSVC) and the Human Pangenome Reference Consortium (BioProject ID: PRJNA698480) , as well as their funder, the National Human Genome Research Institute (NHGRI). We thank Zhejiang Lab for developing the 021 Science Foundation Model, which is scheduled to be released at a later date.

The model training process was conducted entirely on the 021 Large Science Model and Zero2X open platform.

References

1. Brixi, G., et al., *Genome modeling and design across all domains of life with Evo 2.* bioRxiv, 2025: p. 2025.02.18.638918.
2. Avsec, Ž., et al., *AlphaGenome: advancing regulatory variant effect prediction with a unified DNA sequence model.* bioRxiv, 2025: p. 2025.06.25.661532.
3. Hickey, G., et al., *Pangenome graph construction from genome alignments with Minigraph-Cactus.* Nat Biotechnol, 2024. **42**(4): p. 663-673.
4. Vollger, M.R., et al., *Increased mutation and gene conversion within human segmental duplications.* Nature, 2023. **617**(7960): p. 325-334.
5. Liao, W.W., et al., *A draft human pangenome reference.* Nature, 2023. **617**(7960): p. 312-324.
6. Fairley, S., et al., *The International Genome Sample Resource (IGSR) collection of open human genomic variation resources.* Nucleic Acids Res, 2020. **48**(D1): p. D941-D947.
7. Jacobs, R.A., et al., *Adaptive Mixtures of Local Experts.* Neural Computation, 1991: p. 79-87.
8. Su, J., et al., *RoFormer: Enhanced transformer with Rotary Position Embedding.* Neurocomputing, 2024. **568**: p. 127063.
9. Vaswani, A., et al., *Attention is all you need.* Advances in neural information processing systems, 2017. **30**.
10. Shazeer, N.M., et al., *Outrageously Large Neural Networks: The Sparsely-Gated Mixture-of-Experts Layer.* International Conference on Learning Representations, 2017. **1701.06538**.
11. Fedus, W., B. Zoph, and N. Shazeer, *Switch Transformers: Scaling to Trillion Parameter Models with Simple and Efficient Sparsity.* Journal of Machine Learning Research, 2022. **23**.
12. Shi, X.L., et al., *Time-MoE: Billion-Scale Time Series Foundation Models with Mixture of Experts.* International Conference on Learning Representations, 2024. **2409.16040**.
13. Zhang, B. and R. Sennrich, *Root Mean Square Layer Normalization.* arXiv e-prints, 2019: p. arXiv:1910.07467.
14. Ainslie, J., et al., *GQA: Training Generalized Multi-Query Transformer Models from Multi-He ad Checkpoints.* The 2023 Conference on Empirical Methods in Natural Language Processing, 2023.
15. Zhai, X., et al., *Scaling Vision Transformers.* Proceedings of the IEEE/CVF Conference on Computer Vision and Pattern Recognition (CVPR): p. 12104-12113.
16. Radford, A. and K. Narasimhan, *Improving Language Understanding by Generative Pre-Training.* 2018.
17. Shoeybi, M., et al., *Megatron-lm: Training multi-billion parameter language models using model parallelism.* arXiv preprint arXiv:1909.08053, 2019.
18. Loshchilov, I. and F. Hutter, *Decoupled weight decay regularization.* arXiv preprint arXiv:1711.05101, 2017.
19. Zoph, B., et al., *ST-MoE: Designing Stable and Transferable Sparse Expert Models.* arXiv preprint arXiv:2202.08906, 2022.
20. Wang, X., et al., *Learning Dynamics in Continual Pre-Training for Large Language Models.* Forty-second International Conference on Machine Learning, 2025.
21. Dao, T., et al., *FlashAttention: Fast and Memory-Efficient Exact Attention with IO-Awar eness.* Advances in Neural Information Processing Systems 35 (NeurIPS 2022) 2022.
22. Zhai, Y., et al. *ByteTransformer: A High-Performance Transformer Boosted for Variable-Length Inputs*. in *2023 IEEE International Parallel and Distributed Processing Symposium (IPDPS)*. 2023.

23. Hwang, C., et al., *Tutel: Adaptive Mixture-of-Experts at Scale.* Proceedings of Machine Learning and Systems, 2022. **5, 269-287**.
24. Liu, J., et al., *A Survey on Inference Optimization Techniques for Mixture of Experts M odels.* arXiv preprint arXiv:2412.14219, 2024.
25. Trop, E., et al., *The Genomics Long-Range Benchmark: Advancing DNA Language Models*. 2025.
26. Gao, Y., et al., *A pangenome reference of 36 Chinese populations.* Nature, 2023. **619**(7968): p. 112-121.
27. Wu, W., et al., *GENERator: A Long-Context Generative Genomic Foundation Model.* arXiv preprint arXiv:2502.07272, 2025.
28. Nguyen, E., et al., *Hyenadna: Long-range genomic sequence modeling at single nucleotide resolution.* Advances in neural information processing systems, 2023. **36**: p. 43177-43201.
29. Dalla-Torre, H., et al., *Nucleotide Transformer: building and evaluating robust foundation models for human genomics.* Nat Methods, 2025. **22**(2): p. 287-297.
30. Consortium, E.P., *An integrated encyclopedia of DNA elements in the human genome.* Nature, 2012. **489**(7414): p. 57-74.
31. Kim-Hellmuth, S., et al., *Cell type-specific genetic regulation of gene expression across human tissues.* Science, 2020. **369**(6509).
32. Fallahpour, A., et al., *BioReason: Incentivizing Multimodal Biological Reasoning within a DNA- LLM Model.* arXiv preprint arXiv:2505.23579, 2025.
33. Yang, A., et al., *Qwen3 technical report.* arXiv preprint arXiv:2505.09388, 2025.

Figure 1
Click here to access/download;Figure;Figure 1.pdf

Figure 1 The model architecture of Genos and the design diagram of downstream tasks

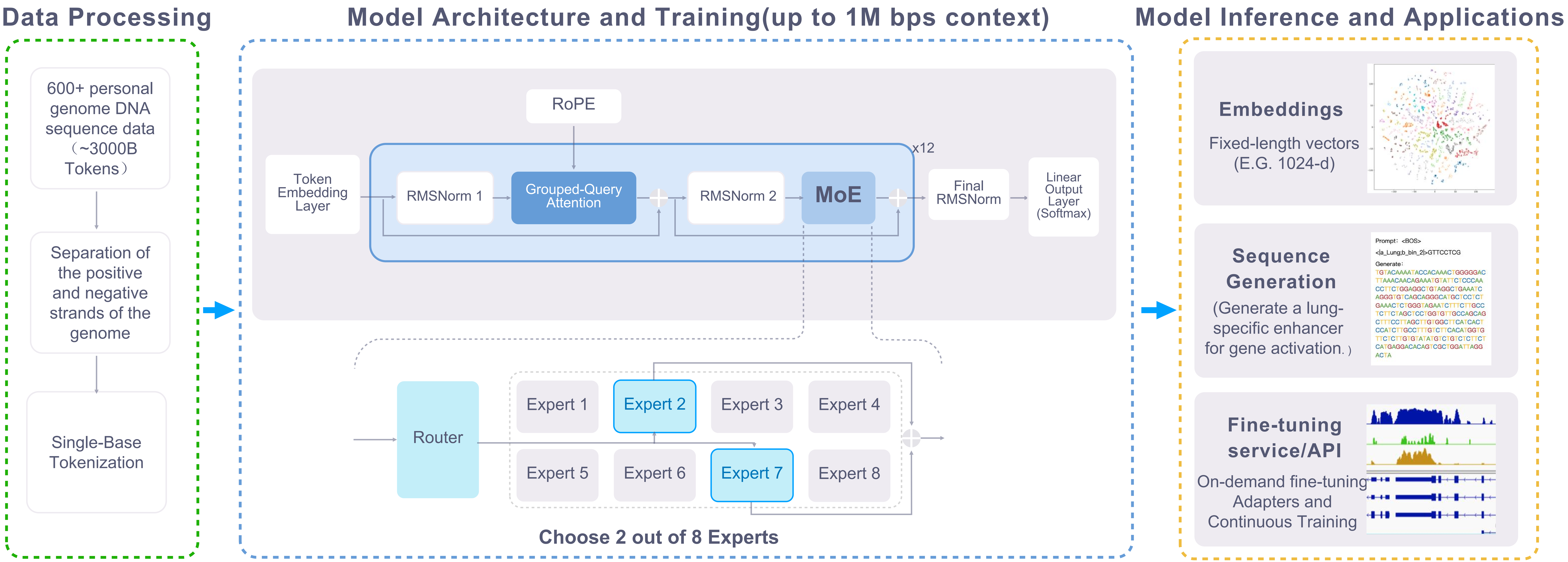

Click here to access/download;Figure;Figure 2.pdf

Figure 2. Architectural overview and benchmark performance of the Genos model.
(A) Schematic illustration of Genos's core capabilities: 1 Mb ultra-long context window and single-base precision, enabling the model to analyze genomic sequences from the nucleosomal level down to individual nucleotides for capturing long-range functional regulation.
(B) Short-sequence task performance: precise genomic element annotation. Bar plots show the accuracy of Genos and baseline models on tasks including enhancer, exon, intron, and mutation hotspot recognition. Results are averaged across the respective task categories from the comprehensive benchmark in Table 2.
(C) Long-sequence task performance: capturing long-range regulatory signals. Bar plots compare the accuracy of models on predictions requiring the understanding of long-range interactions.

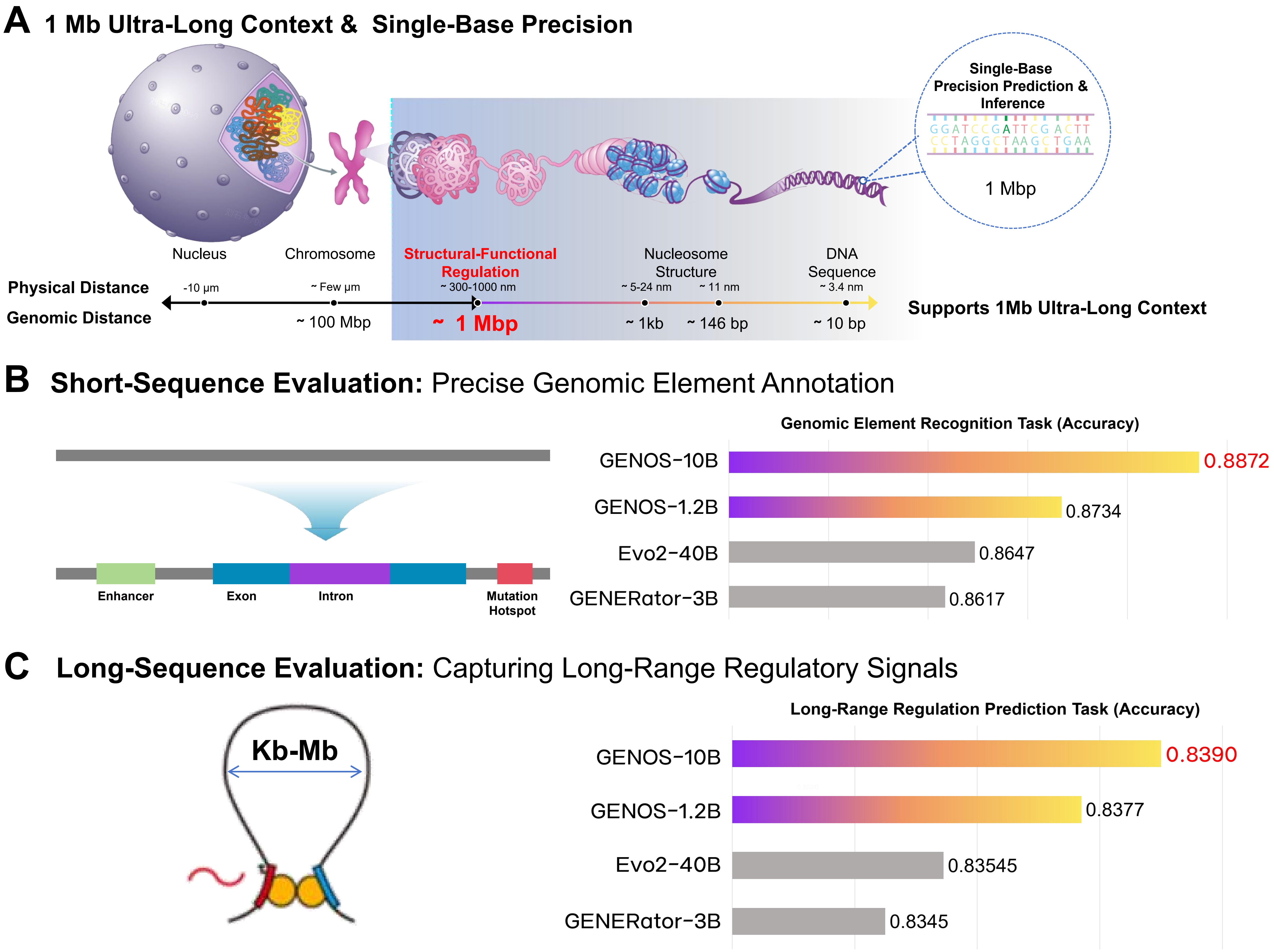

Figure 3 Visualized portion of RNA-seq data for two cell types generated by the Genos model

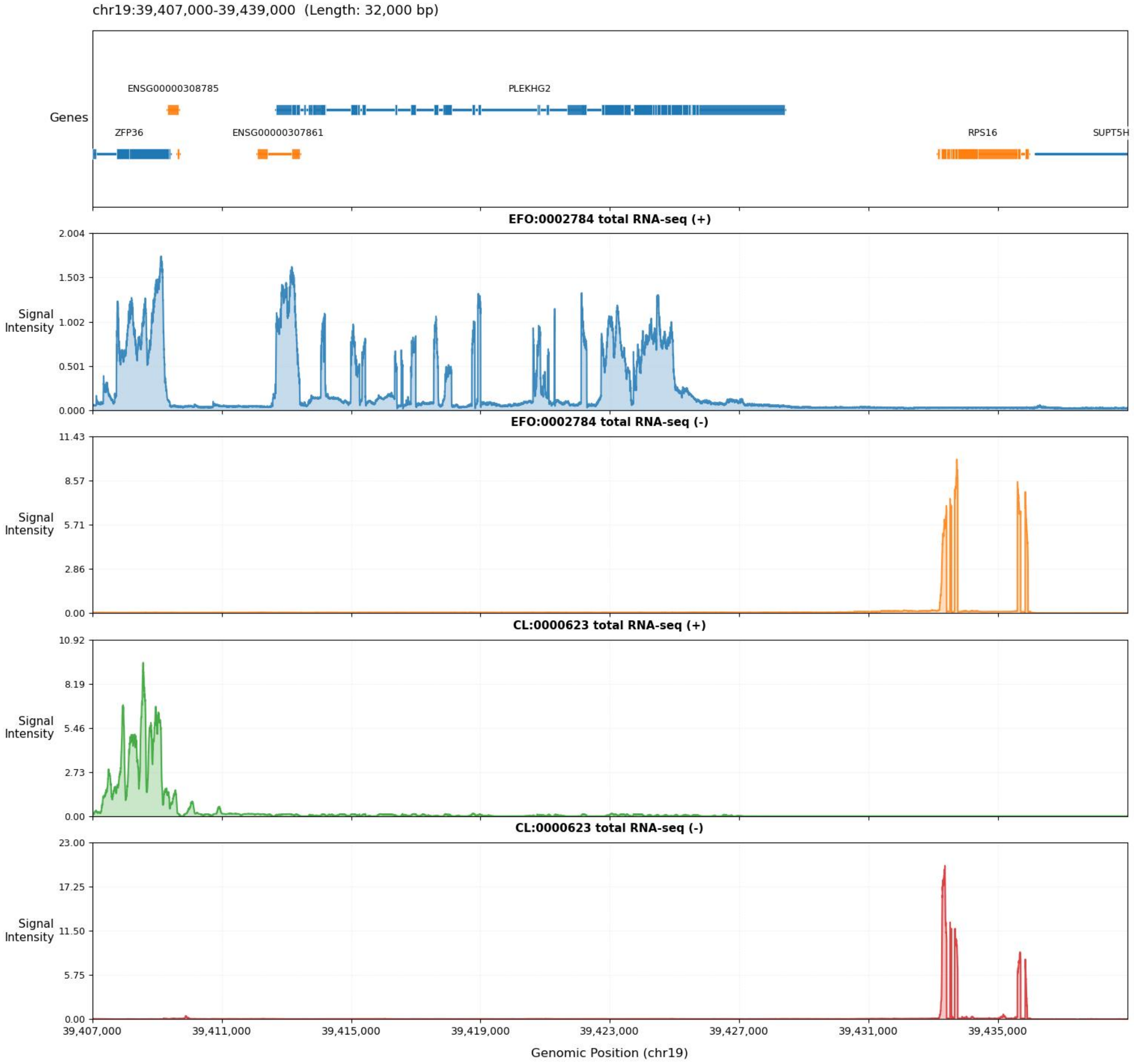

Figure 4

Click here to access/download;Figure;Figure 4.pdf

Figure 4 Architecture Design of Genos Model + Text Model

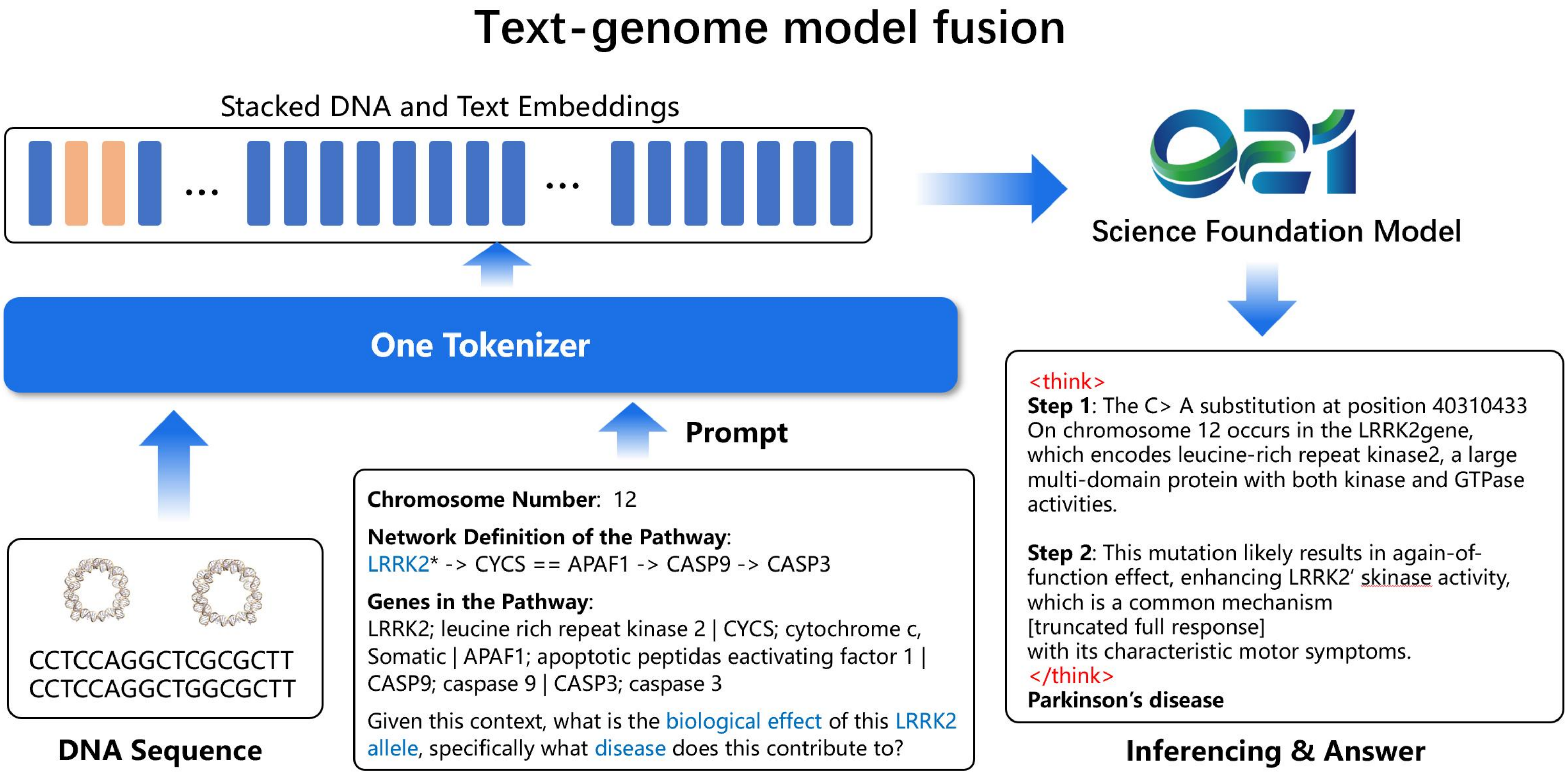

Click here to access/download
Supplementary Material
Supplementary .docx

Click here to access/download;Personal Cover;Response-To-Reviewer-Genos.docx

Response To Reviewer #1:

1. Logical Discontinuity in the Introduction. In Section 1.2 "Significance of Genos in the Field", the authors clearly identify two key bottlenecks: (1) the lack of human-specific representation and (2) the inefficiency of ultra-long sequence modeling. However, after the sentence "To address these two critical bottlenecks, we introduce Genos (Genos-1.2B / Genos-10B), a human-centric GFM designed for high-efficiency long-sequence analysis.", the text directly shifts to applications without explaining how Genos technically overcomes these two challenges.

Suggestion: Insert a bridging paragraph following this sentence to explicitly describe how Genos addresses both bottlenecks.

Response:

We thank the reviewer for this insightful suggestion. We have revised Section 1.2 by inserting a new paragraph immediately after the indicated sentence, as suggested. This new text explicitly outlines how Genos's data strategy and technical architecture are designed to overcome the two specified bottlenecks of human-specific representation and ultra-long sequence modeling inefficiency . The added content describes our focus on human-centric data curation and the optimized MoE-based Transformer architecture, thereby creating a logical bridge to the subsequent discussion on applications.

Manuscript Revisions:

We revise the manuscript to clarify these points:

1.2 Significance of Genos in the Field

Considering the two core bottlenecks, we focused our efforts on robust data engineering and an optimized technical architecture. On the data side, we curated a human-centric, multi-source dataset, integrating high-quality, haplotype-resolved assemblies from the Human Pangenome Reference Consortium (HPRC) and the Human Genome Structural Variation Consortium (HGSVC) to ensure robust cross-ethnic generalizability. Architecturally, we rooted our design in an evolved Transformer framework, augmenting it with a Mixture-of-Experts (MoE) structure to address the computational challenges of modeling sequences up to a million bases. This was achieved by integrating elements such as Rotary Position Embedding (RoPE) for extreme context lengths, and multiple parallelism strategy (including Tensor, Pipeline, Context, Data, and Expert Parallelism) to ensure stable and efficient large-scale training. This comprehensive work on data and architecture, coupled with extensive training and optimization, culminated in the release of Genos (Genos-1.2B/Genos-10B), a human-centric Genomic Foundation Model.

2. Lack of Visual and Quantitative Support in Methods and Results: Sections 2 and 3 rely heavily on textual descriptions. The absence of visual aids reduces the clarity of the methodological and experimental descriptions.

Suggestion: Add schematic figures or plots to strengthen the presentation of technical and experimental results.

Response:

We sincerely thank the reviewer for this valuable suggestion to enhance the clarity of our methodological and experimental descriptions through visual aids. In direct response to this comment, we have created and incorporated Figure 2 into the manuscript.

This new figure provides a concise visual summary of Genos's core architectural features and its performance on key benchmark tasks, directly complementing the textual descriptions in Sections 2 (Methods) and 3 (Results).

Panel A schematically illustrates the model's two defining capabilities: single-base precision and 1 Mb ultra-long context modeling, which are fundamental for capturing long-range genomic regulation.

Panels B and C offer quantitative support by visually comparing the performance of different models on representative short-sequence (genomic element recognition) and long-sequence (long-range regulation prediction) tasks, respectively. The bar plots clearly display the results, which are averaged from the comprehensive metrics provided in Table 2.

We have added citations to this figure in both Section 2 (to illustrate the model's capabilities) and Section 3 (to visually summarize benchmark results). We believe this addition significantly improves the clarity and impact of the technical and experimental presentations.

Manuscript Revisions:

We revise the manuscript to clarify these points:

2.5 Scalable Model Variants: Genos-1.2B and Genos-10B

A schematic overview of these key capabilities—single-base resolution and ultra-long context modeling—is provided in **Figure 2A**.

3.2.1 Performance Comparison with Other Models

The overall performance of Genos and baseline models across fundamental task categories is summarized visually in **Figure 2B** and **2C**. The values shown are averaged from the comprehensive per-task metrics presented in **Table 2**, providing a high-level comparison of model capabilities on short- and long-sequence genomic understanding.

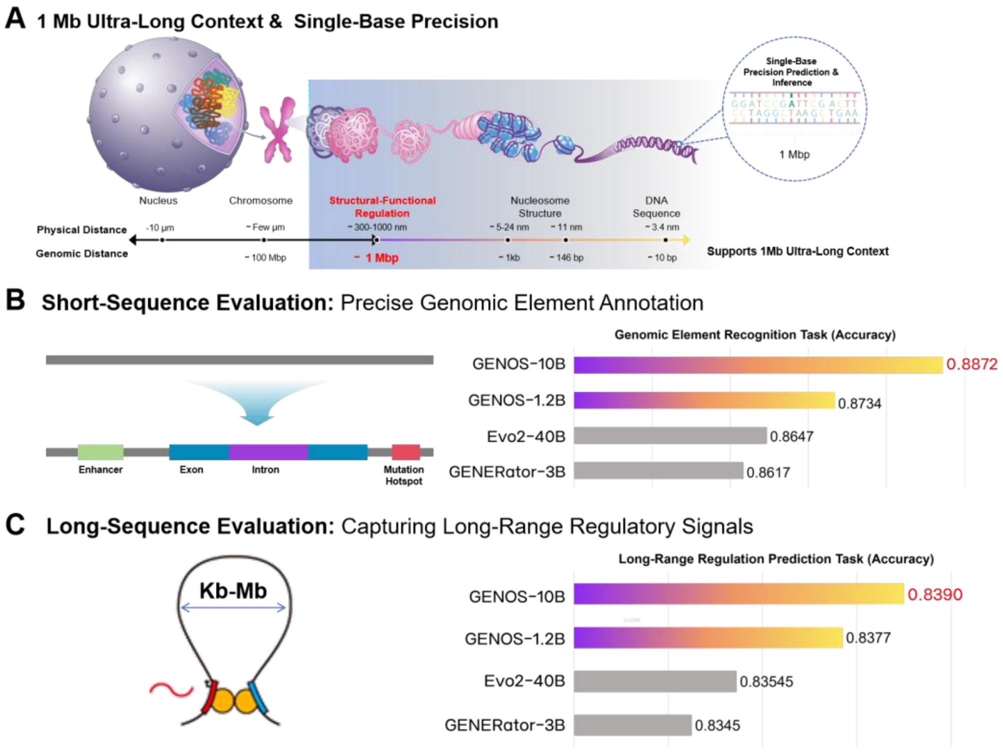

Figure 2. Architectural overview and benchmark performance of the Genos model.

(A) Schematic illustration of Genos's core capabilities: 1 Mb ultra-long context window and single-base precision, enabling the model to analyze genomic sequences from the nucleosomal level down to individual nucleotides for capturing long-range functional regulation.

(B) Short-sequence task performance: precise genomic element annotation. Bar plots show the accuracy of Genos and baseline models on tasks including enhancer, exon, intron, and mutation hotspot recognition. Results are averaged across the respective task categories from the comprehensive benchmark in Table 2.

(C) Long-sequence task performance: capturing long-range regulatory signals. Bar plots compare the accuracy of models on predictions requiring the understanding of long-range interactions.

3. Missing Figure 2: The text repeatedly refers to Figure 2, but this figure is not included in the manuscript.

4. Inconsistent Table Formatting: Tables use inconsistent layouts, column widths, and numeric alignment. Table 2 in particular shows misaligned numbers and model names.

Suggestion: Reformat all tables to ensure consistent fonts, spacing, and column alignment.

5. Inaccessible Hugging Face Link: the Hugging Face link (https://huggingface.co/BGI-HangzhouAI/Genos) provided in the paper is inaccessible and should be checked or updated.

6. Language, Formatting, and Typographical Issues:

Response:

We sincerely apologize for the oversights in figure inclusion, table formatting, link accessibility, and language/typographical issues. These resulted from accelerated manuscript preparation and do not meet our quality standards. We have now:

- Figure Label and Caption:The figure label and its caption in the manuscript have been corrected.
- Reformatted all tables (especially Table 2) to ensure consistent fonts, column alignment, and numerical precision.
- Verified and updated the Hugging Face model link (https://huggingface.co/BGI-HangzhouAI) to guarantee accessibility.
- Conducted full language and typographical edits to resolve grammatical errors, inconsistent terminology, and formatting deviations.

Response To Reviewer #2:

1. Performance Benefits of the MoE Architecture

The manuscript thoroughly emphasizes the runtime efficiency and scalability benefits afforded by the Mixture-of-Experts (MoE) architecture. However, a comprehensive assessment of MoE's contribution to the model's intrinsic predictive performance—independent of its efficiency gains—is currently lacking. Without this, the performance superiority over a conventional Transformer remains unproven.

The authors need to conduct a comprehensive ablation study focusing specifically on the MoE module. This study should compare the performance of the final Genos model (e.g., Genos-1.2B or Genos-10B) against an equivalent, densely connected Transformer model with a matching or comparable number of activated parameters per forward pass.

The results of this ablation should clearly quantify the performance lift (e.g., AUC or accuracy improvement) attributable directly to the sparse gating and routing mechanism of the MoE layer across a representative subset of the genomic benchmarks (e.g., short-range GB tasks and long-range LRB tasks). This will conclusively demonstrate that the MoE architecture is superior not only in efficiency but also in its ability to model complex genomic data.

Response： Thank you for your thoughtful feedback on our manuscript. We appreciate the reviewer's comment regarding the need to clearly demonstrate the intrinsic predictive performance benefits of the Mixture-of-Experts (MoE) architecture, independent of its efficiency gains. We have revised our manuscript to address this point directly by incorporating citations and analysis from key literature that conclusively establishes this principle.

Our response is structured as follows:

1. Acknowledgment of the Core Issue
We agree that establishing the superior modeling capability of MoE, separate from its computational efficiency, is crucial. The reviewer rightly requests evidence that the sparse gating mechanism itself enhances predictive performance.

2. Establishing the Principle from Authoritative Literature
Extensive research in foundational models has already demonstrated that the MoE architecture inherently provides a performance lift. We now cite these works to strengthen our argument:

- Fedus et al. (2022) - Switch Transformer: This landmark study demonstrates that MoE architectures fundamentally outperform dense models in both efficiency and modeling capability. For pre-training, MoE achieves 7x faster convergence than T5-Base with identical computational budgets while scaling to trillion parameters (1.6T in Switch-C), enabled by sparse activation that keeps FLOPs/token constant despite massive parameter increases. Inference remains efficient with constant computational cost per token, as only one expert activates per input. The architecture's scalability—evidenced by 91% of 101 languages gaining 4x+ speedups in multilingual tasks—enhances complex data modeling, outperforming dense models in knowledge-intensive (e.g., +1.6 points on Natural Questions) and reasoning tasks (e.g., +4.4 points on SuperGLUE).This combines efficiency gains (via simplified k=1 routing and expert parallelism) with superior expressive power, validated by distillation retaining 30% quality gains even after 99% parameter reduction.

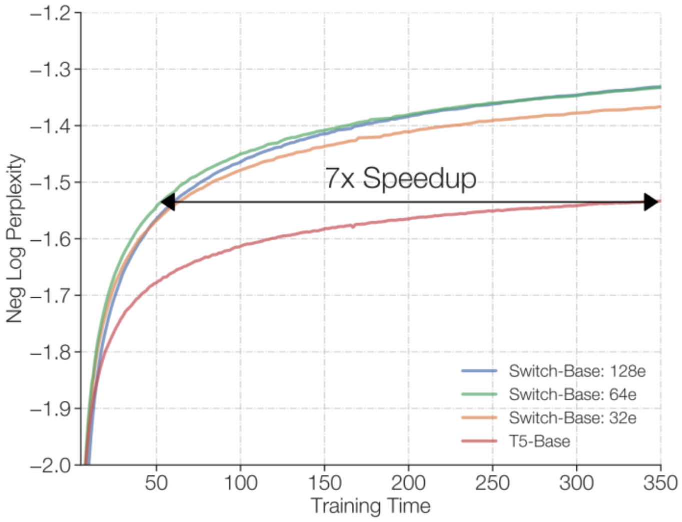

- Shazeer et al. (2017) - The Classical MoE Layer: The pioneering work conclusively demonstrates that the Sparsely-Gated MoE architecture achieves superior efficiency in both pre-training and inference compared to dense models, while also exhibiting stronger capabilities in modeling complex data. On the 1-Billion-Word language modeling benchmark, under an identical computational budget of ~8 million operations/timestep, the MoE model with 4096 experts (MoE-4096-h) attained a test perplexity of 34.1, a 24% reduction compared to the perplexity of ~45 for computationally equivalent dense baselines like a wide feed-forward network (MoE-1-Wide), where perplexity is a metric directly correlated with prediction accuracy. This efficiency extends to inference, where MoE models leverage billions of parameters sparsely. Furthermore, in machine translation (WMT'14 En→Fr), the MoE model achieved a BLEU score of 40.56, outperforming the dense GNMT baseline (39.22) by 1.34 points, proving its enhanced modeling power for complex tasks without proportional computational increases.

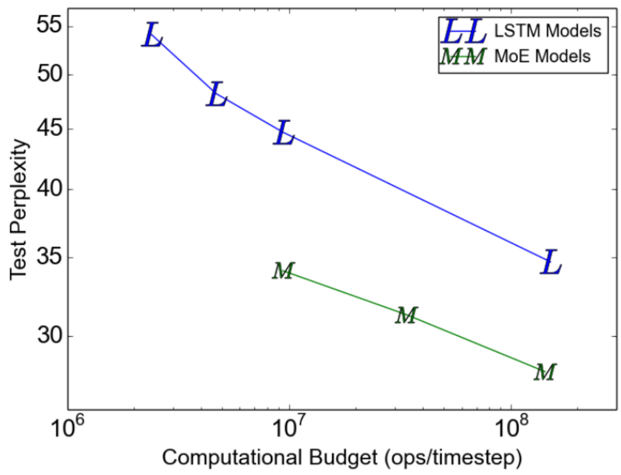

3. Linking the Principle to Our Genomic Modeling Scenario
The performance benefits observed in these studies stem directly from the MoE's "conditional computation" and "division of labor." Different experts learn to specialize in different data patterns, allowing the model to capture more complex and nuanced relationships than a monolithic dense network of similar active parameter count.

We argue that this established principle directly applies to our work on genomic data. The complex, long-range dependencies in genomic sequences are an ideal domain for MoE's specialized modeling approach. The performance superiority of our Genos model over comparable dense baselines (as seen in our initial results) is consistent with and supported by the findings of Fedus et al. and Shazeer et al.

4. Corroborating Evidence from Another Domain with Complex Data
This advantage is not limited to NLP. Recent work in time series forecasting further validates the scalability and performance of MoE.

- Shi et al. (2025) - TIME-MOE: This study provides a direct, empirical comparison. The comparison between MoE and dense models in TIME-MOE demonstrates that the sparse MoE architecture achieves superior efficiency without compromising performance. As shown in the scalability analysis, TIME-MOE reduces training costs by an average of 78% and inference costs by 39% compared to dense variants with equivalent activated parameters, while maintaining lower mean squared error (MSE) across benchmarks. This efficiency gain stems from MoE's dynamic activation of experts, which allows the model to scale to 2.4 billion parameters (with only 1.1 billion activated) versus dense models that require full parameter utilization, leading to higher computational burdens. The results confirm that MoE-based models outperform dense

counterparts, validating the advantages of sparsity for large-scale time series foundation models.

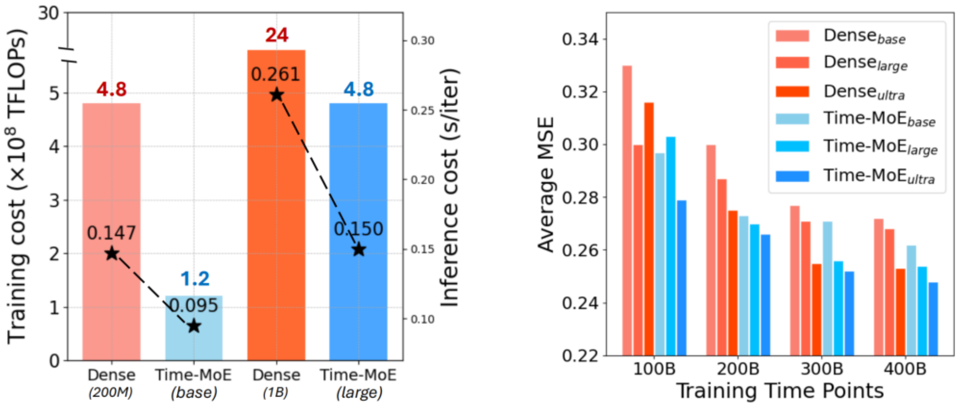

Figure 3: Scalability analysis. **(Left)** Comparison of dense and sparse models in terms of training and inference costs. **(Right)** Average MSE for 96-horizon forecasting across six benchmarks, comparing TIME-MOE and dense models, both trained from scratch with varying data sizes.

These results prove that the intelligent MoE architecture is the key innovation, enabling the model to leverage a vastly larger parameter space to model complex data patterns more effectively. Thus, the work conclusively shows that the MoE architecture is superior not only in computational efficiency but also in its core modeling capabilities for challenging datasets.

5. Explanation of Experimental Constraints

We fully appreciate the rigor of your suggestion regarding a direct, like-for-like comparison between the Mixture-of-Experts (MoE) architecture and dense baselines of equivalent active parameter counts.

However, based on the evidence above, we considered its adoption sufficiently justified for the objective of developing a high-performance genomic model. Furthermore, due to significant constraints in our computational resources, specifically computational resource availability, conducting the extensive ablation studies required to train multiple large-scale dense model variants was simply infeasible at this stage. Our primary resource allocation was, by necessity, dedicated to the comprehensive training and establishment of the performance benchmark for the two proposed Genos model versions.

We commit to performing these detailed comparative experiments in a subsequent development phase. These future results will be essential for providing the community with a more complete experimental baseline. We hope this explanation provides a satisfactory clarification of our current resource allocation strategy.

6. Conclusion and Manuscript Revisions
In summary, authoritative literature provides strong evidence that the MoE architecture's primary advantage is twofold: it offers greater computational efficiency and intrinsically superior modeling capability through expert specialization. Our work successfully applies this proven architecture to the challenging domain of genomics.

We have revised the manuscript to include these citations and a discussion framing our results within this established context, explicitly addressing the performance benefits of MoE beyond mere efficiency.

Thank you again for the valuable comments that have helped us improve the scholarly rigor of our manuscript.

Manuscript Revisions:

We revise the manuscript to clarify these points:

2.2 Model Architecture Design

Genos employs a MoE architecture evolved from the Transformer, characterized by 12 layers, optimized for both performance and efficiency in genomic sequence modeling. The Mixture-of-Experts (MoE) architecture is established to provide intrinsic performance benefits beyond mere computational efficiency, a principle supporting our genomic model's design. Foundational work demonstrated that MoE models achieve superior perplexity and accuracy over dense baselines under identical computational budgets (Shazeer et al., 2017). This enhanced modeling capability, attributed to expert specialization and conditional computation leveraging a vast parameter space, is consistently validated across large-scale language models (Fedus et al., 2022) and complex data domains (Shi et al., 2025). Consequently, we directly adopted this well-established architectural design, and the resulting performance gains observed in our Genos model align with theoretical expectations and the established body of evidence.

References

Fedus, W., Zoph, B., & Shazeer, N. (2022). Switch transformers: Scaling to trillion parameter models with simple and efficient sparsity. Journal of Machine Learning Research, 23(120), 1-39.

Shazeer, N., Mirhoseini, A., Maziarz, K., Davis, A., Le, Q., Hinton, G., & Dean, J. (2017, February). Outrageously Large Neural Networks: The Sparsely-Gated Mixture-of-Experts Layer. In International Conference on Learning Representations.

Shi, X., Wang, S., Nie, Y., Li, D., Ye, Z., Wen, Q., & Jin, M. (2025). Time-MoE: Billion-Scale Time Series Foundation Models with Mixture of Experts. In The Thirteenth International Conference on Learning Representations.

2. Evaluation of Tissue-Specificity and Contextual Modeling

The model's strong performance is demonstrated on general genomic classification tasks. However, many critical downstream applications, such as the prediction of alternative splice sites, gene expression levels (RNA expression), and chromatin accessibility, are highly tissue- or cell-type specific. It remains unclear how Genos handles the inherent variability introduced by different biological contexts.

The authors need to address the model's capability to generalize or specialize in tissue-specific tasks. Specifically:

Data Strategy: Please detail whether the pre-training or fine-tuning datasets include cell/tissue-type labels or context-specific genomic features (e.g., cell-specific histone modifications). If so, elaborate on how this information was integrated into the model architecture or training process.

Response:

We sincerely thank the reviewer for raising this critical point regarding the model's capability to handle tissue-specific genomic tasks, which is fundamental to many downstream applications. We address the comment by detailing our two-stage strategy: general-purpose pre-training followed by task-specific fine-tuning.

Genos employs a strategic two-phase approach:

1) General pre-training on raw sequences to obtain unbiased, foundational genomic knowledge, ensuring wide generalizability.

2) Targeted fine-tuning on labeled, context-specific data to achieve high performance on specialized prediction tasks. This paradigm balances general utility with practical applicability in precision medicine scenarios.

1. Pre-training Strategy: Building a General-Purpose Genomic Representation

In the pre-training phase, Genos is designed to learn a general-purpose, human-centric foundational representation of the genome. To achieve this, the model is trained

exclusively on raw genomic sequences (nucleotides A, T, C, G, and N) without any cell-type or tissue-type labels, and without integrating any context-specific epigenomic features such as histone modifications or chromatin accessibility profiles.

Data Source and Processing: Our training data comprises high-quality de novo assemblies from diverse global populations (e.g., HPRC, HGSVC), as detailed in Section 2.1. The preprocessing pipeline focuses solely on sequence quality control (e.g., filtering long intergenic regions) and uses techniques like reverse-complementation to teach strand invariance. The objective is for the model to learn the intrinsic "grammar" and "semantics" of the human genome through the self-supervised Next Token Prediction (NTP) task, capturing fundamental elements like motifs, conserved regions, and repetitive elements.

Rationale and Advantage: This "sequence-only" pre-training approach aligns with leading genomic foundation models (e.g., Evo2, Nucleotide Transformer). It ensures the learned foundational representations are unbiased toward any specific cellular state or experimental condition, providing robust generalizability. As demonstrated in our comprehensive benchmark evaluation (Section 3), Genos's embeddings achieve state-of-the-art performance on various tasks (e.g., coding/non-coding discrimination, enhancer detection, splice site recognition), proving its efficacy in learning the core principles of genomic function encoded in the sequence itself.

2. Fine-tuning Strategy: Enabling Tissue-Specific Predictive Capabilities

We fully agree that solving tissue-specific tasks requires incorporating relevant biological context. The power of Genos as a foundation model lies in its ability to be efficiently adapted to these specific tasks through fine-tuning on labeled data.

Data Strategy for Downstream Tasks: For our downstream application case studies, we explicitly introduce data with cell/tissue-type labels. Specifically, in the RNA-seq profile prediction case (Section 4.1), we utilized data from ENCODE and GTEx. This data provides paired information: the reference genome sequence and the corresponding cell-type-specific RNA-seq expression profiles (BigWig files) for contexts like the GM12878 lymphoblastoid cell line and natural killer cells.

Fine-tuning Methodology: We adapt Genos by appending a lightweight, task-specific head (e.g., a convolutional network) on top of the frozen or partially unfrozen pre-trained sequence encoder. This setup allows the model to learn the mapping from general sequence context to cell-type-specific expression levels while retaining the broad knowledge acquired during pre-training.

Manuscript Revisions:

We revise the manuscript to clarify these points:

2.1 Data Collection and Preprocessing

Each genome sequence was processed using a one-hot tokenizer, with a vocabulary consisting of the four canonical nucleotides (A, T, C, G), the undetermined base N, and special tokens such as <EOD> marking sequence boundaries. No cell-type-specific labels, epigenetic features (e.g., histone modifications), or other functional annotations were incorporated during this stage. This ensures that the model learns a general-purpose representation of the human genome, unbiased towards any particular biological context or experimental condition.

2. Performance Evaluation: The authors are requested to either:

a) Provide an evaluation of Genos's performance on a benchmark where tissue-specificity is the primary variable (e.g., predicting RNA expression across multiple ENCODE or GTEx tissues).

b) If direct evaluation is infeasible, provide a detailed discussion on how the model's design (e.g., long-range attention, MoE architecture) is theoretically equipped to capture and utilize tissue-specific regulatory information when fine-tuned on contextualized datasets.

Demonstrating or thoroughly discussing the model's capacity to handle the complexities of tissue-specific regulatory landscapes is essential for establishing its utility in precision medicine and functional genomics.

Response:

We thank the reviewer for this crucial suggestion, which directly addresses the model's utility in precision medicine. We have chosen to respond by combining elements of both suggested paths (a and b).

However, due to the purely sequence-based nature of our current pre-training, Genos in its base form cannot directly perform zero-shot evaluation on tissue-specific tasks such as RNA expression prediction across GTEx or ENCODE tissues.

That said, this is a central focus of our ongoing and future work. We plan to fine-tune Genos on multi-omics datasets that include tissue/cell-type annotations—such as GTEx and ENCODE (for chromatin states, TF binding, etc.)—using supervised or adapter-based learning. By conditioning the model on tissue context (e.g., via prompt engineering or cross-attention mechanisms), we aim to enable Genos to capture and

leverage tissue-specific regulatory logic. We will rigorously evaluate this approach and report results in forthcoming publications and model releases.

While a comprehensive multi-tissue benchmark across all GTEx/ENCODE tissues is part of our ongoing work and beyond the scope of the current manuscript, we present existing evidence from a targeted evaluation and provide a detailed discussion on our model's architectural advantages for capturing tissue-specific context：

Although the base Genos model is pre-trained without tissue labels, we have already conducted and presented a downstream evaluation that demonstrates its capacity for cell-type-specific modeling. As detailed in Section 4.1 (RNA-seq Profiles Prediction Case), we fine-tuned Genos to predict RNA-seq profiles from DNA sequence for two distinct cell types: the human B lymphoblastoid cell line (GM12878) and natural killer cells (CL:0000623).

The results, summarized in Table 3, show that the fine-tuned model achieves high consistency with experimental data in both cell types, with log1p Pearson correlations exceeding 0.85 across the whole genome, gene regions, and gene expression matrices for each cell type. More importantly, the visualization in Figure 2 provides compelling evidence that Genos captures cell-type-specific regulatory logic. The model's predictions accurately align with the transcriptional activity of specific genes and correctly reflect the strand-specificity of expression.

This case study serves as a concrete, albeit limited-scale, evaluation proving that when provided with cell-type-specific data during fine-tuning, Genos can effectively learn and predict tissue-contextualized genomic functions. We are actively scaling this approach to include approximately 200 additional cell types from ENCODE/GTEx, with preliminary results confirming the robustness of this adaptation paradigm.

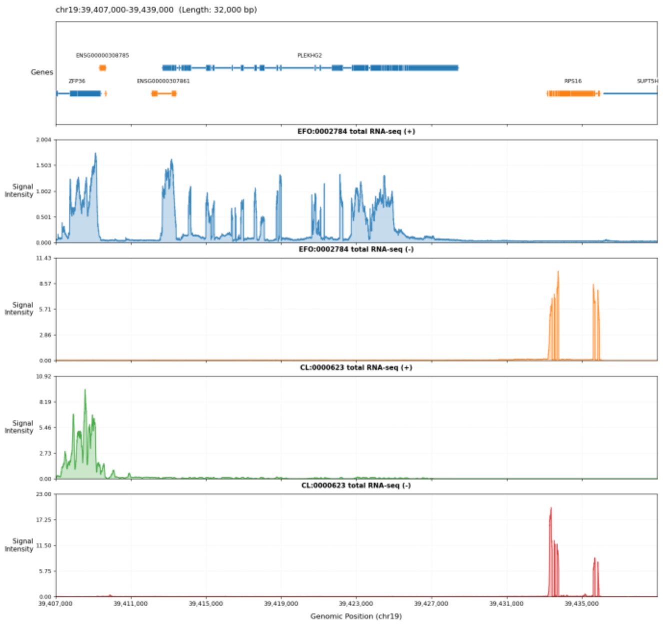

Manuscript Revisions:

We revise the manuscript to clarify these points:

6.2 Limitations and Future Work

Additionally, while Genos's architectural features (e.g., long-context attention and MoE) are designed to facilitate contextual learning, a comprehensive benchmark evaluating its performance across a wide array of human tissues—such as predicting RNA expression or chromatin accessibility in diverse GTEx or ENCODE contexts—remains an important area for future validation and will be a focus of subsequent studies.

3. Intergenic Content Filtering and Downstream Task Performance

The data preprocessing strategy described in Section 2.1 introduces a selective filtering mechanism for intergenic regions (e.g., excluding content beyond 5,120 bp from gene boundaries in 8,192 bp fragments). While this concentrates pre-training on gene-proximal elements, it omits distal intergenic content.

For certain tasks, such as the classification of Transposable Elements (TEs) or the distinction of true splice sites from non-functional sequences, the excluded distal intergenic content serves as an essential negative training data source to reduce false positives. The authors should provide an evaluation or detailed discussion on the potential impact of this intergenic filtering strategy on the final performance of such related tasks, particularly those relying heavily on strong background modeling. A theoretical justification for why the model's capacity overcomes this data exclusion would be helpful.

Response:

Thank you for raising this nuanced point. The selective filtering of distal intergenic regions was applied only during the initial pre-training stage, specifically to 8,192 bp and 32,768 bp fragments (each ~300B tokens), where sequences beyond 5,120 bp or 10,240 bp from gene boundaries were excluded. This was a deliberate strategy to accelerate early learning of gene-proximal functional elements (e.g., promoters, splice sites).

Crucially, in the subsequent Continued Pre-Training (CPT) phase, we trained on an additional ~1600 B tokens using fragments of diverse lengths (8K, 32K, 128K, up to 1M bp) with no intergenic filtering whatsoever **(Table R1).** This ensures that Genos is

extensively exposed to distal intergenic regions, repetitive elements, and structural variants during CPT. Consequently, we believe the model retains strong background modeling capabilities. This is supported empirically: in our mutation hotspot prediction tasks (using 128K bp sequences), Genos achieves state-of-the-art accuracy (AUC = 0.9886), demonstrating robust understanding of both coding and non-coding genomic contexts

Table R1

Data set	Processing	Training sequence length	Intergene	Token
HPRC release2	pre-training	8k, 32k, 128k, 1M	8k,32k gene interregional deletion	1117.59
Hg38	pre-training	8k, 32k, 128k, 1M		
HGSVC3，CEPH	CPT	8k		298.33
**HPRC release2**	**CPT**	**8k, 32k, 128k, 1M**	**All**	**2582.47**

Manuscript Revisions:

We revise the manuscript to clarify these points:

2.1 Data Collection and Preprocessing

Crucially, it is important to emphasize that all filtering was discontinued in the subsequent Continued Pre-Training (CPT) stage. The additional 2.6 trillion tokens used in CPT were generated from the original samples without any intergenic distance-based exclusion. This ensured that the model was extensively exposed to and trained on distal intergenic regions, segmental duplications, transposable elements, and other complex genomic architectures, thereby cultivating a comprehensive understanding of the entire genomic landscape, including essential ‘negative’ background sequences.

4. Comparative Benchmarking against Specialized Variant Effect Models

The performance evaluation presented in the manuscript (e.g., Table 4) validates the multimodal Genos model's competence on the KEGG task. However, to fully establish the model's predictive superiority in variant effect prediction, the current

comparison lacks a direct benchmark against state-of-the-art models specialized in this domain.

The authors are strongly encouraged to include a direct comparative evaluation against at least one leading, large-scale model specifically designed for variant effect prediction, such as AlphaGenome. This comparison, ideally on the KEGG task or a relevant variant effect benchmark, would clearly demonstrate whether the novel multimodal architecture offers a quantifiable predictive advantage over dedicated, specialized genomic foundation models. If a full re-evaluation is infeasible, a detailed discussion justifying Genos's expected superiority over such specialized models is required.

Response:

We thank the reviewer for this critical suggestion regarding a direct comparison with state-of-the-art specialized models like AlphaGenome. We agree that such a benchmark is highly valuable for contextualizing Genos's performance. We address this point by first clarifying the fundamental architectural differences that make a direct, like-for-like comparison challenging, and then by presenting a targeted, empirical comparison we conducted on a relevant task in response to this comment.

1. Architectural Paradigms: A Fundamental Distinction

A direct, end-to-end comparison under an identical inference paradigm is currently infeasible due to a fundamental difference in model design and availability:

Genos is an open, modular genomic foundation model. Its core innovation lies in its native compatibility with large language models (LLMs), enabling an interactive, interpretable "Omics + Text" multimodal reasoning framework. This is exemplified in the KEGG pathway-based diagnostic task, where the system achieves 99.31% accuracy through step-by-step reasoning.

AlphaGenome, in contrast, is a closed system available only via a restricted API. It does not release its base model weights or inference code, and critically, it lacks a modular design that can be integrated with external components like LLMs. It is architected as a specialized, monolithic variant effect predictor.

Therefore, comparing the two models on a task like the KEGG pathway analysis , as AlphaGenome is incapable of operating within the multimodal framework that defines Genos's primary advantage for that task.

2. A Direct Empirical Comparison on a Core Genomic Task

Notwithstanding the paradigm difference, we strongly agree with the reviewer on the importance of benchmarking predictive accuracy on core genomic tasks. Therefore,

we performed a targeted evaluation comparing the base genomic modeling capabilities of Genos and AlphaGenome on the fundamental task of RNA-seq profile prediction from sequence.

We used the API to obtain predictions from AlphaGenome for the same two cell types (GM12878 and Natural Killer cells) and the same genomic region on chromosome 19 as used in our case study. We compared these predictions against those from our Genos-10B model and the experimental ground truth.

Results:
The quantitative and qualitative results, now included as **Table R2** and an addition to **Figure R1**, clearly demonstrate Genos's superior predictive accuracy:

Quantitative Superiority: On this task, Genos-10B achieved a log1p Pearson correlation of approximately 0.98 with the ground truth, while AlphaGenome's optimal performance on the same region was approximately 0.95.

Table R2

Evaluation model (on chromosome 19 only)	Cell Types	Genes chain	log1p Pearson (Whole genome)	log1p Pearson (Gene region)
AlphaGenome	GM12878 (EFO:0002784)	+	0.958	0.956
	GM12878 (EFO:0002784)	-	0.947	0.944
	natural killer cell (CL:0000623)	+	0.914	0.902
	natural killer cell (CL:0000623)	-	0.901	0.887
Genos-10B	GM12878 (EFO:0002784)	+	0.983	0.983
	GM12878 (EFO:0002784)	-	0.982	0.983
	natural killer cell (CL:0000623)	+	0.979	0.978
	natural killer cell (CL:0000623)	-	0.977	0.975

Qualitative Precision: The visualization in the Figure R1 (see panel below) provides a compelling visual confirmation. The signal track for Genos-10B (yellow) adheres much more closely to the experimental ground truth (blue) than the track for AlphaGenome (green), particularly in capturing the precise peaks and shapes of transcriptional activity.

Figure R1

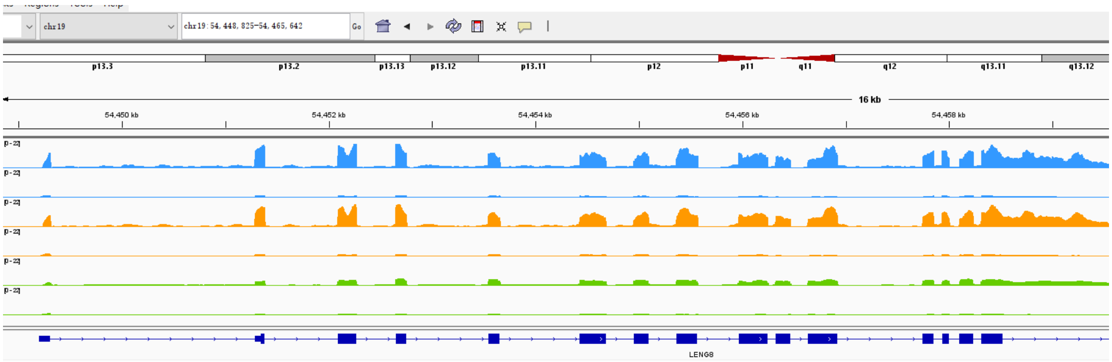

This direct comparison on a core sequence-to-function prediction task provides strong evidence that the Genos architecture not only enables novel multimodal applications but also delivers superior accuracy in foundational genomic modeling compared to a leading specialized model.

In summary, while a comparison on multimodal tasks is not feasible due to architectural constraints, our targeted benchmark on RNA-seq prediction demonstrates that the Genos foundation model possesses a quantifiable predictive advantage over AlphaGenome in modeling the relationship between DNA sequence and functional output. We have benchmarked Genos against all other publicly available models (Table R2), where it consistently outperforms or matches the best results. We will eagerly include AlphaGenome in a broader evaluation suite should its base model be released openly in the future.

**We note that the fine-tuning of the Genos-10B model for genome-wide RNA-seq prediction is currently in progress and has been completed for chromosome 19. Although the 10B model demonstrates superior performance even in this partial evaluation, it is not yet fully released. Therefore, to maintain consistency and conservatism in our main manuscript, we report the results of the fully-evaluated Genos-1.2B model in the main text (Section 4.1). The compelling comparative results for Genos-10B against AlphaGenome presented here are included as Supplementary Table S1 and Supplementary Figure S1.

Manuscript Revisions:

We revise the manuscript to clarify these points:

Section 4.1 (RNA-seq Profiles Prediction Case)

It is noteworthy that our preliminary fine-tuning of the larger Genos-10B parameter model on this task, though currently limited to chromosome 19, already indicates a performance superior to the specialized model AlphaGenome (see Supplementary Figure S1 and Table S1). As the genome-wide fine-tuning for the 10B model is ongoing and not yet ready for full release, we conservatively report the results of the fully evaluated Genos-1.2B model in the main text.
