# Supplementary material for: Genos: a human-centric genomic foundation model
Source: Gigascience. 2026 Jan 1;14:giaf132. doi: 10.1093/gigascience/giaf132 (PMC12755919; doi:10.1093/gigascience/giaf132)
Supplement: giaf132_Authors_Response_To_Reviewer_Comments_Original_Submission [file giaf132_authors_response_to_reviewer_comments_original_submission.pdf]

Author's Response To Reviewer Comments

Response To Reviewer #1:
1. Logical Discontinuity in the Introduction. In Section 1.2 "Significance of Genos in the Field", the authors clearly identify two key bottlenecks: (1) the lack of human-specific representation and (2) the inefficiency of ultra-long sequence modeling. However, after the sentence "To address these two critical bottlenecks, we introduce Genos (Genos-1.2B / Genos-10B), a human-centric GFM designed for high-efficiency long-sequence analysis.", the text directly shifts to applications without explaining how Genos technically overcomes these two challenges.
Suggestion: Insert a bridging paragraph following this sentence to explicitly describe how Genos addresses both bottlenecks.
Response：
We thank the reviewer for this insightful suggestion. We have revised Section 1.2 by inserting a new paragraph immediately after the indicated sentence, as suggested. This new text explicitly outlines how Genos's data strategy and technical architecture are designed to overcome the two specified bottlenecks of human-specific representation and ultra-long sequence modeling inefficiency . The added content describes our focus on human-centric data curation and the optimized MoE-based Transformer architecture, thereby creating a logical bridge to the subsequent discussion on applications.
Manuscript Revisions:
We revise the manuscript to clarify these points:
1.2 Significance of Genos in the Field
Considering the two core bottlenecks, we focused our efforts on robust data engineering and an optimized technical architecture. On the data side, we curated a human-centric, multi-source dataset, integrating high-quality, haplotype-resolved assemblies from the Human Pangenome Reference Consortium (HPRC) and the Human Genome Structural Variation Consortium (HGSVC) to ensure robust cross-ethnic generalizability. Architecturally, we rooted our design in an evolved Transformer framework, augmenting it with a Mixture-of-Experts (MoE) structure to address the computational challenges of modeling sequences up to a million bases. This was achieved by integrating elements such as Rotary Position Embedding (RoPE) for extreme context lengths, and multiple parallelism strategy (including Tensor, Pipeline, Context, Data, and Expert Parallelism) to ensure stable and efficient large-scale training. This comprehensive work on data and architecture, coupled with extensive training and optimization, culminated in the release of Genos (Genos-1.2B/Genos-10B), a human-centric Genomic Foundation Model.
2. Lack of Visual and Quantitative Support in Methods and Results: Sections 2 and 3 rely heavily on textual descriptions. The absence of visual aids reduces the clarity of the methodological and experimental descriptions.
Suggestion: Add schematic figures or plots to strengthen the presentation of technical and experimental results.
Response：
We sincerely thank the reviewer for this valuable suggestion to enhance the clarity of our methodological and experimental descriptions through visual aids. In direct response to this comment, we have created and incorporated Figure 2 into the manuscript.
This new figure provides a concise visual summary of Genos's core architectural features and its performance on key benchmark tasks, directly complementing the textual descriptions in Sections 2 (Methods) and 3 (Results).
Panel A schematically illustrates the model's two defining capabilities: single-base precision and 1 Mb ultra-long context modeling, which are fundamental for capturing long-range genomic regulation.
Panels B and C offer quantitative support by visually comparing the performance of different models on representative short-sequence (genomic element recognition) and long-sequence (long-range regulation prediction) tasks, respectively. The bar plots clearly display the results, which are averaged from the comprehensive metrics provided in Table 2.
We have added citations to this figure in both Section 2 (to illustrate the model's capabilities) and Section 3 (to visually summarize benchmark results). We believe this addition significantly improves the clarity and impact of the technical and experimental presentations.
Manuscript Revisions:
We revise the manuscript to clarify these points:

2.5 Scalable Model Variants: Genos-1.2B and Genos-10B
A schematic overview of these key capabilities—single-base resolution and ultra-long context modeling—is provided in Figure 2A.
3.2.1 Performance Comparison with Other Models
The overall performance of Genos and baseline models across fundamental task categories is summarized visually in Figure 2B and 2C. The values shown are averaged from the comprehensive per-task metrics presented in Table 2, providing a high-level comparison of model capabilities on short- and long-sequence genomic understanding.

Figure 2. Architectural overview and benchmark performance of the Genos model.
(A) Schematic illustration of Genos's core capabilities: 1 Mb ultra-long context window and single-base precision, enabling the model to analyze genomic sequences from the nucleosomal level down to individual nucleotides for capturing long-range functional regulation.
(B) Short-sequence task performance: precise genomic element annotation. Bar plots show the accuracy of Genos and baseline models on tasks including enhancer, exon, intron, and mutation hotspot recognition. Results are averaged across the respective task categories from the comprehensive benchmark in Table 2.
(C) Long-sequence task performance: capturing long-range regulatory signals. Bar plots compare the accuracy of models on predictions requiring the understanding of long-range interactions.

3. Missing Figure 2: The text repeatedly refers to Figure 2, but this figure is not included in the manuscript.
4. Inconsistent Table Formatting: Tables use inconsistent layouts, column widths, and numeric alignment. Table 2 in particular shows misaligned numbers and model names.
Suggestion: Reformat all tables to ensure consistent fonts, spacing, and column alignment.
5. Inaccessible Hugging Face Link: the Hugging Face link (https://huggingface.co/BGI-HangzhouAI/Genos) provided in the paper is inaccessible and should be checked or updated.
6. Language, Formatting, and Typographical Issues:
Response：
We sincerely apologize for the oversights in figure inclusion, table formatting, link accessibility, and language/typographical issues. These resulted from accelerated manuscript preparation and do not meet our quality standards. We have now:
λFigure Label and Caption:The figure label and its caption in the manuscript have been corrected.
λReformatted all tables (especially Table 2) to ensure consistent fonts, column alignment, and numerical precision.
λVerified and updated the Hugging Face model link (https://huggingface.co/BGI-HangzhouAI) to guarantee accessibility.
λConducted full language and typographical edits to resolve grammatical errors, inconsistent terminology, and formatting deviations.

Response To Reviewer #2:
1. Performance Benefits of the MoE Architecture
The manuscript thoroughly emphasizes the runtime efficiency and scalability benefits afforded by the Mixture-of-Experts (MoE) architecture. However, a comprehensive assessment of MoE's contribution to the model's intrinsic predictive performance—independent of its efficiency gains—is currently lacking. Without this, the performance superiority over a conventional Transformer remains unproven.
The authors need to conduct a comprehensive ablation study focusing specifically on the MoE module. This study should compare the performance of the final Genos model (e.g., Genos-1.2B or Genos-10B) against an equivalent, densely connected Transformer model with a matching or comparable number of activated parameters per forward pass.
The results of this ablation should clearly quantify the performance lift (e.g., AUC or accuracy improvement) attributable directly to the sparse gating and routing mechanism of the MoE layer across a representative subset of the genomic benchmarks (e.g., short-range GB tasks and long-range LRB tasks). This will conclusively demonstrate that the MoE architecture is superior not only in efficiency but also in its ability to model complex genomic data.

Response： Thank you for your thoughtful feedback on our manuscript. We appreciate the reviewer's comment regarding the need to clearly demonstrate the intrinsic predictive performance benefits of the Mixture-of-Experts (MoE) architecture, independent of its efficiency gains. We have revised our manuscript to address this point directly by incorporating citations and analysis from key literature that conclusively establishes this principle.

Our response is structured as follows:

1.Acknowledgment of the Core Issue

We agree that establishing the superior modeling capability of MoE, separate from its computational efficiency, is crucial. The reviewer rightly requests evidence that the sparse gating mechanism itself enhances predictive performance.

2.Establishing the Principle from Authoritative Literature

Extensive research in foundational models has already demonstrated that the MoE architecture inherently provides a performance lift. We now cite these works to strengthen our argument:

λFedus et al. (2022) - Switch Transformer: This landmark study demonstrates that MoE architectures fundamentally outperform dense models in both efficiency and modeling capability. For pre-training, MoE achieves 7x faster convergence than T5-Base with identical computational budgets while scaling to trillion parameters (1.6T in Switch-C), enabled by sparse activation that keeps FLOPs/token constant despite massive parameter increases. Inference remains efficient with constant computational cost per token, as only one expert activates per input. The architecture’s scalability—evidenced by 91% of 101 languages gaining 4x+ speedups in multilingual tasks—enhances complex data modeling, outperforming dense models in knowledge-intensive (e.g., +1.6 points on Natural Questions) and reasoning tasks (e.g., +4.4 points on SuperGLUE).This combines efficiency gains (via simplified k=1 routing and expert parallelism) with superior expressive power, validated by distillation retaining 30% quality gains even after 99% parameter reduction.

λShazeer et al. (2017) - The Classical MoE Layer: The pioneering work conclusively demonstrates that the Sparsely-Gated MoE architecture achieves superior efficiency in both pre-training and inference compared to dense models, while also exhibiting stronger capabilities in modeling complex data. On the 1-Billion-Word language modeling benchmark, under an identical computational budget of ~8 million operations/timestep, the MoE model with 4096 experts (MoE-4096-h) attained a test perplexity of 34.1, a 24% reduction compared to the perplexity of ~45 for computationally equivalent dense baselines like a wide feed-forward network (MoE-1-Wide), where perplexity is a metric directly correlated with prediction accuracy. This efficiency extends to inference, where MoE models leverage billions of parameters sparsely. Furthermore, in machine translation (WMT'14 En→Fr), the MoE model achieved a BLEU score of 40.56, outperforming the dense GNMT baseline (39.22) by 1.34 points, proving its enhanced modeling power for complex tasks without proportional computational increases.

3.Linking the Principle to Our Genomic Modeling Scenario

The performance benefits observed in these studies stem directly from the MoE's "conditional computation" and "division of labor." Different experts learn to specialize in different data patterns, allowing the model to capture more complex and nuanced relationships than a monolithic dense network of similar active parameter count.

We argue that this established principle directly applies to our work on genomic data. The complex, long-range dependencies in genomic sequences are an ideal domain for MoE's specialized modeling approach. The performance superiority of our Genos model over comparable dense baselines (as seen in our initial results) is consistent with and supported by the findings of Fedus et al. and Shazeer et al.

4.Corroborating Evidence from Another Domain with Complex Data

This advantage is not limited to NLP. Recent work in time series forecasting further validates the scalability and performance of MoE.

λShi et al. (2025) - TIME-MOE: This study provides a direct, empirical comparison. The comparison between MoE and dense models in TIME-MOE demonstrates that the sparse MoE architecture achieves superior efficiency without compromising performance. As shown in the scalability analysis, TIME-MOE reduces training costs by an average of 78% and inference costs by 39% compared to dense variants with equivalent activated parameters, while maintaining lower mean squared error (MSE) across benchmarks. This efficiency gain stems from MoE's dynamic activation of experts, which allows the model to scale to 2.4

billion parameters (with only 1.1 billion activated) versus dense models that require full parameter utilization, leading to higher computational burdens. The results confirm that MoE-based models outperform dense counterparts, validating the advantages of sparsity for large-scale time series foundation models.

These results prove that the intelligent MoE architecture is the key innovation, enabling the model to leverage a vastly larger parameter space to model complex data patterns more effectively. Thus, the work conclusively shows that the MoE architecture is superior not only in computational efficiency but also in its core modeling capabilities for challenging datasets.

5.Explanation of Experimental Constraints

We fully appreciate the rigor of your suggestion regarding a direct, like-for-like comparison between the Mixture-of-Experts (MoE) architecture and dense baselines of equivalent active parameter counts. However, based on the evidence above, we considered its adoption sufficiently justified for the objective of developing a high-performance genomic model. Furthermore, due to significant constraints in our computational resources, specifically computational resource availability, conducting the extensive ablation studies required to train multiple large-scale dense model variants was simply infeasible at this stage. Our primary resource allocation was, by necessity, dedicated to the comprehensive training and establishment of the performance benchmark for the two proposed Genos model versions.

We commit to performing these detailed comparative experiments in a subsequent development phase. These future results will be essential for providing the community with a more complete experimental baseline. We hope this explanation provides a satisfactory clarification of our current resource allocation strategy.

6.Conclusion and Manuscript Revisions

In summary, authoritative literature provides strong evidence that the MoE architecture's primary advantage is twofold: it offers greater computational efficiency and intrinsically superior modeling capability through expert specialization. Our work successfully applies this proven architecture to the challenging domain of genomics.

We have revised the manuscript to include these citations and a discussion framing our results within this established context, explicitly addressing the performance benefits of MoE beyond mere efficiency.

Thank you again for the valuable comments that have helped us improve the scholarly rigor of our manuscript.

Manuscript Revisions:

We revise the manuscript to clarify these points:

2.2 Model Architecture Design

Genos employs a MoE architecture evolved from the Transformer, characterized by 12 layers, optimized for both performance and efficiency in genomic sequence modeling. The Mixture-of-Experts (MoE) architecture is established to provide intrinsic performance benefits beyond mere computational efficiency, a principle supporting our genomic model's design. Foundational work demonstrated that MoE models achieve superior perplexity and accuracy over dense baselines under identical computational budgets (Shazeer et al., 2017). This enhanced modeling capability, attributed to expert specialization and conditional computation leveraging a vast parameter space, is consistently validated across large-scale language models (Fedus et al., 2022) and complex data domains (Shi et al., 2025). Consequently, we directly adopted this well-established architectural design, and the resulting performance gains observed in our Genos model align with theoretical expectations and the established body of evidence.

References

Fedus, W., Zoph, B., & Shazeer, N. (2022). Switch transformers: Scaling to trillion parameter models with simple and efficient sparsity. Journal of Machine Learning Research, 23(120), 1-39.

Shazeer, N., Mirhoseini, A., Maziarz, K., Davis, A., Le, Q., Hinton, G., & Dean, J. (2017, February). Outrageously Large Neural Networks: The Sparsely-Gated Mixture-of-Experts Layer. In International Conference on Learning Representations.

Shi, X., Wang, S., Nie, Y., Li, D., Ye, Z., Wen, Q., & Jin, M. (2025). Time-MoE: Billion-Scale Time Series Foundation Models with Mixture of Experts. In The Thirteenth International Conference on Learning Representations.

2. Evaluation of Tissue-Specificity and Contextual Modeling
The model's strong performance is demonstrated on general genomic classification tasks. However, many critical downstream applications, such as the prediction of alternative splice sites, gene expression levels (RNA expression), and chromatin accessibility, are highly tissue- or cell-type specific. It remains unclear how Genos handles the inherent variability introduced by different biological contexts.
The authors need to address the model's capability to generalize or specialize in tissue-specific tasks.
Specifically:
Data Strategy: Please detail whether the pre-training or fine-tuning datasets include cell/tissue-type labels or context-specific genomic features (e.g., cell-specific histone modifications). If so, elaborate on how this information was integrated into the model architecture or training process.
Response：
We sincerely thank the reviewer for raising this critical point regarding the model's capability to handle tissue-specific genomic tasks, which is fundamental to many downstream applications. We address the comment by detailing our two-stage strategy: general-purpose pre-training followed by task-specific fine-tuning.
Genos employs a strategic two-phase approach:
1) General pre-training on raw sequences to obtain unbiased, foundational genomic knowledge, ensuring wide generalizability.
2) Targeted fine-tuning on labeled, context-specific data to achieve high performance on specialized prediction tasks. This paradigm balances general utility with practical applicability in precision medicine scenarios.
1. Pre-training Strategy: Building a General-Purpose Genomic Representation
In the pre-training phase, Genos is designed to learn a general-purpose, human-centric foundational representation of the genome. To achieve this, the model is trained exclusively on raw genomic sequences (nucleotides A, T, C, G, and N) without any cell-type or tissue-type labels, and without integrating any context-specific epigenomic features such as histone modifications or chromatin accessibility profiles.
Data Source and Processing:Our training data comprises high-quality de novo assemblies from diverse global populations (e.g., HPRC, HGSVC), as detailed in Section 2.1. The preprocessing pipeline focuses solely on sequence quality control (e.g., filtering long intergenic regions) and uses techniques like reverse-complementation to teach strand invariance. The objective is for the model to learn the intrinsic "grammar" and "semantics" of the human genome through the self-supervised Next Token Prediction (NTP) task, capturing fundamental elements like motifs, conserved regions, and repetitive elements.
Rationale and Advantage:This "sequence-only" pre-training approach aligns with leading genomic foundation models (e.g., Evo2, Nucleotide Transformer). It ensures the learned foundational representations are unbiased toward any specific cellular state or experimental condition, providing robust generalizability. As demonstrated in our comprehensive benchmark evaluation (Section 3), Genos's embeddings achieve state-of-the-art performance on various tasks (e.g., coding/non-coding discrimination, enhancer detection, splice site recognition), proving its efficacy in learning the core principles of genomic function encoded in the sequence itself.
2. Fine-tuning Strategy: Enabling Tissue-Specific Predictive Capabilities
We fully agree that solving tissue-specific tasks requires incorporating relevant biological context. The power of Genos as a foundation model lies in its ability to be efficiently adapted to these specific tasks through fine-tuning on labeled data.
Data Strategy for Downstream Tasks: For our downstream application case studies, we explicitly introduce data with cell/tissue-type labels. Specifically, in the RNA-seq profile prediction case (Section 4.1), we utilized data from ENCODE and GTEx. This data provides paired information: the reference genome sequence and the corresponding cell-type-specific RNA-seq expression profiles (BigWig files) for contexts like the GM12878 lymphoblastoid cell line and natural killer cells.
Fine-tuning Methodology:We adapt Genos by appending a lightweight, task-specific head (e.g., a convolutional network) on top of the frozen or partially unfrozen pre-trained sequence encoder. This setup allows the model to learn the mapping from general sequence context to cell-type-specific expression levels while retaining the broad knowledge acquired during pre-training.

Manuscript Revisions:
We revise the manuscript to clarify these points:
2.1 Data Collection and Preprocessing
Each genome sequence was processed using a one-hot tokenizer, with a vocabulary consisting of the four

canonical nucleotides (A, T, C, G), the undetermined base N, and special tokens such as marking sequence boundaries. No cell-type-specific labels, epigenetic features (e.g., histone modifications), or other functional annotations were incorporated during this stage. This ensures that the model learns a general-purpose representation of the human genome, unbiased towards any particular biological context or experimental condition.

2. Performance Evaluation: The authors are requested to either:
a) Provide an evaluation of Genos's performance on a benchmark where tissue-specificity is the primary variable (e.g., predicting RNA expression across multiple ENCODE or GTEx tissues).
b) If direct evaluation is infeasible, provide a detailed discussion on how the model's design (e.g., long-range attention, MoE architecture) is theoretically equipped to capture and utilize tissue-specific regulatory information when fine-tuned on contextualized datasets.
Demonstrating or thoroughly discussing the model's capacity to handle the complexities of tissue-specific regulatory landscapes is essential for establishing its utility in precision medicine and functional genomics.
Response：
We thank the reviewer for this crucial suggestion, which directly addresses the model's utility in precision medicine. We have chosen to respond by combining elements of both suggested paths (a and b).
However, due to the purely sequence-based nature of our current pre-training, Genos in its base form cannot directly perform zero-shot evaluation on tissue-specific tasks such as RNA expression prediction across GTEx or ENCODE tissues.
That said, this is a central focus of our ongoing and future work. We plan to fine-tune Genos on multi-omics datasets that include tissue/cell-type annotations—such as GTEx and ENCODE (for chromatin states, TF binding, etc.)—using supervised or adapter-based learning. By conditioning the model on tissue context (e.g., via prompt engineering or cross-attention mechanisms), we aim to enable Genos to capture and leverage tissue-specific regulatory logic. We will rigorously evaluate this approach and report results in forthcoming publications and model releases.
While a comprehensive multi-tissue benchmark across all GTEx/ENCODE tissues is part of our ongoing work and beyond the scope of the current manuscript, we present existing evidence from a targeted evaluation and provide a detailed discussion on our model's architectural advantages for capturing tissue-specific context：
Although the base Genos model is pre-trained without tissue labels, we have already conducted and presented a downstream evaluation that demonstrates its capacity for cell-type-specific modeling. As detailed in Section 4.1 (RNA-seq Profiles Prediction Case), we fine-tuned Genos to predict RNA-seq profiles from DNA sequence for two distinct cell types: the human B lymphoblastoid cell line (GM12878) and natural killer cells (CL:0000623).
The results, summarized in Table 3, show that the fine-tuned model achieves high consistency with experimental data in both cell types, with log1p Pearson correlations exceeding 0.85 across the whole genome, gene regions, and gene expression matrices for each cell type. More importantly, the visualization in Figure 2 provides compelling evidence that Genos captures cell-type-specific regulatory logic. The model's predictions accurately align with the transcriptional activity of specific genes and correctly reflect the strand-specificity of expression.
This case study serves as a concrete, albeit limited-scale, evaluation proving that when provided with cell-type-specific data during fine-tuning, Genos can effectively learn and predict tissue-contextualized genomic functions. We are actively scaling this approach to include approximately 200 additional cell types from ENCODE/GTEx, with preliminary results confirming the robustness of this adaptation paradigm.

Manuscript Revisions:
We revise the manuscript to clarify these points:
6.2 Limitations and Future Work
Additionally, while Genos's architectural features (e.g., long-context attention and MoE) are designed to facilitate contextual learning, a comprehensive benchmark evaluating its performance across a wide array of human tissues—such as predicting RNA expression or chromatin accessibility in diverse GTEx or ENCODE contexts—remains an important area for future validation and will be a focus of subsequent studies.

3. Intergenic Content Filtering and Downstream Task Performance
The data preprocessing strategy described in Section 2.1 introduces a selective filtering mechanism for

intergenic regions (e.g., excluding content beyond 5,120 bp from gene boundaries in 8,192 bp fragments). While this concentrates pre-training on gene-proximal elements, it omits distal intergenic content.
For certain tasks, such as the classification of Transposable Elements (TEs) or the distinction of true splice sites from non-functional sequences, the excluded distal intergenic content serves as an essential negative training data source to reduce false positives. The authors should provide an evaluation or detailed discussion on the potential impact of this intergenic filtering strategy on the final performance of such related tasks, particularly those relying heavily on strong background modeling. A theoretical justification for why the model's capacity overcomes this data exclusion would be helpful.
Response：
Thank you for raising this nuanced point. The selective filtering of distal intergenic regions was applied only during the initial pre-training stage, specifically to 8,192 bp and 32,768 bp fragments (each ~300B tokens), where sequences beyond 5,120 bp or 10,240 bp from gene boundaries were excluded. This was a deliberate strategy to accelerate early learning of gene-proximal functional elements (e.g., promoters, splice sites).
Crucially, in the subsequent Continued Pre-Training (CPT) phase, we trained on an additional ~1600 B tokens using fragments of diverse lengths (8K, 32K, 128K, up to 1M bp) with no intergenic filtering whatsoever (Table R1). This ensures that Genos is extensively exposed to distal intergenic regions, repetitive elements, and structural variants during CPT. Consequently, we believe the model retains strong background modeling capabilities. This is supported empirically: in our mutation hotspot prediction tasks (using 128K bp sequences), Genos achieves state-of-the-art accuracy (AUC = 0.9886), demonstrating robust understanding of both coding and non-coding genomic contexts
Table R1
Data set Processing Training sequence length Intergene Token
HPRC release2 pre-training 8k, 32k, 128k, 1M 8k,32k gene interregional deletion 1117.59
Hg38 pre-training 8k, 32k, 128k, 1M
HGSVC3，CEPH CPT 8k 298.33
HPRC release2 CPT 8k, 32k, 128k, 1M All 2582.47

Manuscript Revisions:
We revise the manuscript to clarify these points:
2.1 Data Collection and Preprocessing
Crucially, it is important to emphasize that all filtering was discontinued in the subsequent Continued Pre-Training (CPT) stage. The additional 2.6 trillion tokens used in CPT were generated from the original samples without any intergenic distance-based exclusion. This ensured that the model was extensively exposed to and trained on distal intergenic regions, segmental duplications, transposable elements, and other complex genomic architectures, thereby cultivating a comprehensive understanding of the entire genomic landscape, including essential ‘negative’ background sequences.

4. Comparative Benchmarking against Specialized Variant Effect Models
The performance evaluation presented in the manuscript (e.g., Table 4) validates the multimodal Genos model's competence on the KEGG task. However, to fully establish the model's predictive superiority in variant effect prediction, the current comparison lacks a direct benchmark against state-of-the-art models specialized in this domain.
The authors are strongly encouraged to include a direct comparative evaluation against at least one leading, large-scale model specifically designed for variant effect prediction, such as AlphaGenome. This comparison, ideally on the KEGG task or a relevant variant effect benchmark, would clearly demonstrate whether the novel multimodal architecture offers a quantifiable predictive advantage over dedicated, specialized genomic foundation models. If a full re-evaluation is infeasible, a detailed discussion justifying Genos's expected superiority over such specialized models is required.
Response：
We thank the reviewer for this critical suggestion regarding a direct comparison with state-of-the-art specialized models like AlphaGenome. We agree that such a benchmark is highly valuable for contextualizing Genos's performance. We address this point by first clarifying the fundamental architectural differences that make a direct, like-for-like comparison challenging, and then by presenting a targeted, empirical comparison we conducted on a relevant task in response to this comment.
1. Architectural Paradigms: A Fundamental Distinction
A direct, end-to-end comparison under an identical inference paradigm is currently infeasible due to a

fundamental difference in model design and availability:
Genos is an open, modular genomic foundation model. Its core innovation lies in its native compatibility with large language models (LLMs), enabling an interactive, interpretable "Omics + Text" multimodal reasoning framework. This is exemplified in the KEGG pathway-based diagnostic task, where the system achieves 99.31% accuracy through step-by-step reasoning.
AlphaGenome, in contrast, is a closed system available only via a restricted API. It does not release its base model weights or inference code, and critically, it lacks a modular design that can be integrated with external components like LLMs. It is architected as a specialized, monolithic variant effect predictor.
Therefore, comparing the two models on a task like the KEGG pathway analysis , as AlphaGenome is incapable of operating within the multimodal framework that defines Genos's primary advantage for that task.
2. A Direct Empirical Comparison on a Core Genomic Task
Notwithstanding the paradigm difference, we strongly agree with the reviewer on the importance of benchmarking predictive accuracy on core genomic tasks. Therefore, we performed a targeted evaluation comparing the base genomic modeling capabilities of Genos and AlphaGenome on the fundamental task of RNA-seq profile prediction from sequence.
We used the API to obtain predictions from AlphaGenome for the same two cell types (GM12878 and Natural Killer cells) and the same genomic region on chromosome 19 as used in our case study. We compared these predictions against those from our Genos-10B model and the experimental ground truth.
Results:
The quantitative and qualitative results, now included as Table R2 and an addition to Figure R1, clearly demonstrate Genos's superior predictive accuracy:
Quantitative Superiority:On this task, Genos-10B achieved a log1p Pearson correlation of approximately 0.98 with the ground truth, while AlphaGenome's optimal performance on the same region was approximately 0.95.
Table R2
Evaluation model
(on chromosome 19 only) Cell Types Genes chain log1p Pearson (Whole genome) log1p Pearson (Gene region)
AlphaGenome GM12878 (EFO:0002784) + 0.958 0.956
GM12878 (EFO:0002784) - 0.947 0.944
natural killer cell (CL:0000623) + 0.914 0.902
natural killer cell (CL:0000623) - 0.901 0.887
Genos-10B GM12878 (EFO:0002784) + 0.983 0.983
GM12878 (EFO:0002784) - 0.982 0.983
natural killer cell (CL:0000623) + 0.979 0.978
natural killer cell (CL:0000623) - 0.977 0.975

Qualitative Precision:The visualization in the Figure R1 (see panel below) provides a compelling visual confirmation. The signal track for Genos-10B (yellow) adheres much more closely to the experimental ground truth (blue) than the track for AlphaGenome (green), particularly in capturing the precise peaks and shapes of transcriptional activity.
Figure R1

This direct comparison on a core sequence-to-function prediction task provides strong evidence that the Genos architecture not only enables novel multimodal applications but also delivers superior accuracy in foundational genomic modeling compared to a leading specialized model.
In summary, while a comparison on multimodal tasks is not feasible due to architectural constraints, our targeted benchmark on RNA-seq prediction demonstrates that the Genos foundation model possesses a quantifiable predictive advantage over AlphaGenome in modeling the relationship between DNA sequence and functional output. We have benchmarked Genos against all other publicly available models (Table R2), where it consistently outperforms or matches the best results. We will eagerly include AlphaGenome in a broader evaluation suite should its base model be released openly in the future.
**We note that the fine-tuning of the Genos-10B model for genome-wide RNA-seq prediction is currently in progress and has been completed for chromosome 19. Although the 10B model demonstrates superior performance even in this partial evaluation, it is not yet fully released. Therefore, to maintain consistency and conservatism in our main manuscript, we report the results of the fully-evaluated Genos-1.2B model in

the main text (Section 4.1). The compelling comparative results for Genos-10B against AlphaGenome presented here are included as Supplementary Table S1 and Supplementary Figure S1.
Manuscript Revisions:
We revise the manuscript to clarify these points:
Section 4.1 (RNA-seq Profiles Prediction Case)
It is noteworthy that our preliminary fine-tuning of the larger Genos-10B parameter model on this task, though currently limited to chromosome 19, already indicates a performance superior to the specialized model AlphaGenome (see Supplementary Figure S1 and Table S1). As the genome-wide fine-tuning for the 10B model is ongoing and not yet ready for full release, we conservatively report the results of the fully evaluated Genos-1.2B model in the main text.

Editorial Manager®

Cookie Preference Center

We use cookies which are necessary to make our site work. We may also use additional cookies to analyze, improve and personalize our content and your digital experience. For more information, see our Cookie Policy.

You may choose not to allow some types of cookies. However, blocking some types may impact your experience of our site and the services we are able to offer. See the different category headings below to find out more or change your settings.

Allow all

Manage Consent Preferences

Strictly Necessary Cookies

Always active

These cookies are necessary for the website to function and cannot be switched off in our systems. They are usually only set in response to actions made by you which amount to a request for services, such as setting your privacy preferences, logging in or filling in forms. You can set your browser to block or alert you about these cookies, but some parts of the site will not then work.

Functional Cookies

These cookies enable the website to provide enhanced functionality and personalisation. They may be set by us or by third party providers whose services we have added to our pages. If you do not allow these cookies then some or all of these services may not function properly.

Performance Cookies

These cookies allow us to count visits and traffic sources so we can measure and improve the performance of our site. They help us to know which pages are the most and least popular and see how visitors move around the site.

Targeting Cookies

These cookies may be set through our site by our advertising partners. They may be used by those companies to build a profile of your interests and show you relevant adverts on other sites. If you do not allow these cookies, you will experience less targeted advertising.

Cookie List

Clear

Apply Cancel
Consent Leg.Interest

Confirm my choices

Powered by onetrus
