# Supplementary material for: Genos: a human-centric genomic foundation model
Source: Gigascience. 2026 Jan 1;14:giaf132. doi: 10.1093/gigascience/giaf132 (PMC12755919; doi:10.1093/gigascience/giaf132)
Supplement: giaf132_Reviewer_1_Report_Original_Submission — Xing Zheng -- 10/15/2025 [file giaf132_reviewer_1_report_original_submission.pdf]

Reviewer Report

Title: Genos: A Human-Centric Genomic Foundation Model

Version: Original Submission **Date:** 10/15/2025

Reviewer name: Xing Zheng

Reviewer Comments to Author:

Reviewer Comments for Manuscript GIGA-D-25-00428
Title: Genos: A Human-Centric Genomic Foundation Model
Recommendation: Major Revision
The manuscript has conducted many meaningful explorations and provides valuable insights into the development of genomic foundation models; however, the main issues lie in the writing and presentation of the paper. It is recommended for acceptance after careful refinement and polishing of the manuscript.
General Assessment: The manuscript presents a novel and technically promising work that introduces Genos, a human-centric genomic foundation model. The study demonstrates substantial contributions in model architecture (MoE and ultra-long sequence modeling), training data curation (HPRC and HGSVC), and downstream applications (RNA-seq prediction and multimodal gene-text reasoning). However, the paper requires major revision before it can be considered for publication. Several issues need to be addressed, including structural incoherence between sections, repetitive sentences, missing figures, and inconsistent table formatting. Moreover, numerous linguistic and formatting errors remain throughout the text.
Major Comments:
1. Logical Discontinuity in the Introduction. In Section 1.2 "Significance of Genos in the Field", the authors clearly identify two key bottlenecks: (1) the lack of human-specific representation and (2) the inefficiency of ultra-long sequence modeling. However, after the sentence "To address these two critical bottlenecks, we introduce Genos (Genos-1.2B / Genos-10B), a human-centric GFM designed for high-efficiency long-sequence analysis.", the text directly shifts to applications without explaining how Genos technically overcomes these two challenges.
Suggestion: Insert a bridging paragraph following this sentence to explicitly describe how Genos addresses both bottlenecks.
2. Lack of Visual and Quantitative Support in Methods and Results: Sections 2 and 3 rely heavily on textual descriptions. The absence of visual aids reduces the clarity of the methodological and experimental descriptions.
Suggestion: Add schematic figures or plots to strengthen the presentation of technical and experimental results.
3. Missing Figure 2: The text repeatedly refers to Figure 2, but this figure is not included in the manuscript.
4. Inconsistent Table Formatting: Tables use inconsistent layouts, column widths, and numeric alignment. Table 2 in particular shows misaligned numbers and model names.

Suggestion: Reformat all tables to ensure consistent fonts, spacing, and column alignment.
5. Inaccessible Hugging Face Link: the Hugging Face link (https://huggingface.co/BGI-HangzhouAI/Genos) provided in the paper is inaccessible and should be checked or updated.
6. Language, Formatting, and Typographical Issues:
Location Original Text Correction
p.1 Abstract large-sclae Mixture of Experts (MoE) large-scale
p.2 Section 1.1 phenotyperegulatory networks phenotype regulatory
p.4 Section 2.1 Centre d'Etude du Polymophisme Humain Polymorphisme
p.4 Section 2.2 Genos adopted MoE archtecture architecture
p.6 Section 2.4 avalable through its hugginface service available
p.x Section 5.1 Huada DCSCloud ???
p.x Section 6.3 fundation model - based approach foundation model-based
p.x Section 6.3 treatmen stratificationt treatment
Please check the entire manuscript for typographical errors and unnecessary spaces in compound terms, e.g., 'multi - omics', 'RNA - seq', 'gene - region', and 'DNA - LLM combined models'.

Methods

Are the methods appropriate to the aims of the study, are they well described, and are necessary controls included? Choose an item.

Conclusions

Are the conclusions adequately supported by the data shown? Choose an item.

Reporting Standards

Does the manuscript adhere to the journal's guidelines on minimum standards of reporting? Choose an item.

Choose an item.

Statistics

Are you able to assess all statistics in the manuscript, including the appropriateness of statistical tests used? Choose an item.

Quality of Written English

Please indicate the quality of language in the manuscript: Choose an item.

Declaration of Competing Interests

Please complete a declaration of competing interests, considering the following questions:

- Have you in the past five years received reimbursements, fees, funding, or salary from an organisation that may in any way gain or lose financially from the publication of this manuscript, either now or in the future?
- Do you hold any stocks or shares in an organisation that may in any way gain or lose financially from the publication of this manuscript, either now or in the future?
- Do you hold or are you currently applying for any patents relating to the content of the manuscript?
- Have you received reimbursements, fees, funding, or salary from an organization that holds or has applied for patents relating to the content of the manuscript?
- Do you have any other financial competing interests?
- Do you have any non-financial competing interests in relation to this paper?

If you can answer no to all of the above, write 'I declare that I have no competing interests' below. If your reply is yes to any, please give details below.

I declare that I have no competing interests

I agree to the open peer review policy of the journal. I understand that my name will be included on my report to the authors and, if the manuscript is accepted for publication, my named report including any attachments I upload will be posted on the website along with the authors' responses. I agree for my report to be made available under an Open Access Creative Commons CC-BY license (http://creativecommons.org/licenses/by/4.0/). I understand that any comments which I do not wish to be included in my named report can be included as confidential comments to the editors, which will not be published.

Choose an item.

To further support our reviewers, we have joined with Publons, where you can gain additional credit to further highlight your hard work (see: https://publons.com/journal/530/gigascience). On publication of this paper, your review will be automatically added to Publons, you can then choose whether or not to claim your Publons credit. I understand this statement.

Yes Choose an item.
