# Supplementary material for: Genos: a human-centric genomic foundation model
Source: Gigascience. 2026 Jan 1;14:giaf132. doi: 10.1093/gigascience/giaf132 (PMC12755919; doi:10.1093/gigascience/giaf132)
Supplement: giaf132_Reviewer_2_Report_Original_Submission — Xiaofei Yang -- 10/16/2025 [file giaf132_reviewer_2_report_original_submission.pdf]

**Reviewer Report**

**Title: Genos: A Human-Centric Genomic Foundation Model**

**Version: Original Submission** **Date:** 10/16/2025

**Reviewer name: Xiaofei Yang**

**Reviewer Comments to Author:**

The Genos model represents an advancement in genomic foundation models, leveraging a highly optimized Mixture-of-Experts (MoE) architecture and novel components like RoPE with a high base frequency 50M and Grouped-Query Attention (GQA). This design addresses two major challenges in genomics: processing ultra-long sequences (up to 1M tokens) and achieving high efficiency via adaptive resource allocation.
I have following comments:
1. Performance Benefits of the MoE Architecture
The manuscript thoroughly emphasizes the runtime efficiency and scalability benefits afforded by the Mixture-of-Experts (MoE) architecture. However, a comprehensive assessment of MoE's contribution to the model's intrinsic predictive performanceâ€"independent of its efficiency gainsâ€"is currently lacking. Without this, the performance superiority over a conventional Transformer remains unproven.
The authors need to conduct a comprehensive ablation study focusing specifically on the MoE module. This study should compare the performance of the final Genos model (e.g., Genos-1.2B or Genos-10B) against an equivalent, densely connected Transformer model with a matching or comparable number of activated parameters per forward pass.
The results of this ablation should clearly quantify the performance lift (e.g., AUC or accuracy improvement) attributable directly to the sparse gating and routing mechanism of the MoE layer across a representative subset of the genomic benchmarks (e.g., short-range GB tasks and long-range LRB tasks). This will conclusively demonstrate that the MoE architecture is superior not only in efficiency but also in its ability to model complex genomic data.
2. Evaluation of Tissue-Specificity and Contextual Modeling
The model's strong performance is demonstrated on general genomic classification tasks. However, many critical downstream applications, such as the prediction of alternative splice sites, gene expression levels (RNA expression), and chromatin accessibility, are highly tissue- or cell-type specific. It remains unclear how Genos handles the inherent variability introduced by different biological contexts.
The authors need to address the model's capability to generalize or specialize in tissue-specific tasks. Specifically:
1. Data Strategy: Please detail whether the pre-training or fine-tuning datasets include cell/tissue-type labels or context-specific genomic features (e.g., cell-specific histone modifications). If so, elaborate on how this information was integrated into the model architecture or training process.
2. Performance Evaluation: The authors are requested to either:
a) Provide an evaluation of Genos's performance on a benchmark where tissue-specificity is the primary variable (e.g., predicting RNA expression across multiple ENCODE or GTEx tissues).

b) If direct evaluation is infeasible, provide a detailed discussion on how the model's design (e.g., long-range attention, MoE architecture) is theoretically equipped to capture and utilize tissue-specific regulatory information when fine-tuned on contextualized datasets.
Demonstrating or thoroughly discussing the model's capacity to handle the complexities of tissue-specific regulatory landscapes is essential for establishing its utility in precision medicine and functional genomics.
3. Intergenic Content Filtering and Downstream Task Performance
The data preprocessing strategy described in Section 2.1 introduces a selective filtering mechanism for intergenic regions (e.g., excluding content beyond 5,120 bp from gene boundaries in 8,192 bp fragments). While this concentrates pre-training on gene-proximal elements, it omits distal intergenic content.
For certain tasks, such as the classification of Transposable Elements (TEs) or the distinction of true splice sites from non-functional sequences, the excluded distal intergenic content serves as an essential negative training data source to reduce false positives. The authors should provide an evaluation or detailed discussion on the potential impact of this intergenic filtering strategy on the final performance of such related tasks, particularly those relying heavily on strong background modeling. A theoretical justification for why the model's capacity overcomes this data exclusion would be helpful.
4. Comparative Benchmarking against Specialized Variant Effect Models
The performance evaluation presented in the manuscript (e.g., Table 4) validates the multimodal Genos model's competence on the KEGG task. However, to fully establish the model's predictive superiority in variant effect prediction, the current comparison lacks a direct benchmark against state-of-the-art models specialized in this domain.
The authors are strongly encouraged to include a direct comparative evaluation against at least one leading, large-scale model specifically designed for variant effect prediction, such as AlphaGenome. This comparison, ideally on the KEGG task or a relevant variant effect benchmark, would clearly demonstrate whether the novel multimodal architecture offers a quantifiable predictive advantage over dedicated, specialized genomic foundation models. If a full re-evaluation is infeasible, a detailed discussion justifying Genos's expected superiority over such specialized models is required.

**Methods**

Are the methods appropriate to the aims of the study, are they well described, and are necessary controls included? Choose an item.

**Conclusions**

Are the conclusions adequately supported by the data shown? Choose an item.

**Reporting Standards**

Does the manuscript adhere to the journal's guidelines on [minimum standards of reporting?] Choose an item.

Choose an item.

**Statistics**

Are you able to assess all statistics in the manuscript, including the appropriateness of statistical tests used? Choose an item.

**Quality of Written English**

Please indicate the quality of language in the manuscript: Choose an item.

**Declaration of Competing Interests**

Please complete a declaration of competing interests, considering the following questions:

- Have you in the past five years received reimbursements, fees, funding, or salary from an organisation that may in any way gain or lose financially from the publication of this manuscript, either now or in the future?
- Do you hold any stocks or shares in an organisation that may in any way gain or lose financially from the publication of this manuscript, either now or in the future?
- Do you hold or are you currently applying for any patents relating to the content of the manuscript?
- Have you received reimbursements, fees, funding, or salary from an organization that holds or has applied for patents relating to the content of the manuscript?
- Do you have any other financial competing interests?
- Do you have any non-financial competing interests in relation to this paper?

If you can answer no to all of the above, write 'I declare that I have no competing interests' below. If your reply is yes to any, please give details below.

I have no competing interests

I agree to the open peer review policy of the journal. I understand that my name will be included on my report to the authors and, if the manuscript is accepted for publication, my named report including any attachments I upload will be posted on the website along with the authors' responses. I agree for my report to be made available under an Open Access Creative Commons CC-BY license (http://creativecommons.org/licenses/by/4.0/). I understand that any comments which I do not wish to be included in my named report can be included as confidential comments to the editors, which will not be published.

Choose an item.

To further support our reviewers, we have joined with Publons, where you can gain additional credit to further highlight your hard work (see: https://publons.com/journal/530/gigascience). On publication of this paper, your review will be automatically added to Publons, you can then choose whether or not to claim your Publons credit. I understand this statement.

Yes Choose an item.
